# Synthesis, Structure, and Reactivity of Binaphthyl Supported Dihydro[1,6]diazecines

**DOI:** 10.3390/molecules24173098

**Published:** 2019-08-26

**Authors:** Miran Lemmerer, Michael Abraham, Bogdan R. Brutiu, Alexander Roller, Michael Widhalm

**Affiliations:** 1 Institute of Organic Chemistry, University of Vienna, Währinger Straße 38, 1090 Wien, Austria; 2 Institute of Chemical Catalysis, University of Vienna, Währinger Straße 38, 1090 Wien, Austria; 3 Institute of Inorganic Chemistry, University of Vienna, Währinger Straße 42, 1090 Wien, Austria

**Keywords:** 2,2′-diamino-1,1′-binaphthyl, ring closing metathesis, heterocycle, diaza-macrocycle, dihydroxylation, epoxidation, ring contraction, rearrangement

## Abstract

A short approach to chiral diaza-olefines from protected 2,2′-diamino-1,1′-binaphthyl is presented. *Cis*- and *trans*-olefines can be selectively obtained by twofold *N*-allylation followed by RCM or by bridging a 2,2′-diamino-1,1′-binaphthyl precursor with *trans*-1,4-dibromo-2-butene. Deprotection afforded *cis*- and *trans*-dihydro[1,6]diazecines **1** in 58 and 64% overall yield. The reactivity of the but-2-ene-1,4-diyl fragment was investigated yielding corresponding epoxides, diols, and mono- and dibromo products. In several cases rearrangements and participation of the proximate *N*-Boc group was observed. In no case could allylic substitution be accomplished. From 13 compounds X-ray structure analyses could be obtained.

## 1. Introduction

**Figure 1:**
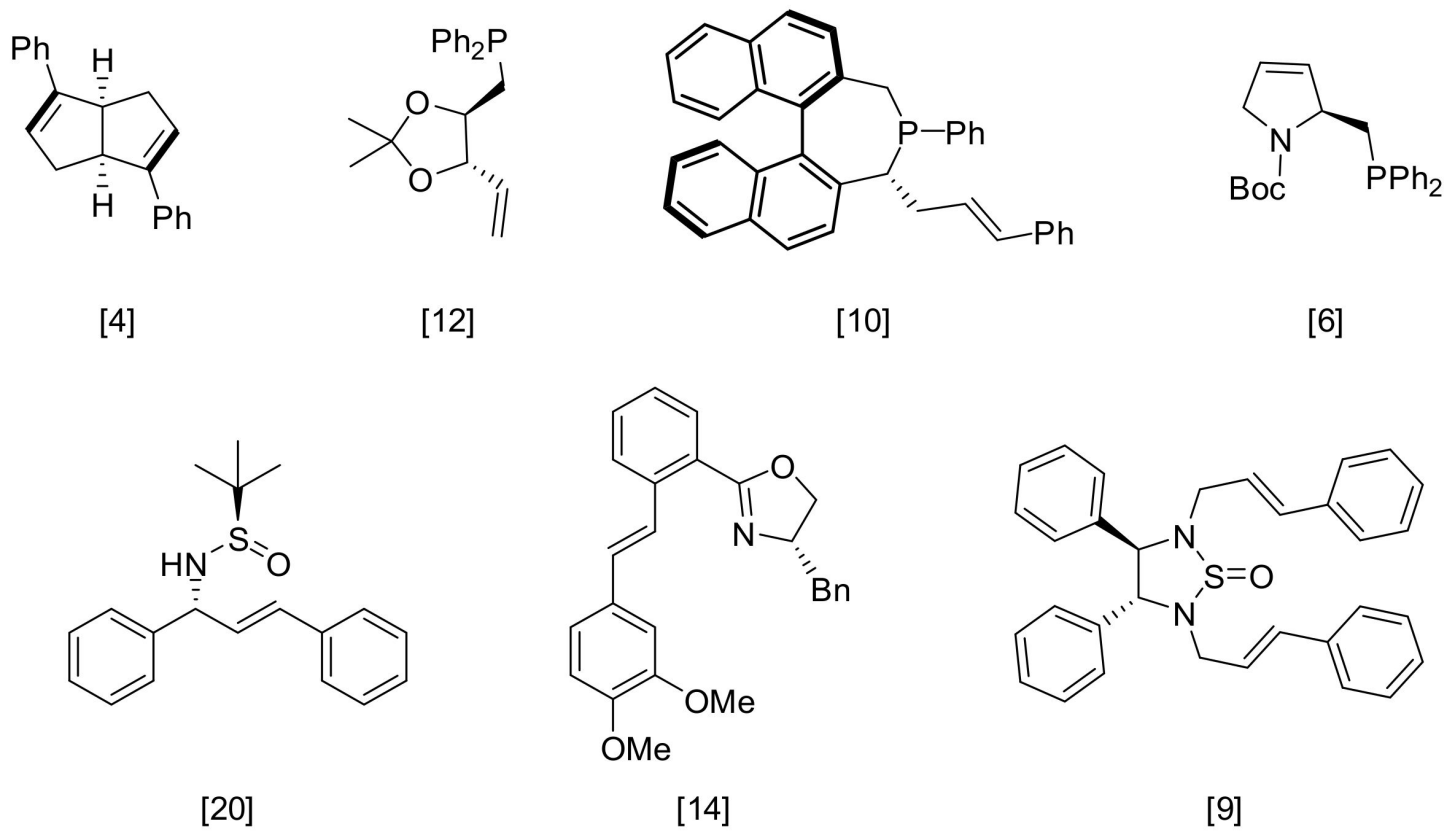
Selected mono- and diolefine ligands previously applied in asymmetric catalysis.

Monoolefine and diolefine ligands are often key players in homogeneous catalysis and have found various applications in asymmetric transformations [1,2]. The preferred structures are either rigid, based on bicyclic diene skeletons [3,4,5], semi-rigid, consisting of a mono-ene as part of a cycle which is linked to P [6,7] or S [8,9] functionalities as second coordination site, or flexible with the olefin part being a freely rotating *pending side arm* attached at a chiral back bone [10,11,12,13,14,15,16,17,18,19,20]. Some examples showing structural diversity are depicted in Figure 1.

The requirement for an efficient chiral ligand in transition metal catalysis is its ability to form only a few, conformative stable diastereomeric intermediates during the catalytic cycle. Ideally, these show highly differing stability and/or traversing transition states with significantly different activation energy on the reaction path to product enantiomers. This is usually fulfilled if stable chelate structures are involved. The challenge in catalyst design is to produce molecules with two coordination centres with a sufficiently large chiral cavity and *tuned* rigidity to form stable substrate complexes best as a single conformer. As the search for proper catalysts is a largely empirical and time consuming process, easy access to ligand libraries to be tested is desired. To this end, structural modification should be done at a late stage of the synthesis, preferably as the last step.

**Figure 2:**
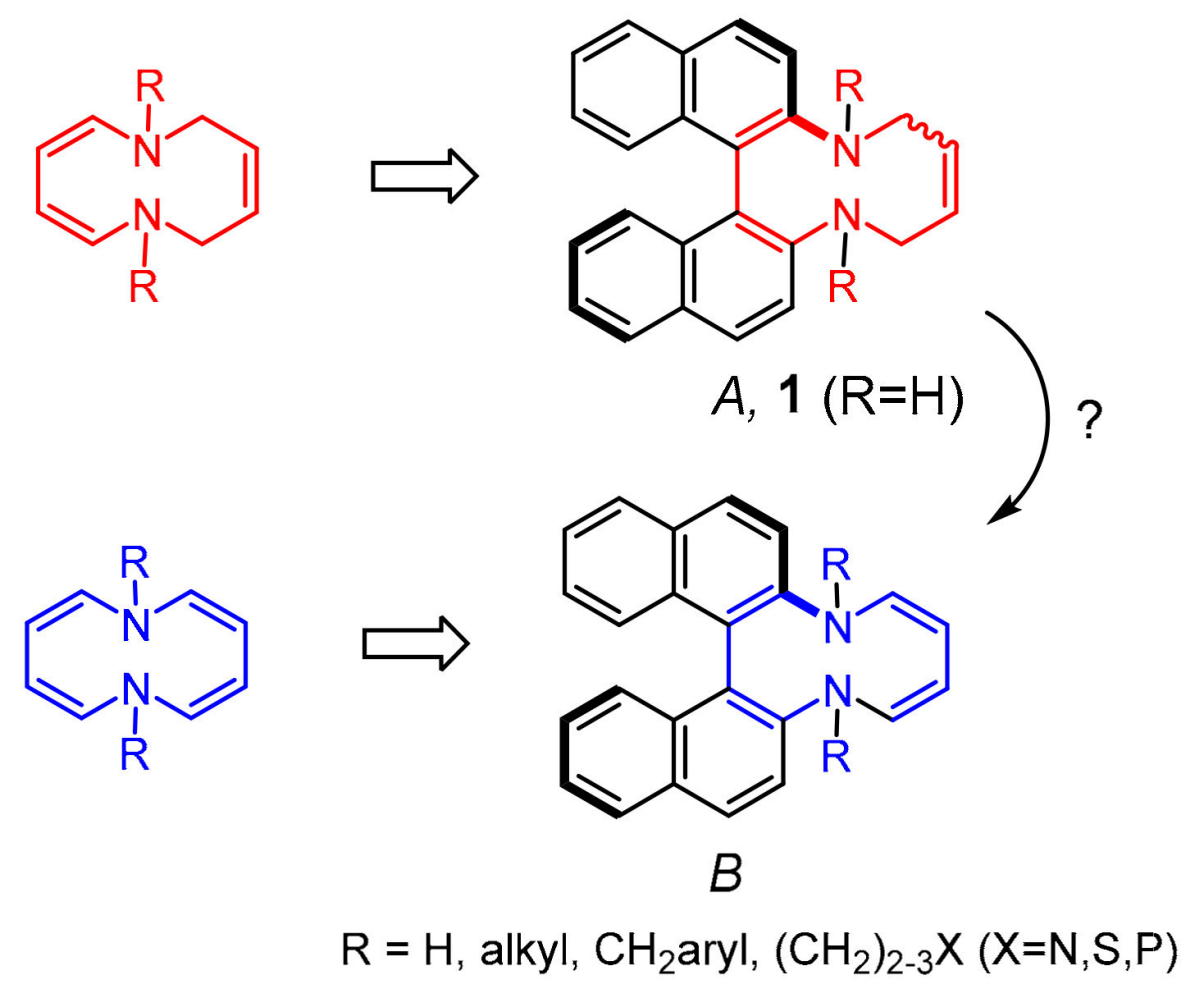
Chiral diazaheterocycles based on the 1,1′-binaphthyl skeleton.

As a further extension of ligand design, we therefore considered the incorporation of an atropomeric biaryl unit as part of a cycloolefine *A* or –diolefine moiety *B* (Figure 2). This would place corresponding olefine complexes in a chiral environment with a variable degree of conformative freedom depending on the size and rigidity of the perimeter. Introduction of *N*-alky or –methylaryl substituents will fine-tune steric interactions. In the case of monoolefine *A,* various *N*-substituents also containing heteroatoms (sulphur or phosphorus functional groups preferred) could be introduced in the final step to act as further potential coordination sites. The aim of the present investigation was to synthesize the simplest candidate **1** (R=H) through bridging of 2,2′-diamino-1,1′-binaphthyl, exploring stereochemistry and reactivity [21].

## 2. Results and Discussion

**Scheme 1:**
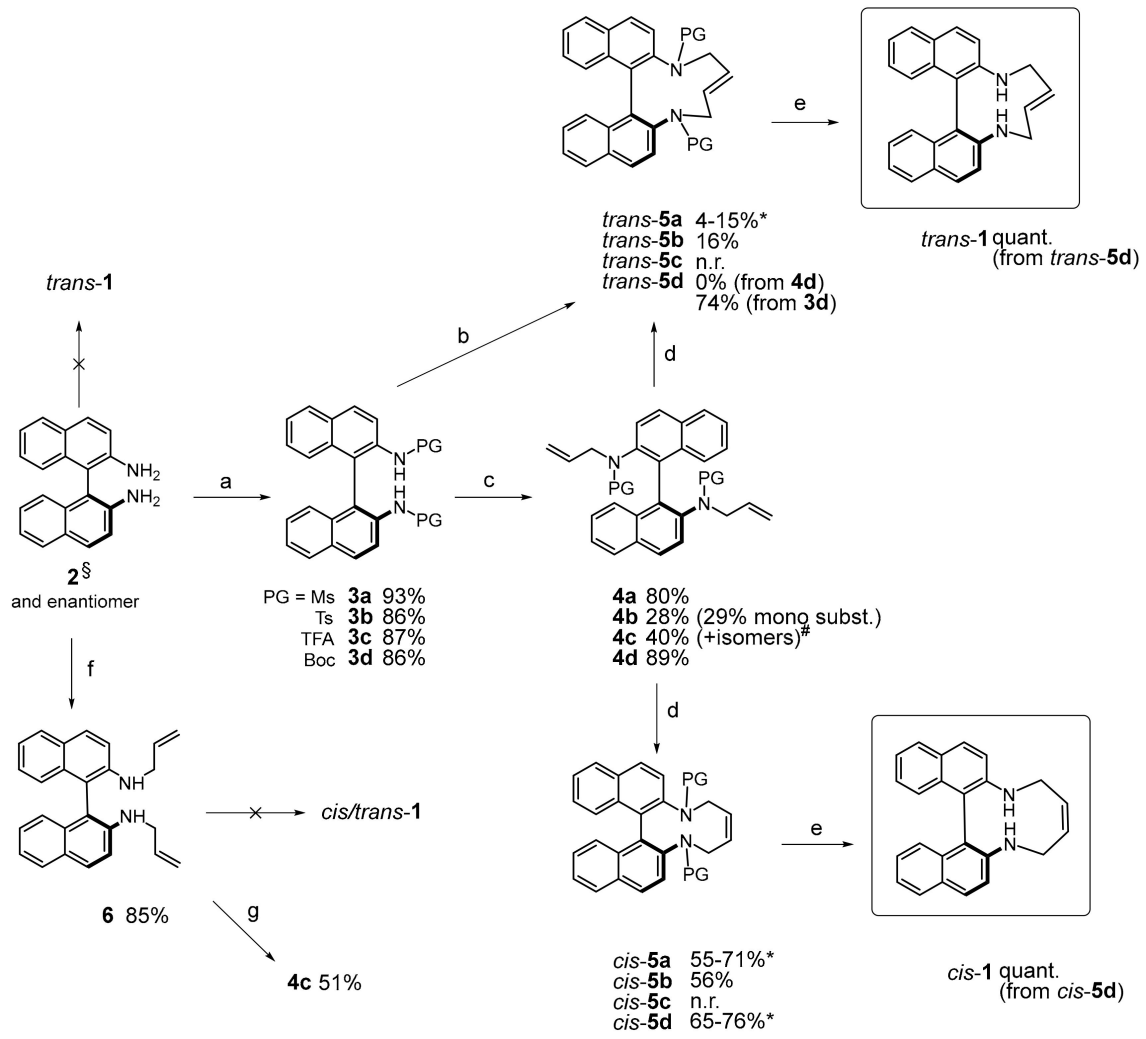
Synthesis of *cis*- and *trans*-**1**. (a) MsCl or TsCl, Py/DCM, r.t. (**3a**, **3b**), trifluoroacetic anhydride (TFAA), Na_2_CO_3_, THF, r.t. (**3c**), Li hexamethyldisilazide (LiHMDS), Boc_2_O, THF, 0 °C→r.t. (**3d**); (b) NaH, *trans*-1,4-dibromobut-2-ene, THF, 0 °C→r.t., (c) allylbromide, K_2_CO_3_, MeCN, 85 °C (**4a**, **4b**), allylbromide, NaH, DMF, 0 °C→r.t. (**4d**), (d) 10 mol% catalyst (*see text*) DCM, 40 °C, syringe pump, (e) trifluoroacetic acid (TFA), DCM, 0→4 °C, (f) allyl alcohol, Pd(OAc)_2_, Ph_3_P, Ti(O*i*Pr)_4_, benzene, 50 °C, (g) TFAA, Et_3_N, 4-dimethylaminopyridine (DIMAP), DCM, r.t. * Yield depending on reaction condition and metathesis catalyst applied. ^#^ see text. ^§^ *Note:* The synthesis was conducted with racemic starting material and consequently all products are racemic, too; only the enantiomer with (*S*)_axial_ configuration is depicted.

For the synthesis of **1** in the beginning, a seemingly simple cyclization step of diaminobinaphthyl **2** was considered using *trans*-1,4-dibromo-2-butene or *trans*-1,4-dihydroxy-2-butene (Scheme 1). Unfortunately, only inseparable mixtures of, and in part oligomerized, products were obtained. Alternatively, olefin ring closing metathesis (RCM) of bis-*N*-allyl substrate **6** with Grubbs I, Grubbs II, Grubbs-Hoveyda II, and Schrock′s catalyst was attempted, which was previously successfully applied for substrates with unprotected NH functionality [22,23,24,25]. With none of these catalysts did a cyclization of **6** take place, neither at r.t. nor elevated temperature.

Protection of NH was therefore envisaged and suitable *N*-protecting groups (PG) were installed before the RCM step [26]. Diaminobinaphthyls **3a**–**d** with *N*-Ms [27], *N*-Ts [28], *N*-TFA, and *N*-Boc groups [29] were synthesized under standard conditions and obtained in good yield (86–93%). While in the subsequent allylation **3a** and **3d** performed well, yielding the disubstituted products **4a** and **4d** in 80% and 89%, respectively, the substitution of Ts-protected amine **3b** proceeded slowly affording 28% of **4b** along with 29% of the mono-allylated product.

We were pleased to discover that in the case of **3d** the reactivity could be effectively controlled through proper choice of solvent. In THF, mono-allylation exclusively took place (71%), while on the other hand 89% of diallyl product **4d** was obtained in DMF.

**Figure 3:**
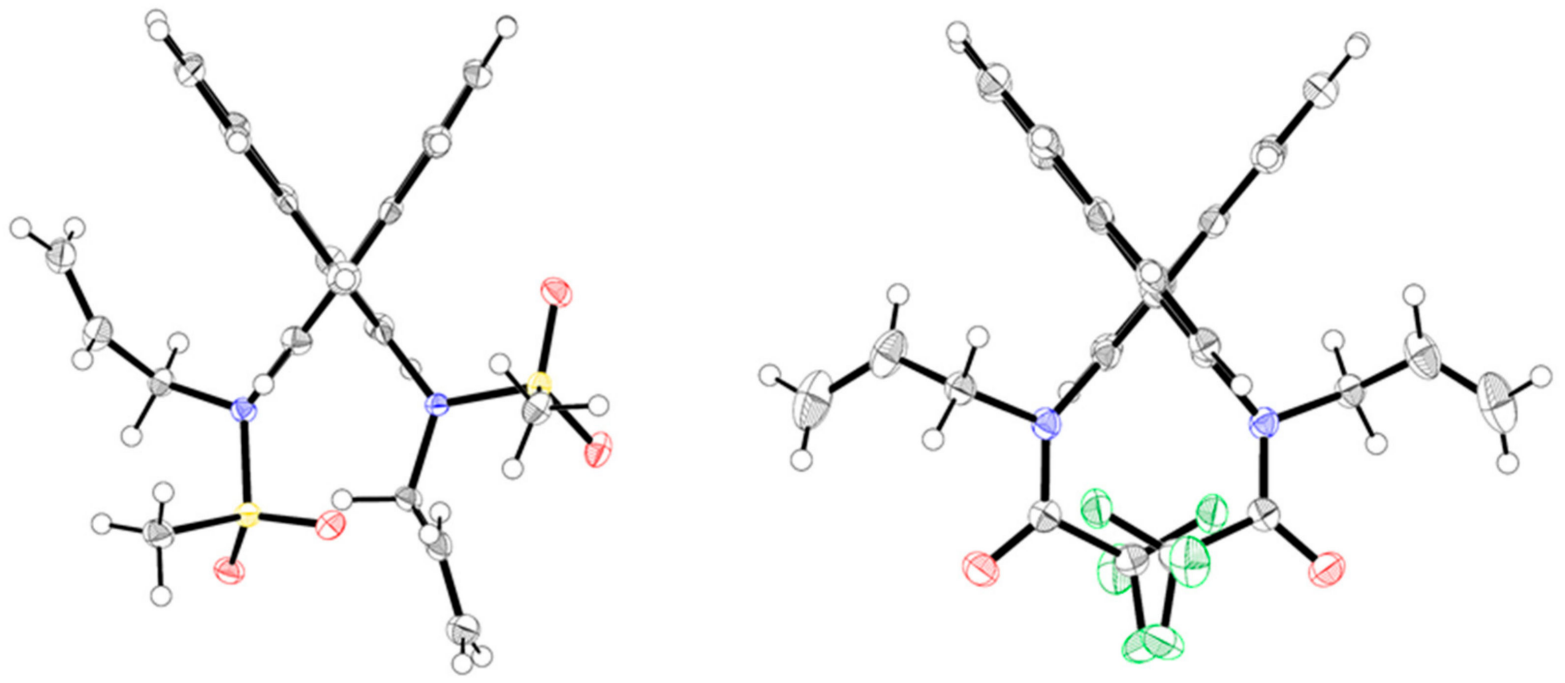
Crystal structure of **4a** (left side) and **4c** (right side). *Note:* To facilitate visual comparison of structures, all compounds are depicted with (*S*)_axial_-configuration and viewing along the binaphthyl axis.

The allylation of bis(trifluoro)acetamide **3c** proceeded slowly at r.t., yielding only monosubstitution product (DMF, NaH). Conducting the reaction at reflux (48 h) in acetonitrile in the presence of K_2_CO_3_ resulted in a complex mixture, only separable in part by chromatography. Two bands were isolated, each of which contained at least three compounds, two with *C*_2_ symmetry and one with *C*_1_ symmetry as evidenced by ^19^F-NMR (see Supplementary Materials). Fortunately, one component of fraction 2 crystallized and X-ray crystallography confirmed the expected structure **4c** (Figure 3). Re-dissolving the crystalline material in CDCl_3_ gave identical spectra as before crystallization, supporting the assumption of three interconverting species in equilibrium. The same mixture was obtained as the sole product from diallyl compound **6** upon treatment with TFAA at r.t. The solution structure of neither of these compounds, nor of those present in the first fraction could be elucidated to date. All fractions gave the same HRMS and contain isomers of **4c**.

For the RCM step of **4a**–**d** commercially available catalysts Grubbs I, Grubbs II, and Grubbs-Hoveyda II were tested [30]. It was found that the influence of the type of catalyst on reactivity was moderate, but instead a pronounced impact of the nature and bulkiness of PG on reactivity and *cis/trans* selectivity was observed. While a complex mixture was formed from **4c** with no clear evidence for formation of a cyclic product **5c** [31], products with *cis*-geometry dominated (**5a**,**b**) or were formed exclusively (76% of *cis*-**5d** with Grubbs I). Having developed a synthetic route with high yields and remarkable selectivity with PG = Boc, the synthesis **2** → **3d** → **4d** → *cis*-**5d** → *cis*-**1** was chosen for subsequent investigations. Gratifying, the geometric isomer *trans*-**5d** was selectively accessible in 64% from **3d** and *trans*-1,4-dibromo-2-butene in one step. The deprotection under standard conditions proceeded smoothly, affording *cis*- and *trans*-**1** in quantitative yield.

The bulkiness of protecting groups effectively controlled the stability of conformers arising through rotation around the naphthyl-*N* bond. In ^1^H-NMR spectra significant line broadening of allyl moieties of **4a** and **4b** was observed, particularly pronounced for **4d**.

**Figure 4:**
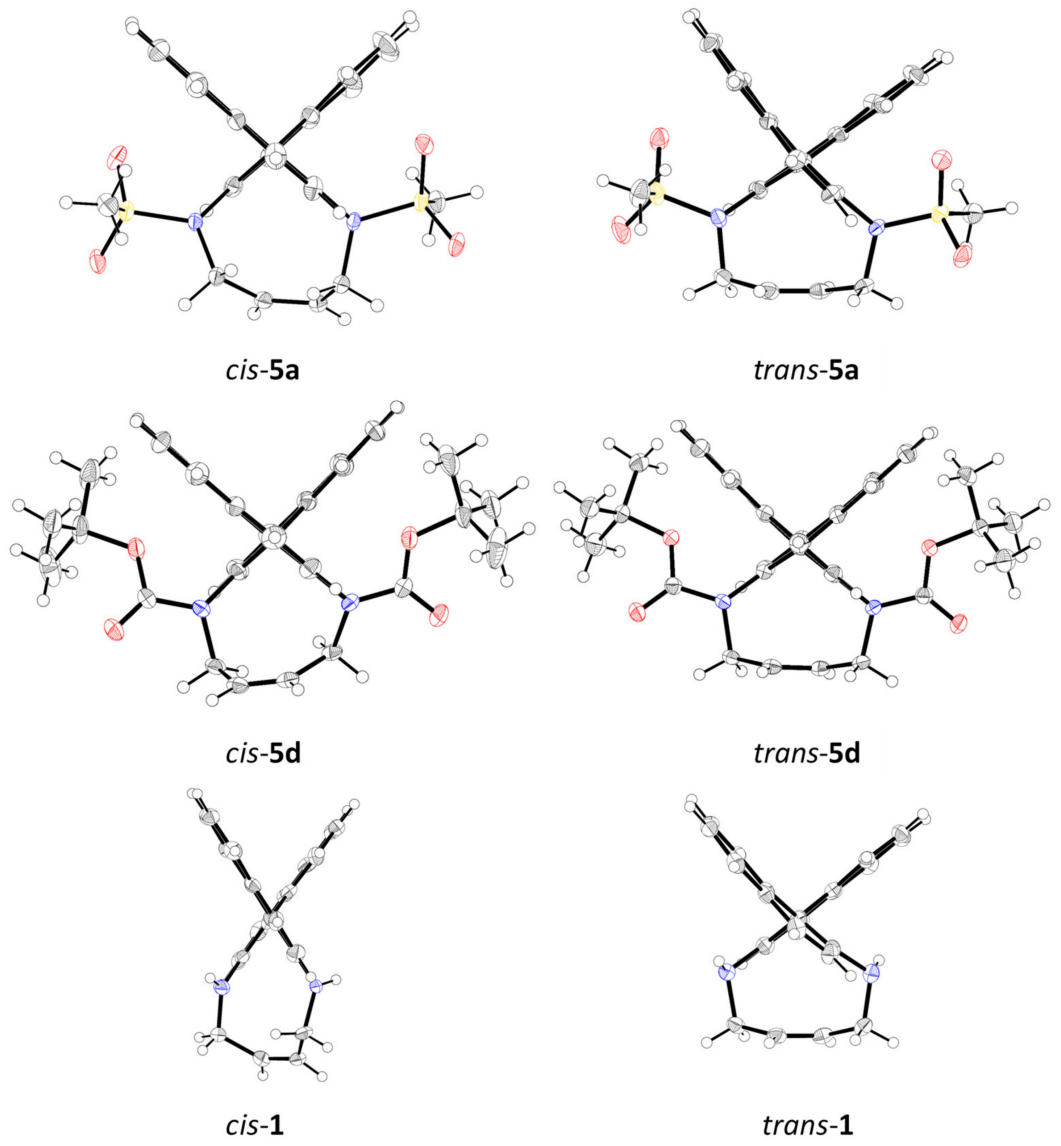
Crystal structures of *N*-protected diazacycles *cis*- and *trans*-**5a** and **5d** and deprotected cycles *cis*- and *trans*-**1**. See also note in Figure 3.

Solid state structure of *N*-protected cycles *cis*- and *trans*-**5a** and **5d** as well as cycles with free NH *cis*- and *trans*-**1** did not show evidence for severe steric strain (Figure 4). Double bonds only display small deviations from planarity (maximum 5.2° for *trans*-**1**) as a consequence of widely unrestricted rotability of the binaphthyl bond. Most striking are differences between *cis*- and *trans*-**1** with Ar-Ar angles of 65.2° and 98.9° and *N-N* distances of 3.04 Å and 4.31 Å, respectively. In *trans*-**5d**, as well as in *trans*-**1** intermolecular hydrogen bonds were observed (C-H∙∙∙∙O and N∙∙∙∙H, respectively see Supplementary Materials) and intramolecular C-H∙∙∙∙π-ring interactions in *trans*-**5d**.

*Cis*- and *trans*-**1** was completely stable in toluene even when heated to 100 °C which is in contrast to corresponding [1,6]dioxecine which could not be trapped due to its readiness to undergo Claisen rearrangement [32,33].

**Scheme 2:**
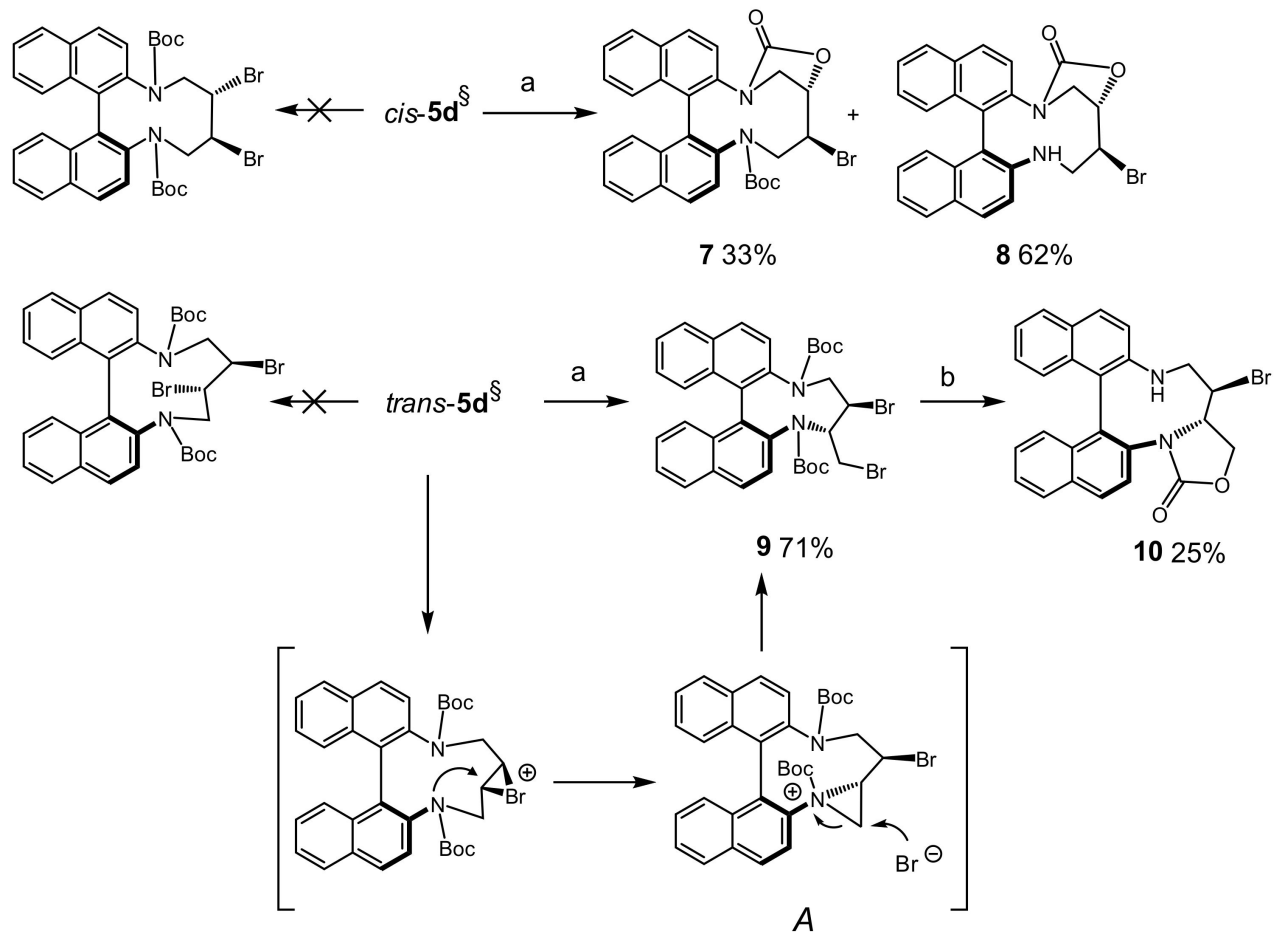
Bromination of *cis*- and *trans*-**5d.** (a) Br_2_, DCM, 0 °C→r.t. (b) TFA, DCM. ^§^ See note in Scheme 1.

**Figure 5:**
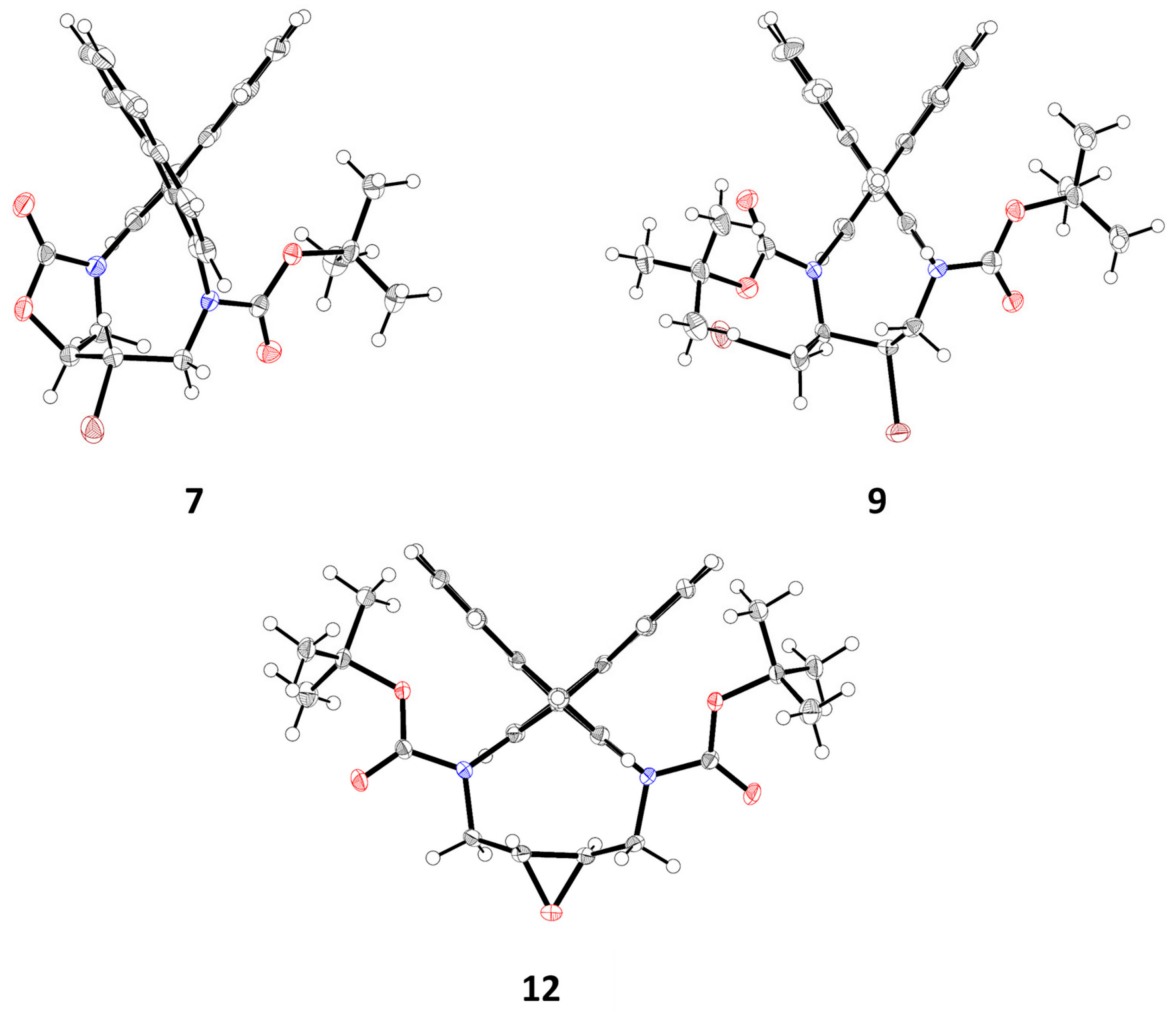
Crystal structure of **7**, **9**, and **12.** See also note in Figure 3.

The reactivity of intermediates **5d** was investigated next. Reaction of *cis*-**5d** with bromine did not give the expected dibromide by simple *trans* addition (Scheme 2). Instead formation of a cyclic carbamate **7** was observed, obviously formed through attack of the Boc group on the bromonium ion [34]. This step is facilitated as the *tert*-butyl cation is trapped by bromide, eventually forming some amount of isobutene with liberation of HBr which removes the second Boc group to give **8** as a sequence product. Racemic **7** crystallized in a chiral space group (see Supplementary Materials). From a non-racemic crystal relative configurations were determined as being *10S*, *11S*, in a binaphthyl with (*S*)_ax_ configuration. While **7** exists as a single geometric form, two conformers of **8** were detected in an approximate ratio of 60:40 (^1^H-NMR). Also *trans*-**5d** did not afford the 2,3-dibromide; instead, ring contracted product **9** was isolated in 71% yield. The geometry of **9** was in agreement with HRMS and confirmed by X-ray structure analysis (Figure 5).

The ^1^H-NMR spectrum, even from the crystallized compound, was a complex which was attributed interconverting conformers. A *N*-boc aziridinium cation *A* is suggested as an intermediate as a similar rearrangement was reported by Paquette et al. [35]. The reaction is rather slow and required more than 48 h to complete. Attempts to cleave the Boc groups proceeded only with an excess of TFA and resulted in a mixture of two products, both without Boc groups (^1^H-NMR). One was identified as cyclic carbamate **10**.

**Scheme 3:**
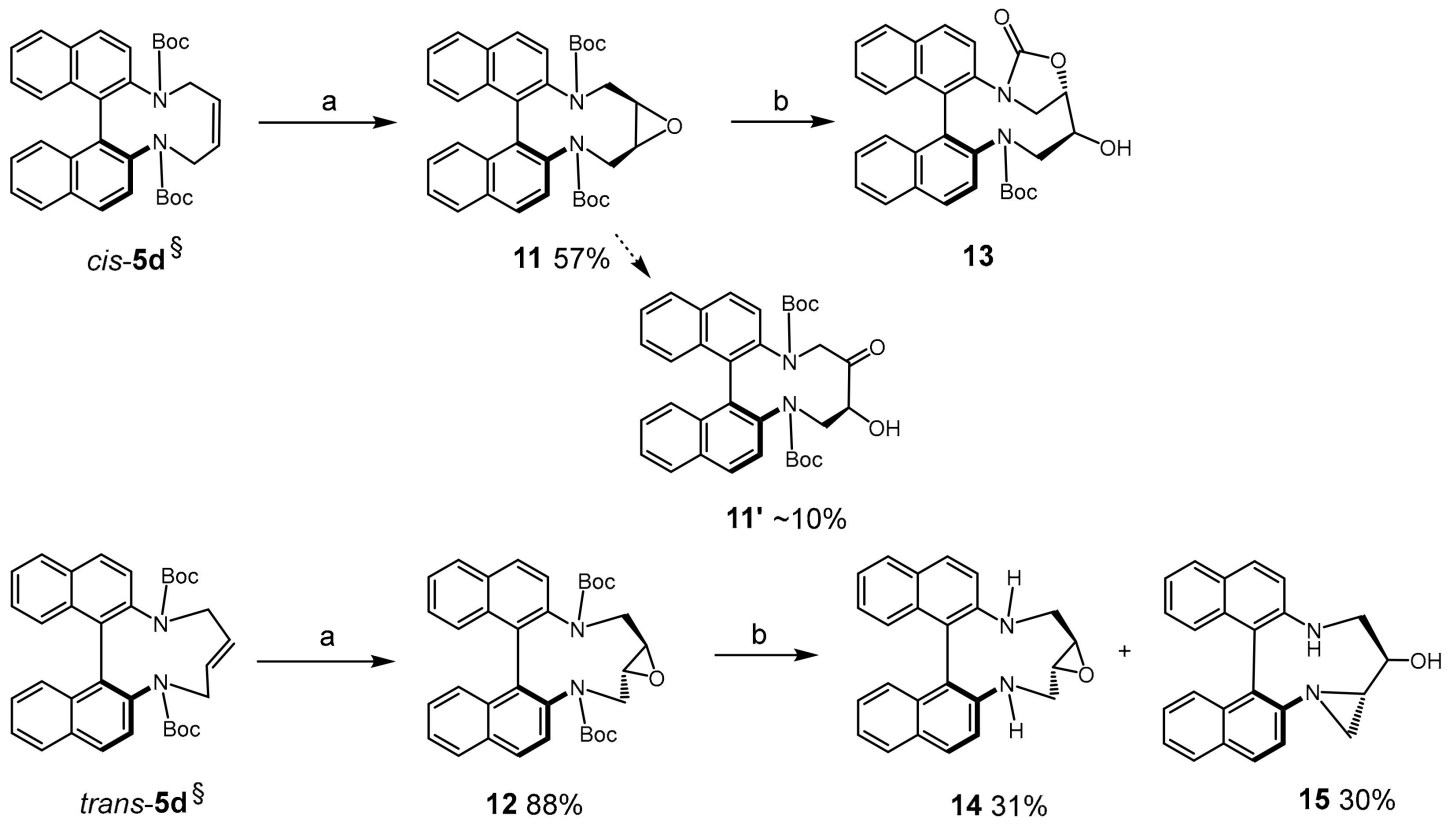
Epoxidation of *cis*- and *trans*-**5d.** (a) *meta*-chloroperbenzoic acid (*m*-CPBA), DCM. (b) TFA, DCM. ^§^ See note in Scheme 1.

Epoxidation of *cis*- and *trans*-**5** (Scheme 3) proceeded with both when employing *m*-CPBA under standard conditions [36], however considerably faster with the *trans*-substrate yielding **11** (57%) and **12** (89%), respectively. During prolonged reaction time, increasing amounts of hydroxyketone **11′** (10–30%) were formed. X-ray structure analysis confirmed the geometry of **12** and **11′**. Both compounds showed inter moleculer (**11′**, O-H∙∙∙O=C) or intra molecular interaction (**12**, C-H∙∙∙∙π-ring). For details see Supplementary Materials, Figures S14 and S16. Treatment of **11** with excess of TFA afforded cyclocarbamate **13** still carrying one Boc group. Since formation of 5-membered rings is in general faster, this structure appears to be more reasonable than the isomeric 6-membered carbamate and is in agreement with 2D-NMR. A similar transformation has been reported by Tietze et al. [37]. Attempted deprotection of **12** with TFA at r.t. yielded a mixture of **14** and **15**.

**Figure 6:**
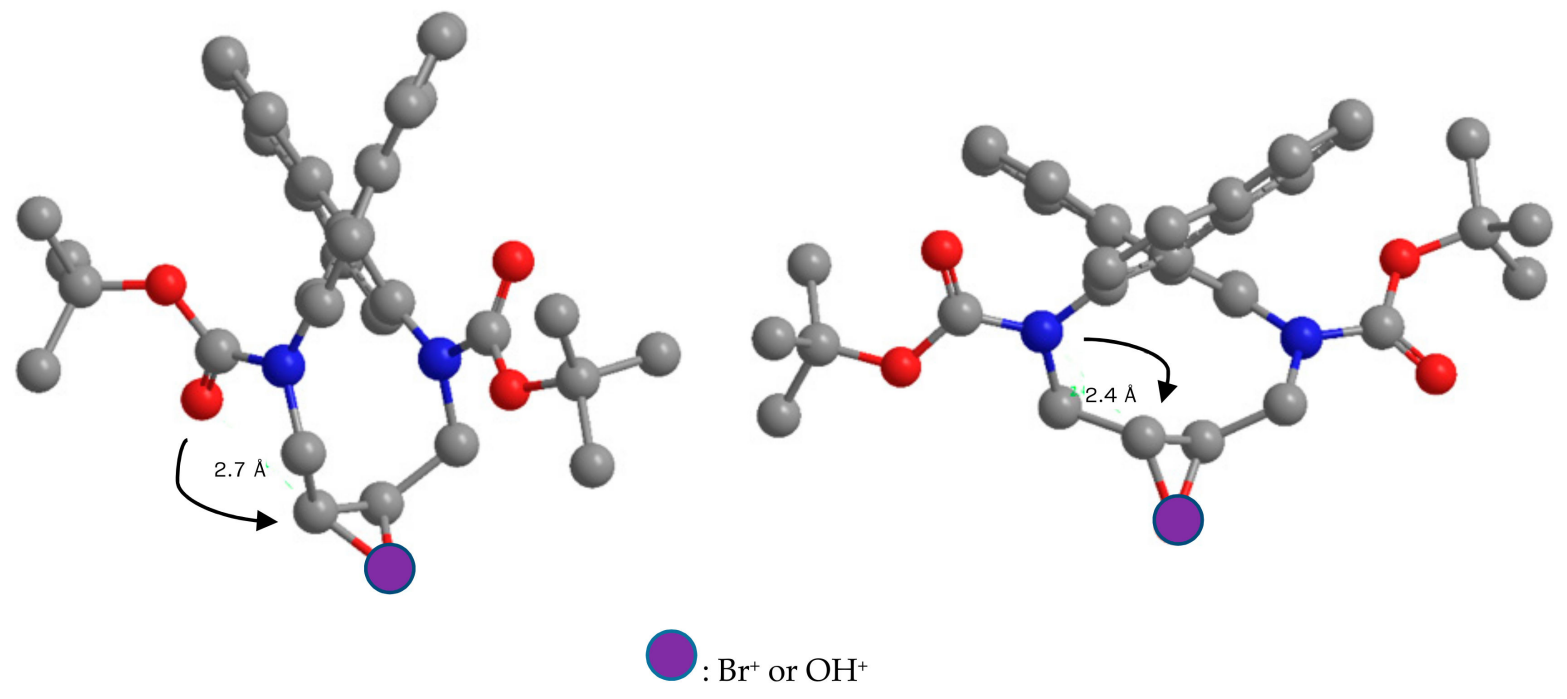
Graphic representation of postulated onium ions derived from *cis*- (left side) and *trans*-**5d** (right side).

Bromination and TFA-induced deprotection of epoxides show similar behavior, best explained with the presence of cyclic onium ions in both cases (Figure 6). Their reactivity might be controlled through conformation of the substrate with efficient shielding preventing intermolecular reactivity but favouring an intramolecular attack of the boc group. The *trans*-isomer of **5d** forms (protonated) epoxide **12** (and obviously also a cyclic bromonium ion) with local *C*_2_ symmetry, which will be attacked by N rather than by O as the Boc group is directed *outside* the perimeter (distance C-N: 2.4 Å) to give **9** via *A* (Scheme 2) and (presumably) a precursor of **15**. In both cases a *N*-boc-aziridinium ion might be a key intermediates. In contrast, the *cis*-isomer of **5d** may form an onium ion with better accessibility of the Boc carbonyl group yielding **7** and **13**, respectively (distance C-O: 2.7 Å).

**Scheme 4:**
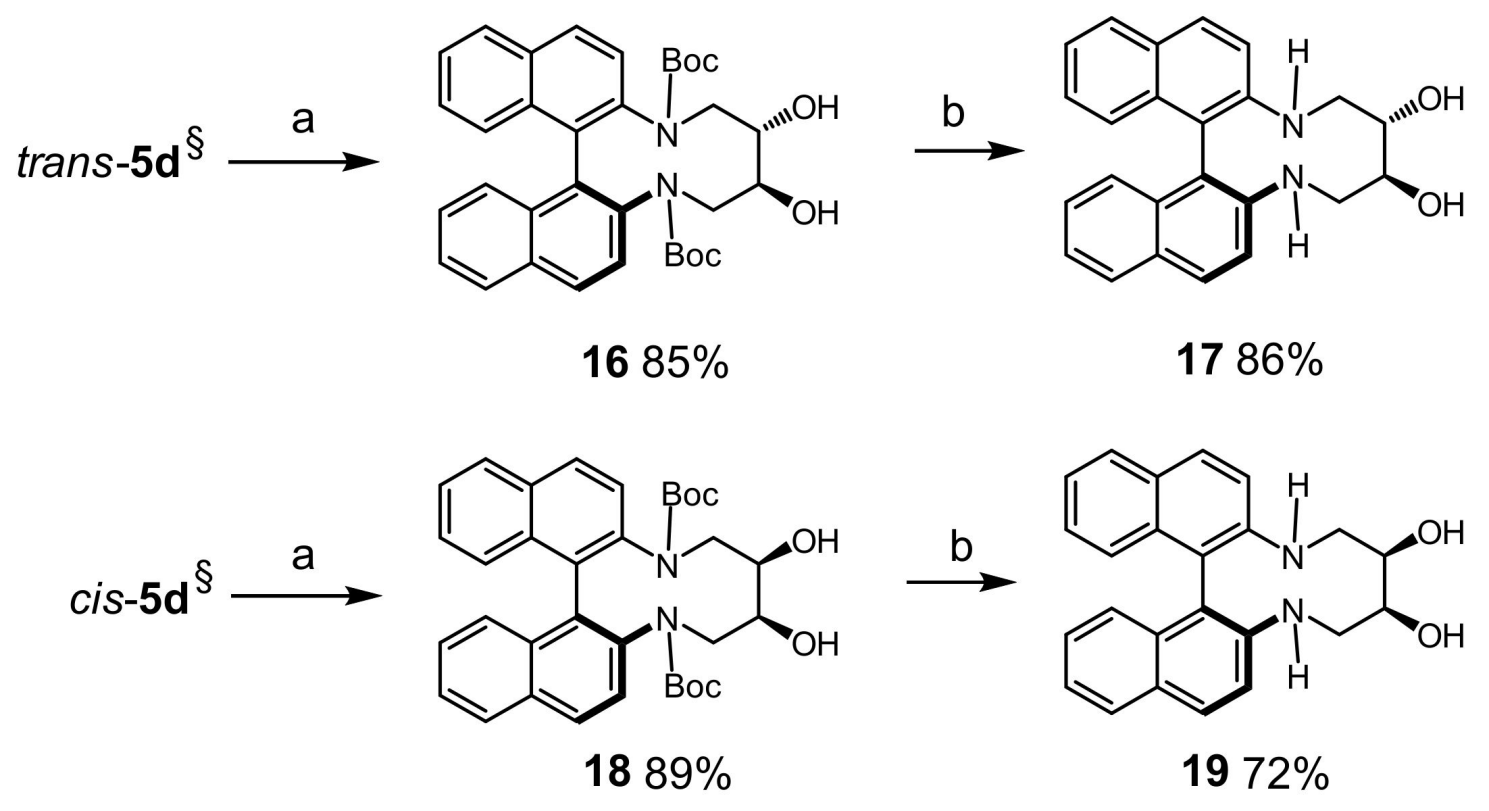
Dihydroxylation of *cis*- and *trans*-**5d.** (a) K_2_OsO_4_, NMO, DCM. (b) TFA, DCM. ^§^ See note in Scheme 1.

**Scheme 5:**
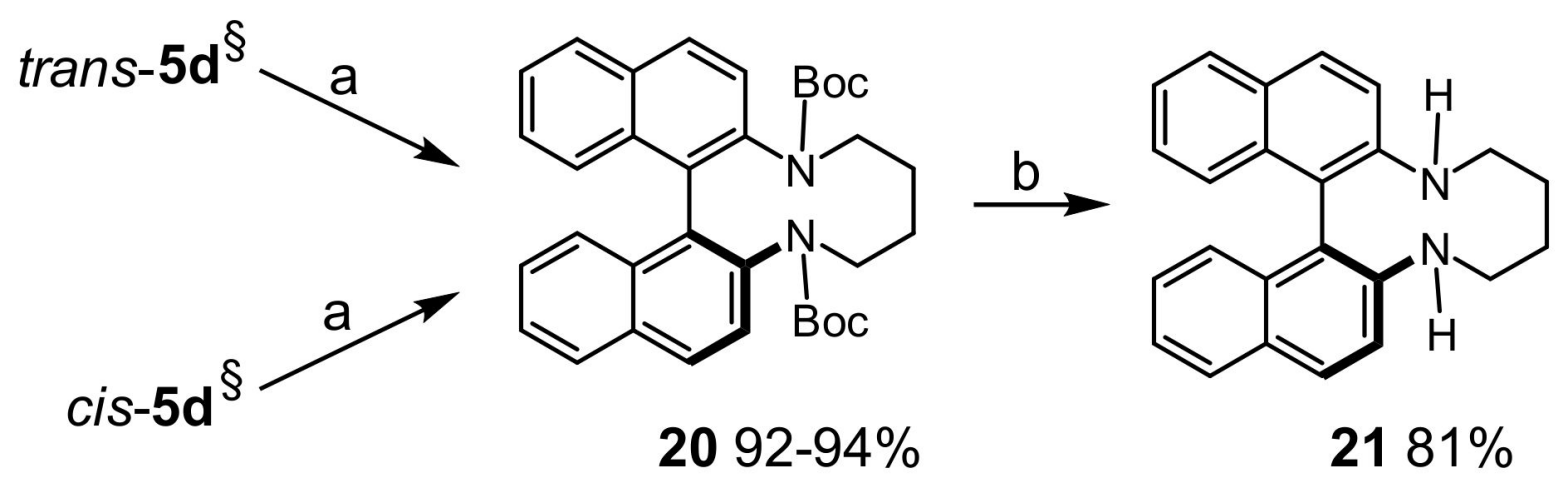
Hydrogenation of *cis*- and *trans*-**5d** (a) Pd/C, H_2_ (1 bar), THF. (b) TFA, DCM. ^§^ See note in Scheme 1.

The dihydroxylation of *cis*- and *trans*-**5d** under standard conditions (K_2_OsO_4_, *N*-methylmorpholino-*N*-oxide (NMO) afforded diols **16** and **18** and after deprotection dihydroxydiamines **17** and **19** in good yield. The hydrogenation of *cis*- or *trans*-**5d** yielded diamine **21** in two steps (Scheme 4 and Scheme 5). The X-ray structure of **21** was determined (see Supplementary Materials).

Treatment of unprotected substrates, *cis*- and *trans*-**1**, with bromine under various conditions produced inseparable mixtures of polybrominated products. ^1^H-NMR spectra showed formation of several compounds with up to four bromo substituents (also in position 6 and 6′ of the binaphthyl moiety).

Summarizing, a short synthetic route for two ten-membered chiral diaza-macrocyles, *cis*- and *trans*-**1**, in 3 and 4 steps from 2,2′-diamino-1,1′-binaphthyl (58-64% overall yield) was developed and the reactivity of Boc-protected precursors towards bromine, *m*-CPBA, K_2_OsO_4_/NMO, and H_2_/Pd was investigated. In several cases, rearranged products could be isolated and characterized. Crystal structures of target compounds and various intermediates were determined.

## 3. Materials and Methods

### 3.1. General Considerations

Melting points: Kofler melting point apparatus, uncorrected. NMR: recorded at 400.27 MHz (^1^H) and 100.66 MHz (^13^C), respectively, or at 600.25 MHz (^1^H) and 150.95 MHz (^13^C), respectively, on a Bruker AVIII400 or AVIII600 spectrometer. Chemical shifts δ were reported in ppm; for ^1^H rel. to (residuals non-deuterated) solvent signals (chloroform-d or DMSO-d6: 7.26 or 2.50 ppm, respectively), for ^13^C to CDCl_3_ or (CD_3_)_2_SO at 77.00 or 39.52 ppm, respectively. Coupling patterns were designated as s(inglet), d(oublet), t(riplet), q(uartet), m(ultiplet), ps(eudo), and br(oad). ^13^C{^1^H}-NMR spectra are recorded in a J-modulated mode; signals are assigned as C, CH, CH_2_, and CH_3_. HRMS: ESI (maXis ESI-Qq-TOF mass spectrometer, Bruker Daltonics, Bremen, Germany), or EI (Bruker, 70 eV).

Heptane fraction (PE), dichloromethane (DCM), and ethyl acetate (EtOAc) were distilled, absolute THF from sodium benzophenone ketyl, dichloromethane (DCM), DMF, and acetonitrile from CaH_2_; Li hexamethyldisilazide (LHMDS) was used as a 1.0 molar solution in THF. All the other chemicals were analytical grade and used without further purification. Preparative medium pressure chromatography (MPLC) was performed on an Isolera One chromatograph (Biotage) applying a solvent gradient using self-packed cartridges (SiO_2_, 40-63 µm). Reported procedures have been followed to obtain 2,2′-diamino-1,1′-binaphthyl (**2**) [38] and di-*tert*-butyl [1,1′-binaphthalene]-2,2′-diyldicarbamate (**3d**) [29].

### 3.2. Synthesis

**(*E*)-11,12,15,16-Tetrahydrodinaphtho [2,1-b:1′,2′-d]**[1,6]**diazecine** (**trans**-**1**): A solution of *trans*-**5d** (67 mg, 0.12 mmol) in DCM (3 mL) was cooled to 0 °C and an excess of TFA (1.5 mL) was added. The mixture was stirred for 2 h and then kept at 4 °C overnight. The reaction was quenched by careful addition of saturated NaHCO_3_ solution (10 mL) and extracted with DCM. The organic layer was dried (Na_2_SO_4_) and the solvent was evaporated at reduced pressure, affording 40 mg (99%) of *trans*-**1** as colorless crystals; m.p.: 204–207 °C. ^1^H-NMR δ = 7.99 (d, *J* = 8.8 Hz, 2H); 7.91 (d, *J* = 8.2 Hz, 2H); 7.48 (d, *J* = 8.8 Hz; 2H); 7.43 (ddd, *J* = 8.2, 6.5, 1.7 Hz, 2H); 7.31 (ddd, *J* = 8.4, 6.6, 1.3 Hz, 2H); 7.27 (dm, *J* = 8.4 Hz, 2H); 4.67–4.76 (m, 2H); 3.42 (dm, *J* = 13.1 Hz, 2H); 3.22 (dm, *J* = 13.1 Hz, 2H); ~2.8 (br.s, 2H). ^13^C-NMR δ = 144.5 (C); 133.6 (C); 130.8 (C); 129.6 (CH); 128.7 (C); 128.3 (CH); 127.8 (CH); 127.3 (CH); 126.6 (CH); 125.0 (CH); 124.9 (CH); 52.2 (CH_2_). HRMS: calcd for C_24_H_21_N_2_ [M + H]^+^: 337.1698; found: 337.1696.

**(*Z*)-11,12,15,16-Tetrahydrodinaphtho[2,1-b:1′,2′-d]**[1,6]**diazecine** (**cis-1**)**:** The same procedure was applied as given for *trans*-**1**; yield: 34 mg (99%, colorless crystals, 0.1 mmol scale); m.p.: 184–185 °C. ^1^H-NMR δ = 7.90 (d, *J* = 8.7 Hz, 2H); 7.82 (br.d, *J* = 8.0 Hz, 2H); 7.35 (d, *J* = 8.8 Hz, 2H); 7.29 (ddd, *J* = 8.1, 6.6, 1.5 Hz, 2H); 7.21 (ddd, *J* = 8.5, 6.6, 1.4 Hz, 2H); 7.17 (dm, *J* = 8.5 Hz, 2H); 5.92–6.00 (m, 2H); 3.73–3.91 (m, 4H); 3.30 (br.s, 2H). ^13^C-NMR δ = 145.8 (C); 134.0 (C); 132.7 (CH); 129.7 (CH); 129.3 (C); 128.1 (CH); 126.7 (CH); 125.4 (CH); 123.2 (CH); 118.5 (CH); 117.7 (C); 45.1 (CH_2_). HRMS: calcd for C_24_H_21_N_2_ [M + H]^+^: 337.1705; found: 337.1696.

***N*,*N*′-([1,1′-Binaphthalene]-2,2′-diyl)dimethanesulfonamide** (**3a**)**:** To a solution of **2** (142 mg, 0.5 mmol) in pyridine (1 mL)/DCM (4 mL) was added mesylchloride (126 mg, 1.1 mmol) and the orange mixture was stirred at r.t. After 24 h, a second portion of mesylchloride was added (126 mg, 1.1 mmol) and stirring was continued. After complete conversion (TLC), the reaction was acidified (HCl, 1 M) and sufficiently extracted with DCM. The organic phase was dried (MgSO_4_) and the solvent removed under reduced pressure. The crude mixture was purified by MPLC (EtOAc (30→50%)/heptane) to yield 223 mg (quant.) of **3a** as a mixture of tautomers; m.p.: 221–222 °C. ^1^H-NMR (*C_2_*-symmetric tautomer) δ = 8.10 (d, *J* = 8.9 Hz, 2H); 8.02 (d, *J* = 8.9 Hz, 2H); 7.95 (br.d, *J* = 8.2 Hz, 2H); 7.47 (ddd, *J* = 8.0, 6.8, 1.1 Hz, 2H); 7.31 (ddd, *J* = 8.4, 6.9, 1.3 Hz, 2H); 6.99 (br.d, *J* = 8.3 Hz, 2H); 6.02 (br.s, 2H); 2.97 (s, 6H). ^13^C-NMR δ = 134.4 (C); 132.5 (C); 131.5 (CH); 131.2 (C); 128.7 (CH); 128.2 (CH); 126.1 (CH); 124.5 (CH); 118.5 (C); 118.2 (CH); 41.0 (CH_3_). HRMS: calcd for C_22_H_20_NaN_2_O_4_S_2_ [M + Na]^+^: 463.0762; found 463.0762.

***N*,*N*′-([1,1′-Binaphthalene]-2,2′-diyl)bis(4-methylbenzenesulfonamide)** (**3b**): A similar procedure as given for **3a** was applied, yielding 252 mg (85%, 0.5 mmol scale) of **3b** as off-white solid. NMR spectra are in agreement with references [28,39].

***N*,*N*′-([1,1′-Binaphthalene]-2,2′-diyl)bis(2,2,2-trifluoroacetamide)** (**3c**)**:** To a solution of 1,1′-binaphthyl-2,2′-diamine **2** (569 mg, 2 mmol) in THF (30 mL) was added solid Na_2_CO_3_ (212 mg, 2 mmol) followed by dropwise addition of TFAA (1.27 mL, 9 mmol) in THF (30 mL). After 2 h the reaction was quenched with sat. NaHCO_3_ solution and extracted with EtOAc, washed with H_2_O and brine, dried (Na_2_SO_4_), and evaporated to give 826 mg (87%) of **3c**; colorless crystals; m.p.: 195–196 °C. The product was pure enough for the next step. ^1^H-NMR: δ = 8.19 (d, *J* = 8.9 Hz, 2H); 8.14 (d, *J* = 8.9 Hz, 2H); 8.01 (d, *J* = 8.2 Hz, 2H); 7.72 (s, 2H); 7.56 (ddd, *J* = 8.2, 6.8, 1.2 Hz, 2H); 7.38 (ddd, *J* = 8.4, 6.8, 1.3 Hz, 2H); 7.13 (dm, *J* = 8.6 Hz, 2H). ^13^C-NMR δ = 132.3 (C); 131.8 (C); 131.7 (C); 130.9 (CH); 128.7 (CH); 128.2 (CH); 127.0 (CH); 124.7 (CH); 124.0 (C); 121.8 (CH); 115.3 (CF_3_, *J*_CF_ ~280 Hz). HRMS: calcd for C_24_H_15_F_6_N_2_O_2_ [M + H]^+^: 477.1038; found 477.1041.

***N*,*N*′-([1,1′-Binaphthalene]-2,2′-diyl)bis(*N*-allylmethanesulfonamide)** (**4a**): Bis(*N*-mesylate) **3a** (220 mg, 0.5 mmol) was suspended in acetonitrile and degassed. To this was added allylbromide (420 mg, 3.5 mmol) and K_2_CO_3_ (350 mg, 2.5 mmol), and the mixture was stirred at 85 °C for 48 h. Extractive work-up with EtOAc/water left crude diallylated product which was purified by column chromatography (EtOAc (20→30%)/heptane) to yield 208 mg (80%) of **4a**; m.p.: 197–199 °C. ^1^H-NMR: δ = 7.99 (d, *J* = 8.8 Hz, 2H); 7.92 (br.d, *J* = 8.2 Hz, 2H); 7.65 (d, *J* = 8.9 Hz, 2H); 7.49 (ddd, *J* = 8.0, 6.8, 1.0 Hz, 2H); 7.26 (ddd, *J* = 8.4, 6.9, 1.3 Hz, 2H); 7.08 (d, *J* = 8.4 Hz, 2H); 5.67 (br.s, 2H); 4.96–5.08 (m, 4H); 3.73–4.05 (br.m, 4H); 2.54 (br.s, 6H). ^13^C-NMR δ = 138.1 (br.C); 133.8 (C); 132.6 (C); 132.3 (C); 129.2 (CH); 128.2 (br.CH); 127.9 (CH); 127.7 (br.CH); 126.7 (CH); 126.6 (CH); 119.3 (CH_2_); 54.4 (br.CH_2_); 41.7 (br.CH_3_). HRMS: calcd for C_28_H_28_NaN_2_O_4_S_2_ [M + Na]^+^: 543.1388; found 543.1394.

***N*,*N*′-([1,1′-Binaphthalene]-2,2′-diyl)bis(*N*-allyl-4-methylbenzenesulfonamide)** (**4b**): A similar procedure as given for the synthesis of **4a** was applied using an excess of allylbromide (8 equ.) and 48 h reflux to afford 92 mg (28%, 0.5 mmol scale) of **4b** (along with 92 mg, 29% of mono-allylated product); m.p.: 125–128 °C. ^1^H-NMR: δ = 7.94 (d, *J* = 8.8 Hz, 2H); 7.87 (d, *J* = 8.0 Hz, 2H); 7.61 (br.d, *J* = 8.8 Hz, 2H); 7.43 (ddd, *J* = 8.0, 6.9, 1.1 Hz, 2H); 7.14–7.29 (br.m, 4H); 7.18 (ddd, *J* = 8.2, 6.7, 1.1 Hz, 2H); 7.06 (br.d, *J* = 8.4 Hz, 2H); 6.96–7.08 (br.m, 4H); 5.68 (br.s, 2H); 4.76–4.91 (br.m, 4H); 4.03–4.21 (br.m, 2H); 3.73–3.93 (br.m, 2H); 2.35 (s, 6H). ^13^C-NMR δ = 143.0 (br.C); 137.3 (br.C); 134.4 (C); 134.0 (C); 133.6 (br.CH); 132.6 (C); 129.1 (CH); 128.8 (CH); 128.7 (br.CH); 127.8 (CH); 127.5 (br.CH); 126.6 (CH); 126.2 (CH); 118.7 (CH_2_); 21.5 (CH_3_). HRMS: calcd for C_40_H_36_NaN_2_O_4_S_2_ [M + Na]^+^: 695.2014; found 695.2026.

***N*,*N*′-([1,1′-binaphthalene]-2,2′-diyl)bis(*N*-allyl-2,2,2-trifluoroacetamide)** (**4c**): (*Method A*) Bis(trifluoroacetamide) **3c** (238 mg, 0.5 mmol) was dissolved in MeCN (10 mL) and degassed. To this was added K_2_CO_3_ (346 mg, 2.5 mM) and allylbromide (423 mg, 303 µL, 3.5 mM) and the mixture was stirred at reflux for 20 h. The reaction was worked up with DCM (50 mL)/water (20 mL). The organic phase was washed with water and brine and dried (MgSO_4_). After removal of solvents the crude material was subjected to MPLC (EtOAc (5→20%)/heptane) afforded 109 mg (95% purity, 40% yield) of **4c** as mixture of rotamers. Due to complexity of the ^1^H and ^13^C-NMR spectra, no signal assignment was possible (see Supplementary Materials). ^19^F-NMR: δ = −66.43 (s); −66.53 (q, *J* = 6.0 Hz); −68.43 (q, *J* = 6.0 Hz); −68.65 (s). HRMS: calcd for C_30_H_22_NaF_6_N_2_O_2_ [M + Na]^+^: 579.1483; found 579.1467.

(Method B) To a solution of diallyldiamine **6** (142 mg, 0.5 mmol), Et_3_N (101 mg, 139 µL, 1 mmol) and DIMAP (122 mg, 1 mmol) in DCM (5 mL) was added trifluoroacetic anhydride (420 mg, 282 µL, 2 mmol) at r.t. and the solution was stirred for 24 h. Extractive work-up with DCM (30 mL)/water (20 mL) and MPLC (see above) afforded of **4c** (140 mg, 50%, colorless crystals, m.p.: 175–176 °C).

***Di-tert-butyl* [1,1′-binaphthalene]-2,2′-diylbis(allylcarbamate)** (**4d**): A stirred suspension of Boc protected 2,2′-diamino-1,1′-binaphthyl **3d** [29] (242 mg, 0.5 mmol) in DMF (5 mL) was mixed at 0 °C with NaH (60 mg, 1.5 mmol, 60% in mineral oil) and then warmed up to r.t. during 30 min. The mixture was cooled to 0 °C again and treated with allylbromide (173 µL, 2 mmol) After stirring for 16 h at r.t. the reaction was diluted with EtOAc, washed with water and brine, dried (MgSO_4_), and concentrated under reduced pressure. Purification by MPLC (EtOAc (10→30%)/heptane) afforded 270 mg (93% purity, 89% yield, colorless foam) of **4d** as mixture of rotamers. ^1^H-NMR δ = 7.88 (d, *J* = 7.9 Hz, ~2H); 7.87 (d, *J* = 8.5 Hz, ~2H); 7.42 (ps.t, *J* = 7.6 Hz, ~2H); 7.33 (d, *J* = 8.9 Hz, ~1H); 7.17 (ps.t, *J* = 7.5 Hz, ~1H); 6.85 (d, *J* = 8.5 Hz, ~1H); 5.59–5.72 (m, ~1H); 4.79 (d, *J* = 10.1 Hz, ~1H); 4.54 (d, *J* = 17.1 Hz, ~1H); 3.99 (dd, *J* = 15.4, 4.0 Hz, ~1H); 2.91 (dd, *J* = 15.4, 7.8 Hz, ~1H); 1.44 (br.s, >9H). In addition several unresolved multiplets were observed between 2.8 and 8.0 ppm. HRMS: calcd for C_36_H_40_N_2_NaO_4_ [M + Na]^+^: 587.2886; found: 587.2893.

Repetition of allylation of **3d** in THF with a reaction time of 2 h at r.t. afforded 112 mg (71%, 0.3 mmol scale) of mono-allylated product, *tert*-butyl allyl(2′-((*tert*-butoxycarbonyl)amino)-[1,1′-binaphthalen]-2-yl)carbamate. ^1^H-NMR δ = 6.57–8.30 (several br.m, ~12H); 5.40–5.90 (m, 1H); 4.45–4.92 (m, 2H); 2.80–4.20 (m, 2H); 1.37; 1.28; 1.25 (3× br.s, ~18H). HRMS: calcd for C_33_H_36_N_2_NaO_4_ [M + Na]^+^: 547.2573; found: 547.2576.

**(*Z*)- and (*E*)-11,16-Bis(methylsulfonyl)-11,12,15,16-tetrahydrodinaphtho[2,1-b:1′,2′-d][1,6]diazecine, (*cis*- and *trans*-5a) (Typical procedure):** To a solution of 4a (52 mg, 0.1 mmol) in DCM (7 mL) was added at 40 °C Grubbs II catalyst (8.5 mg, 10 mol%) in DCM (3 mL) during 6 h by syringe pump. After 24 h the solvent was removed and the product mixture separated by chromatography (30→50% EtOAc/PE) to yield *trans*-5a (2 mg, 4%), *cis*-5a (35 mg, 71%), and a side product with shifted double bond *cis*-5a′ (9 mg, 18%). Repetition with Grubbs I catalysts afforded *trans*-5a (15%), *cis*-5a (55%), and 4a (2%).

***trans***-**5a**: Colorless crystals, m.p.: 248–255 °C, dec. ^1^H-NMR δ = 8.06 (d, *J* = 8.7 Hz, 2H); 7.92 (br.d, *J* = 8.2 Hz, 2H); 7.63 (br.d, *J* = 8.6 Hz, 2H), 7.62 (d, *J* = 8.6 Hz, 2H); 7.53 (ddd, *J* = 8.1, 6.9, 1.3 Hz, 2H); 7.38 (ddd, *J* = 8.3, 6.8, 1.3 Hz, 2H); 4.78–4.88 (m, 2H); 3.96–4.04 (m, 2H); 3.54–3.65 (m, 2H); 2.04 (s, 6H). ^13^C-NMR δ = 137.5 (C); 135.1 (C); 133.9 (C); 132.7 (C); 130.1 (CH); 129.5 (CH);129.1 (CH); 128.5 (CH); 127.5 (CH); 127.1 (CH); 126.5 (CH); 53.8 (CH_2_); 40.2 (CH_3_). HRMS (EI) calcd for C_26_H_24_N_2_O_4_S_2_ [M]^+^: 492.1178; found: 492.1171.

***cis***-**5a**: Colorless crystals, m.p.: 125–129 °C. ^1^H-NMR δ = 8.00 (d, *J* = 8.8 Hz, 2H); 7.90 (d, *J* = 8.2 Hz); 7.55 (d, *J* = 8.7 Hz, 2H); 7.47–7.53 (m, 2H); 7.29–7.35 (m, 4H); 5.80–5.88 (m, 2H); 4.16–4.23 (m, 4H); 1.88 (s, 6H). ^13^C-NMR δ = 137.8 (C); 135.0 (C); 133.5 (C); 132.4 (C); 129.9 (CH); 129.7 (CH); 128.4 (CH); 128.2 (CH); 127.7 (CH); 127.0 (CH); 126.7 (CH); 46.8 (CH_2_); 40.7 (CH_3_). HRMS (EI) calcd for C_26_H_24_N_2_O_4_S_2_ [M]^+^: 492.1178; found: 492.1168.

**(*Z*)- and (*E*)-11,16-Ditosyl-11,12,15,16-tetrahydrodinaphtho[2,1-b:1′,2′-d]**[1,6]**diazecine** (***cis*- and** ***trans*-5b**)**:** A similar procedure as given for **5a** was applied yielding a mixture of *cis*- and *trans*-**5b**, which was only in part separable affording *trans*-**5b** (~16%, enriched sample) and *cis*-**5b** (36 mg, 56%, 0.1 mmol scale) as a colorless foam.

*trans*-**5b**: ^1^H-NMR δ = 8.13 (d, *J* = 8.8 Hz, 2H); 8.04 (d, *J* = 8.1 Hz, 2H); 7.88 (d, *J* = 8.8 Hz, 2H); 7.81 (d, *J* = 8.4 Hz, 2H); 7.58 (ddd, *J* = 8.1, 6.8, 1.1 Hz, 2H); 7.39 (ddd, *J* = 8.3, 6.8, 1.3 Hz, 2H); 6.89 (d, *J* = 7.8 Hz, 4H); 6.70 (d, *J* = 8.2 Hz, 4H); 4.53–4.63 (m, 2H); 3.57–3.64 (m, 2H); 3.33–3.44 (m, 2H); 2.29 (s, 6H).

*cis*-**5b**: ^1^H-NMR δ = 7.98 (d, *J* = 8.8 Hz, 2H); 7.93 (br.d, *J* = 8.2 Hz, 2H); 7.50 (ddd, *J* = 8.1, 6.8, 1.1 Hz, 2H); 7.43 (d, *J* = 8.8 Hz, 2H); 7.38 (br.d, *J* = 8.4 Hz, 2H); 7.28 (ddd, *J* = 8.1, 6.7, 1.2 Hz, 2H); 6.94 (br.s, 8H); 5.42 (m, 2H); 3.92 (m, 4H); 2.31 (s, 6H). ^13^C-NMR δ = 143.5 (C); 137.4 (C); 135.9 (C); 135.6 (C); 134.1 (C); 132.6 (C); 129.41 (CH); 129.38 (CH); 129.3 (CH); 129.1 (CH); 128.1 (CH); 127.3 (CH); 126.7 (CH); 126.2 (2×CH), 47.2 (CH_2_); 21.4 (CH_3_). HRMS calcd for C_38_H_33_N_2_O_4_S_2_ [M + H]^+^: 645.1882; found: 645.1887.

***Di-tert-butyl*(*E*)-12,15-dihydrodinaphtho[2,1-b:1′,2′-d]**[1,6]**diazecine-11,16-dicarboxylate** (***trans*-5d**): To a suspension of **3d** (484 mg, 1 mmol) in DMF (10 mL) was added NaH (120 mg, 3 mmol, 60% in mineral oil) at 0 °C with stirring and after gas evolution ceased stirring was continued at r.t. for 30 min. The turbid mixture was again cooled to 0 °C, solid *trans*-1,4-dibromobut-2-ene (214 mg, 1 mmol) was added and reaction stirred at r.t. for 20 h. The mixture was diluted with EtOAc, washed with water and brine, dried and concentrated under reduced pressure. The crude product was purified by column chromatography (EtOAc(10→30%)/heptane) to give 399 mg (74%) of *trans*-**5d** as an off-white solid, m.p.: 242–243 °C. ^1^H-NMR (unresolved mixture of conformers) δ = 7.99 (br.d, *J* = 8.6 Hz, 2H); 7.86 (br.d, *J* = 8.2 Hz, 2H); 7.41–7.60 (br.m, 2H); 7.45 (br.pt, *J* = 7.4 Hz); 7.18–7.34 (br.m, 2H); 7.23 (ddd, *J* = 8.4, 7.0, 1.1 Hz, 2H); 4.79–4.89 (br.m, 2H); 4.40–4.90 (br.m, 2H); 3.18–3.42 (br.m, 2H); 1.15–1.45 (br.m, 18H). HRMS calcd for C_34_H_36_N_2_Na_2_O_4_ [M + Na]^+^: 559.2573; found: 559.2567.

***Di-tert-butyl*(*Z*)-12,15-dihydrodinaphtho[2,1-b:1′,2′-d]**[1,6]**diazecine-11,16-dicarboxylate** (**cis**-**5d**): A similar procedure as given for **5a** was applied affording exclusively *cis*-**5d** in 41 mg (76%, Grubbs I or Grubbs-Hoveyda II) or 35 mg (65%, Grubbs II) yield. Experiments were performed on a 0.1 mmol scale and 10 mol% of catalyst; colorless crystals, m.p.: 230–231 °C. ^1^H-NMR (unresolved mixture of conformers) δ = 7.86–7.98 (br.m, 2H); 7.84 (br.d, *J* = 7.7 Hz, 2H); 7.37–7.46 (br.m, 2H); 7.16–7.36 (br.m, 6H); 5.62–5.73 (br.m, 2H); 3.69–4.39 (br.m, 4H); 0.95–1.38 (br.m, 18H). HRMS calcd for C_34_H_36_N_2_Na_2_O_4_ [M + Na]^+^: 559.2573; found: 559.2566.

***N^2^,N^2^*′-Diallyl-[1,1′-binaphthalene]-2,2′-diamine** (**6**): To a solution of **2** (1.421 g, 5 mmol) in benzene (5 mL) was added allylalcohol (0.850 mL, 12.5 mmol) and dried molsieve (1 g, 4 Å) and the mixture was degassed. Subsequently, Ti(*i*-OPr)_4_, (710 mg, 740 µL, 2.5 mmol), PPh_3_ (105 mg, 0.4 mmol), and Pd(OAc)_2_ (22.5 mg, 0.1 mmol) was added and the reaction was stirred under Ar at 50 °C. The conversion was monitored by TLC. After extractive work-up with DCM/water, drying (MgSO_4_), and evaporation, the crude product was purified by chromatography in EtOAc (5→20%)/heptane to afford 1.55 g (85%) of **6** as a slightly brown crystaline solid; m.p.: 95–99 °C. ^1^H-NMR δ = 7.87 (d, *J* = 9.0 Hz, 2H); 7.78 (dm, *J* = 7.7 Hz, 2H); 7.21 (d, *J* = 9.1 Hz, 2H); 7.14–7.22 (m, 4H); 6.99 (dm, *J* = 7.9 Hz, 2H); 5.77 (ddm, *J* = 17.3, 10.3 Hz, 2H); 5.12 (dm, *J* = 17.3 Hz, 2H); 5.02 (dm, *J* = 10.3 Hz, 2H); 3.92 (br.s, 2H); 3.77–3.86 (br.m, 4H). ^13^C-NMR δ = 144.2 (C); 135.7 (CH); 133.9 (C); 129.5 (CH); 128.1 (CH); 127.7 (C); 126.7 (CH); 123.9 (CH); 122.0 (CH); 115.6 (CH_2_); 114.2 (CH); 112.0 (C); 46.1 (CH_2_). HRMS calcd for C_26_H_25_N_2_ [M + H]^+^: 365.2018; found: 365.2011.

*tert-Butyl* (10S*,11S*)-11-bromo-8-oxo-11,12-dihydro-8H-7,10-methanodinaphtho[2,1-d:1′,2′-f][1]oxa[3,8]di-azacycloundecine-13(10H)-carboxylate (7) and (10S*,11S*)-11-Bromo-10,11,12,13-tetrahydro-8*H*-7,10-methanodinaphtho[2,1-d:1′,2′-f][1]oxa[3,8]diazacycloundecin-8-one (8): Diazecine *cis*-5d (54 mg, 0.1 mmol) was added to DCM (2 mL) and the solution was cooled to 0 °C. Bromine (26 mg, 0.16 mmol) dissolved in DCM (1 mL) was added dropwise. After 16 h at r.t. the pale yellow reaction was diluted with DCM (10 mL) and stirred with NaHSO_3_ solution (10%, 3 mL). The organic phase was separated, dried, and evaporated. MPLC (EtOAc(20→40%)/heptane) afforded fractions containing 7 (18 mg, 33%, colorless crystals, m.p.: 180–185 °C, dec.) and 8 (28 mg, 62%, colorless crystals, m.p.: 254–256 °C, dec.).

**7**: ^1^H-NMR δ = 8.02 (d, *J* = 8.7 Hz, 1H); 7.96 (d, *J* = 8.5 Hz, 1H); 7.88 (br.d, *J* = 8.3 Hz); 7.48 (d, *J* = 8.8 Hz, 1H); 7.43–7.48 (m, 2H); 7.42 (d, *J* = 8.7 Hz, 1H); 7.18–7.27 (m, 3H); 7.01 (dm, *J* = 8.7 Hz, 1H); 5.01 (dd, *J* = 6.4, 3.6 Hz, 1H); 4.72 (dd, *J* = 12.7, 15.0 Hz, 1H); 4.29 (dd, *J* = 9.8, 6.4 Hz, 1H); 4.18 (dd, *J* = 15.0, 3.2 Hz, 1H); 4.09 (d, *J* = 9.9 Hz, 1H); 3.79 (dps.t, *J* = 12.7, 3.4 Hz, 1H); 0.71 (s, 9H). ^13^C-NMR δ = 153.4 (C); 134.23 (C); 134.17 (C); 133.7 (C); 133.1 (C); 132.4 (C); 132.2 (C); 132.1 (C); 131.9 (C); 130.3 (CH); 130.1 (CH); 128.5 (CH); 128.0 (CH); 127.9 (CH); 127.7 (CH); 126.6 (CH); 126.5 (CH); 126.4 (CH); 126.2 (CH); 123.3 (CH); 120.9 (CH); 82.0 (CH); 81.8 (C); 52.8 (CH_2_); 50.5 (CH_2_); 49.0 (CH); 27.4 (CH_3_). HRMS: calcd for C_30_H_27_BrN_2_NaO_4_ [M + Na]^+^: 583.1031; found: 583.1024.

**8**: ^1^H-NMR (mixture of conformers) δ = 8.03 (d, *J* = 8.5 Hz, 0.4H); 8.02 (d, *J* = 8.4 Hz, 0.6H); 7.92–7.96 (m, 1.4H); 7.90 (d, *J* = 8.8 Hz, 0.6H); 7.50–7.55 (m, 1H); 7.47 (d, *J* = 8.5 Hz, 0.6H); 7.46 (d, *J* = 8.7 Hz, 0.4H); 7.16-7.30 (m, 5H); 6.99 (dm, *J* = 8.5 Hz, 0.6H); 6.85 (dm, *J* = 9.1 Hz, 0.4H); 5.10–5.14 (m, 1H); 4.47–4.56 (m, 2H); 4.27–4.36 (m, 1H); 3.81–3.89 (m, 2H); 3.42–3.51 (m, 1H). ^13^C-NMR (mixture of conformers [40]) δ = 152.4 (C); 139.4 (C); 139.0 (C); 134.14 (C); 134.07 (C); 134.05 (C); 133.9 (C); 133.7 (C); 132.9 (C); 132.6 (C); 130.5 (CH^B^); 130.4 (CH^A^); 130.2 (CH^A^); 130.1 (CH^B^); 129.98 (CH^B^); 129.95 (C); 129.6 (C); 129.5 (CH^B^); 128.9 (C); 128.3 (CH^B^); 128.22 (CH^A^); 128.21 (CH^A^); 127.43 (CH^B^); 127.42 (CH^B^); 127.36 (CH^B^); 127.2 (CH^A^); 127.1 (CH^B^); 126.8 (CH^A^,CH^B^); 126.6 (CH^A^); 125.6 (CH^A^); 123.41 (CH^A^); 123.38 (CH^B^); 123.3 (CH^A^); 117.01 (C); 116.99 (C); 115.5 (CH^B^); 114.4 (CH^A^); 79.1 (CH^A^); 79.0 (CH^B^); 50.4 (CH^A^); 50.1 (CH_2_^A^); 50.04 (CH^B^); 50.00 (CH_2_^B^); 48.4 (CH_2_^A^); 48.3 (CH_2_^B^). HRMS: calcd for C_25_H_19_BrN_2_NaO_4_ [M + Na]^+^: 481.0528; found: 481.0516.

*Di-tert-butyl* (8S*,9R*)-9-bromo-8-(bromomethyl)-9,10-dihydro-7H-dinaphtho[2,1-f:1′,2′-h][1,5] diazonine-7,11(8*H*)-dicarboxylate (9): Diazecine *trans*-5d was treated with bromine in DCM similarly as described for *cis*-5d to afford 9 (49 mg, 71%, 0.1 mmol scale) after MPLC (EtOAc(5→20%)/heptane); m.p.: 180–183 °C, dec. ^1^H-NMR (mixture of conformers, THF solvate) δ = 7.63–8.03 (m, 5.13H); 6.85–7.52 (m, 6.90H); 5.10–5.19 (m, 0.42H); 4.49–4.79 (m, 2.60H); 3.90–3.95 (m, 0.49H); 3.73–3.77 (m, 2H, THF); 3.57–3.89 (m, 4.11H); 3.48 (dd, *J* = 14.4, 8.4 Hz, 0.48H); 1.81–1.90 (m, 2H, THF); 0.70–1.12 (6× s, 18.2H). HRMS: calcd for C_34_H_36_^79^Br^81^BrN_2_NaO_4_ [M + Na]^+^: 717.0919; found: 717.0923.

**Attempted deprotection of9**: Treatment of **9** with excess of TFA in DCM (1:1) afforded bromocarbamate **10** as white solid (12 mg, 25%, 0.1 mmol scale). ^1^H-NMR δ = 8.03 (dm, *J* = 8.9 Hz, 1H); 7.91 (dm, *J* = 8.3 Hz, 1H); 7.84 (dm, *J* = 8.9 Hz, 1H); 7.78 (dm, *J* = 8.9 Hz, 1H); 7.77 (d, *J* = 8.8 Hz, 1H); 7.47 (ddd, *J* = 8.1, 6.9, 1.2 Hz, 1H); 7.23–7.27 (m, 2H); 7.14 (ddd, *J* = 8.4, 6.9, 1.4 Hz, 1H); 7.09 (d, *J* = 8.9 Hz, 1H); 7.01 (dm, *J* = 8.4 Hz, 1H); 6.84 (dm, *J* = 8.4 Hz, 1H); 4.80 (ddd, *J* = 10.9, 9.6, 8.3 Hz, 1H); 4.34 (dd, *J* = 8.8, 8.2 Hz, 1H); 4.08 (dt, *J* = 11.1, 2.1 Hz, 1H); 3.84 (dd, *J* = 9.4, 9.0 Hz, 1H); 3.53 (dd, *J* = 17.2, 2.0 Hz, 1H); 2.93 (dd, *J* = 17.1, 2.4 Hz, 1H). ^13^C-NMR δ = 156.3 (C); 142.8 (C); 134.6 (C); 133.6 (C); 133.4 (C); 132.5 (C); 130.2 (CH); 130.0 (C); 129.6 (CH); 128.6 (C); 128.0 (CH); 127.6 (CH); 127.5 (CH); 127.2 (CH); 126.6 (CH); 125.64 (CH); 125.55 (CH); 123.6 (CH); 123.5 (CH); 120.5 (CH); 114.0 (C); 69.5 (CH_2_); 60.0 (CH); 57.3 (CH); 48.0 (CH_2_). HRMS: calcd for C_25_H_19_BrN_2_NaO_2_ [M + Na]^+^: 483.0507; found: 483.0505.

*Di-tert-butyl*(1aR*,17aS*)-1a,2,17,17a-tetrahydrodinaphtho[2,1-b:1′,2′-d]oxireno[2,3-h][1,6]**diazecine- 3,16-dicarboxylate** (**11**): To a solution of *cis*-**5d** (0.1 mmol, 54 mg) in DCM (4 mL) was added *m*-CPBA in portions (120 mg, 0.7 mmol) and the mixture was kept at r.t. overnight. To destroy excess of reagent, NaHSO_3_ (10%) was added and the organic phase was washed with Na_2_CO_3_ (2 M) and dried (Na_2_SO_4_). The crude material was purified by MPLC (EtOAc(20→50%)/heptane) to afford **11** as semisolid product (35 mg, 57%) and **11′** as a by-product (6 mg, 10%). **11**: ^1^H-NMR δ = 7.77–8.07 (br.m, 4H); 7.16–7.55 (br.m, 8H); 4.03–4.71 (br.m, 2H); 2.89–3.14 (br.m, 2H); 2.45–2.89 (br.m, 2H); 1.00–1.42 (3× br.s, 18H). HRMS: calcd for C_34_H_36_N_2_NaO_5_ [M + Na]^+^: 575.2522; found: 575.2529. **11′**: m.p.: 192–8 °C (dec.). ^1^H-NMR (DMSO-*d_6_*, 353K) δ = 8.13 (d, *J* = 8.9 Hz, 1H); 8.05 (d, *J* = 8.9 Hz, 1H); 7.98 (d, *J* = 8.3 Hz, 1H); 7.94 (d, *J* = 8.3 Hz, 1H); 7.84 (d, *J* = 8.9 Hz, 1H); 7.54 (d, *J* = 8.8 Hz, 1H); 7.46 (m, 2H); 7.21 (m, 2H); 7.01 (d, *J* = 8.6 Hz, 1H); 6.97 (d, *J* = 8.5 Hz, 1H); 4.79 (br.d, *J* = 6.3 Hz, 1H); 4.54 (d, *J* = 16.3 Hz, 1H); 4.24 (br.m, 1H); 3.99 (m, 2H); 3.91 (d, *J* = 16.3 Hz, 1H); 0.84 (s, ~9H); 0.80 (s, ~9H). ^13^C-NMR (DMSO-*d*_6_, 353K) δ = 204.4 (C); 152.5 (C); 138.1 (C); 136.0 (C); 132.8 (C); 132.7 (C); 131.9 (C); 131.4 (C); 131.04 (C); 130.99 (C); 129.4 (CH); 128.8 (CH); 128.0 (CH); 127.7 (CH); 127.0 (CH); 125.23 (CH); 125.16 (CH); 125.0 (CH); 124.2 (CH); 122.7 (CH); 80.4 (C); 79.6 (C); 68.7 (CH); 58.9 (CH_2_); 52.0 (CH_2_); 26.8 (CH_3_); 26.7 (CH_3_). HRMS: calcd for C_34_H_36_N_2_NaO_6_ [M + Na]^+^: 591.2471; found. 591.2466.

*Di-tert-butyl* (1aR*,17aR*)-1a,2,17,17a-tetrahydrodinaphtho[2,1-b:1′,2′-d]oxireno[2,3-h][1,6]diazecine-3,16-dicarboxylate (12): Epoxide 12 was accessed from *trans*-5d similarly as described for 11, with the exception that 3 equ. of *m*-CPBA were used; the reaction was complete after 6 h at r.t. Crystalline colorless material was obtained by slow evaporation from DCM/heptane solution; 49 mg (88% yield, 0.1 mmol scale). ^1^H-NMR δ = 7.14–8.12 (m, ~12H); 4.43–5.18 (br.m, 2H); 2.48 (br.m, 2H); 2.08 (dm, *J* = 9.3 Hz, 2H); 1.27 (br.s, ~18H). HRMS: calcd for C_34_H_36_N_2_NaO_5_ [M + Na]^+^: 575.2522; found: 575.2526.

**Attempted deprotection of 11 and 12**: To epoxide **11** (32 mg, 0.06 mmol) in DCM (2 mL) was added TFA (19 µL). After 22 h the reaction was neutralized (NaHCO_3_) and extracted. MPLC (MeOH(0→5%)/DCM) afforded 23 mg of **13** (80–90% purity). ^1^H-NMR δ = 7.98 (d, *J* = 8.8 Hz, 1H); 7.97 (d, *J* = 8.7 Hz, 1H); 7.88 (br.d, *J* = 8.1 Hz, 1H); 7.85 (br.d, *J* = 8.1 Hz, 1H); 7.46 (d, *J* = 8.7 Hz, 1H); 7.43 (ddd, *J* = 8.1, 6.7, 1.3 Hz, 1H); 7.40 (ddd, *J* = 8.0, 6.7, 1.1 Hz, 1H); 7.32 (d, *J* = 8.7 Hz, 1H); 7.21 (ddd, *J* = 8.6, 6.6, 1.3 Hz, 1H); 7.16 (dm, *J* = 8.7 Hz, 1H); 7.10 (ddd, *J* = 8.6, 6.8, 1.4 Hz, 1H); 6.85 (br.s, 1H); 6.83 (br.d, *J* = 8.7 Hz, 1H); 4.78 (ddd, *J* = 8.8, 5.7, 1.9 Hz, 1H); 4.51 (t, *J* = 9.2 Hz, 1H); 4.43 (dd, *J* = 15.0, 1.9 Hz, 1H); 4.00–4.03 (m, 1H); 3.95 (dd, *J* = 9.3, 2.1 Hz, 1H); 3.81 (dd, *J* = 15.0, 3.6 Hz, 1H); 0.56 (s, 9H). ^13^C-NMR δ = 152.8 (C); 138.8 (C); 134.2 (C); 133.59 (C); 133.55 (C); 132.1 (C); 131.8 (C); 130.7 (C); 130.2 (CH); 129.9 (CH); 128.6 (CH); 128.3 (CH); 128.0 (CH); 127.9 (C); 127.5 (CH); 126.52 (CH); 126.45 (CH); 126.2 (CH);125.8 (CH); 123.7 (CH); 122.5 (CH); 82.8 (C); 72.7 (CH); 68.6 (CH); 56.4 (CH_2_); 49.2 (CH_2_); 27.2 (CH_3_). HRMS: calcd for C_30_H_28_N_2_NaO_5_ [M + Na]^+^: 519.1896; found: 519.1897.

**Similar treatment of epoxide 12 (TFA, DCM, r.t., 22 h) afforded a mixture of diaminoepoxide 14 and hydroxyaziridine 15**: (1aR*,17aR*)-1a,2,3,16,17,17a-Hexahydrodinaphtho[2,1-b:1′,2′-d]oxireno[2,3-h][1,6]*diazecine* (**14**): 9 mg (31% yield, 0.08 mmol scale); colorless oil. ^1^H-NMR δ = 7.96 (d, *J* = 8.8 Hz, 2H); 7.91 (br.d, *J* = 8.1 Hz, 2H); 7.41–7.45 (m, 2H); 7.42 (d, *J* = 8.6 Hz, 2H); 7.35 (ddd, *J* = 8.3, 6.7, 1.4 Hz, 2H); 7.28 (dm, *J* = 8.4 Hz, 2H); 3.56 (dd, *J* = 13.7, 3.0 Hz, 2H); 2.88 (br.s, 2H); 2.63–2.72 (br.m, 2H); 2.03–2.08 (m, 2H). ^13^C-NMR δ = 144.6 (C); 133.7 (C); 130.3 (C); 129.9 (CH); 128.3 (CH); 127.5 (CH); 125.0 (CH); 124.8 (CH); 124.6 (C); 123.7 (CH); 55.1 (CH); 52.2 (CH_2_). HRMS: calcd for C_24_H_21_N_2_O [M + H]^+^: 353.1648; found: 353.1651.

(13R*,13aS*)-12,13,13a,14-Tetrahydro-11H-azirino[1,2-a]dinaphtho[2,1-f:1′,2′-h][1,5]**diazonin-13-ol** (**15**): 9 mg (30% yield, 90% purity, 0.08 mmol scale). ^1^H-NMR δ = 7.94 (d, *J* = 8.8 Hz, 1H); 7.93 (d, *J* = 8.7 Hz, 1H); 7.90 (br.d, *J* = 8.3 Hz, 1H); 7.84 (br.d, *J* = 8.0 Hz, 1H); 7.47 (d, *J* = 8.9 Hz, 1H); 7.39 (ddd, *J* = 8.0, 5.3, 2.7 Hz, 1H); 7.25-7.27 (m, 3H); 7.25 (d, *J* = 8.8 Hz, 1H); 7.16 (ddd, *J* = 8.1, 6.7, 1.2 Hz, 1H); 6.88 (d, *J* = 8.4 Hz, 1H); 3.33–3.40 (m, 2H); 2.86 (td, *J* = 9.3, 4.5 Hz, 1H); 2.86 (br.s, ~1H); 2.34 (d, *J* = 4.6 Hz, 1H); 2.23 (d, *J* = 3.0 Hz, 1H); 2.12 (ddd, *J* = 8.8, 4.5, 3.0 Hz, 1H). ^13^C-NMR δ = 145.6 (C); 145.4 (C); 134.3 (C); 132.7 (C); 130.4 (C); 130.3 (C); 129.9 (CH); 129.3 (CH); 128.1 (CH); 128.0 (CH); 127.1 (CH); 126.7 (CH); 126.01 (C); 125.1 (CH); 124.8 (CH); 124.6 (CH); 124.0 (CH); 122.4 (CH); 120.5 (CH); 73.8 (CH); 55.2 (br.CH_2_); 44.4 (CH); 29.2 (CH_2_). HRMS: calcd for C_24_H_21_N_2_O [M + H]^+^: 353.1648; found: 353.1651.

*Di-tert-butyl*(9S*,10S*)-9,10-dihydroxy-8,9,10,11-tetrahydrodinaphtho[2,1-b:1′,2′-d][1,6]**diazecine- 7,12-dicarboxylate** (**16**): To a solution of *trans*-**5d** (54 mg, 0.1 mmol) in THF/water (10:1, 2 mL) was added 2 equ. of NMO (50% in water, 25 mg) and K_2_OsO_4_∙H_2_O (0.01 mmol, 3.7 mg). After stirring for 24 h at r.t., solid Na_2_S_2_O_3_ (17 mg) was added and stirring was continued for 1 h. The mixture was diluted with DCM (19 mL), dried (MgSO_4_), filtered, and concentrated. MPLC (EtOAc(50→100%)/heptane) afforded 48 mg (85%) of **16** as colorless solid; m.p.: 201–205 °C. ^1^H-NMR δ = 7.94 (br.d, *J* = 8.6 Hz, 2H); 7.87 (d, *J* = 7.9 Hz, 2 H); 7.45 (ddd, *J* = 8.3, 6.2, 2.0 Hz, 2H); 7.33–7.46 (br.m, 2H); 7.19–7.26 (br. m, 4H); 3.40–3.86 (br.m, 4H); 3.31 (br.s, 2H); 2.27–2.95 (br.s, 2H); 1.18 (br.s, 18H). ^13^C-NMR δ = 138.9 (br.C); 133.1 (C); 132.3 (C); 129.3 (br.CH); 127.8 (br.CH); 127.5 (br.CH); 126.2 (2CH); 126.1 (CH); 80.7 (CH_2_); 73.9 (br.CH); 28.0 (CH_3_). HRMS: calcd for C_34_H_38_N_2_NaO_6_ [M + Na]^+^: 593.2628; found: 593.2624.

(9S*,10S*)-7,8,9,10,11,12-Hexahydrodinaphtho[2,1-b:1′,2′-d][1,6]**diazecine-9,10-diol** (**17**): To a solution of diol **16** (57 mg, 0.1 mmol) in DCM (1 mL) was added TFA (1 mL) and the reaction was stirred for 2 h at r.t. The mixture was concentrated under reduced pressure and the residue was dissolved in EtOAc (10 mL). Solid Na_2_CO_3_ was added and the mixture was stirred for 30 min. After filtration and evaporation of solvent the crude material was purified by MPLC (EtOAc(50→100%)/heptane) to afforded 32 mg (86%) of **17** as colorless crystalline solid; m.p: >165 °C (dec.). ^1^H-NMR (DMSO-*d_6_*) δ = 7.82 (d, *J* = 9.1 Hz, 2H); 7.77 (dd, *J* = 8.0, 1.1 Hz, 2H); 7.49 (d, *J* = 9.2 Hz, 2H); 7.14 (ddd, *J* = 7.9, 6.6, 1.2 Hz, 2H); 7.09 (ddd, *J* = 8.4, 6.7, 1.4 Hz, 2H); 6.79 (br.d, *J* = 8.4 Hz, 2H); 5.01–5.04 (m, 2H); 4.54 (d, *J* = 11.8 Hz, 2H); 4.11 (s, 2H); 3.80 (br.dd, *J* = 14.8, 12.6 Hz, 2H); 3.31 (d, *J* = 14.9 Hz, 2H). ^13^C-NMR (DMSO-*d*_6_) δ = 145.9 (C); 133.7 (C); 128.4 (CH); 128.0 (CH); 127.3 (C); 125.8 (CH); 124.0 (CH); 121.3 (CH); 117.6 (CH); 111.9 (C); 72.9 (CH); 48.0 (CH_2_). HRMS: calcd for C_24_H_23_N_2_O_2_ [M + H]^+^: 371.1760; found: 371.1745.

*Di-tert-butyl (*9R*,10S*)-9,10-dihydroxy-8,9,10,11-tetrahydrodinaphtho[2,1-b:1′,2′-d][1,6]**diazecine- 7,12-dicarboxylate** (**18**): A procedure similarly as described for **16** was applied to give **18**; 51 mg (89% yield, colorless solid, 0.1 mmol scale); m.p.: 133–135 °C. ^1^H-NMR δ = 7.96 (d, *J* = 8.9 Hz, 1H); 7.94 (d, *J* = 8.9 Hz, 1H); 7.86 (d, *J* = 6.2 Hz, 1H); 7.84 (d, *J* = 6.2 Hz, 1H); 7.56 (br.d, *J* = 8.2 Hz, 1H); 7.39–7.45 (m, 2H); 7.29 (br.d, *J* = 8.7 Hz, 1H); 7.15–7.22 (m, 2H); 7.09–7.14 (br.m, 1H); 3.9–4.6 (br.s, ~2H); 4.04 (dd, *J* = 13.6, 4.6 Hz, 1H); 3.72–3.85 (m, 2H); 3.20–3.33 (m, 1H); 2.6–3.8 (br.s, ~2H); 1.01 (s, 9H); 0.93 (s, 9H). ^13^C-NMR δ = 140.0 (C); 137.3 (br.C); 133.7 (C); 133.5 (C); 132.5 (C); 132.0 (C); 131.8 (C); 130.1 (CH); 129.3 (CH); 128.7 (br.CH); 128.6 (br.CH); 127.5 (CH); 126.03 (CH); 125.95 (CH); 125.9 (CH); 125.7 (CH); 125.2 (br.CH); 80.8 (C); 80.7 (C); 54.5 (CH_2_); 48.3 (br.CH_2_); 27.9 (CH_3_); 27.7 (CH_3_). HRMS: calcd for C_34_H_38_N_2_NaO_6_ [M + Na]^+^: 593.2628; found: 593.2618.

(9R*,10S*)-7,8,9,10,11,12-Hexahydrodinaphtho[2,1-b:1′,2′-d][1,6]**diazecine-9,10-diol** (**19**): A procedure as described similarly for **17** was applied to give **19**; yield: 21 mg (72%, colorless solid, 0.08 mmol scale); m.p.: 240-245 °C (dec.). ^1^H-NMR (DMSO-*d*_6_) δ = 7.93 (d, *J* = 9.0 Hz, 1H); 7.83 (dm, *J* = 7.9 Hz, 1H); 7.81 (d, *J* = 9.1 Hz, 1H); 7.78 (dm, *J* = 7.9 Hz, 1H); 7.49 (d, *J* = 9.1 Hz, 1H); 7.48 (d, *J* = 9.1 Hz, 1H); 7.09–7.21 (m, 4H); 6.83 (dm, *J* = 8.4 Hz, 1H); 6.79 (dm, *J* = 8.3 Hz, 1H); 5.01 (d, *J* = 4.1 Hz, 1H); 4.79 (d, *J* = 4.4 Hz, 1H); 4.07 (dd, *J* = 12.0, 2.3 Hz, 1H); 3.91 (ddd, *J* = 14.6, 12.0, 2.6 Hz, 1H); 3.71–3.79 (m, 2H); 3.59–3.68 (m, 1H); 3.06–3.14 (m, 2H). ^13^C-NMR (DMSO-*d*_6_) δ = 146.2 (C); 144.9 (C); 134.0 (C); 133.6 (C); 129.4 (CH); 128.3 (CH); 128.2 (CH); 128.0 (CH); 127.8 (C); 127.6 (C); 126.1 (CH); 125.9 (CH); 123.9 (CH); 123.7 (CH); 121.7 (CH); 121.6 (CH); 118.5 (CH); 116.5 (CH); 113.7 (C); 112.6 (C); 79.7 (CH); 70.1 (CH); 50.1 (CH_2_); 47.7 (CH_2_). HRMS: calcd for C_24_H_23_N_2_O_2_ [M + H]^+^: 371.1760; found: 371.1754.

***Di-tert-butyl*8,9,10,11-tetrahydrodinaphtho[2,1-b:1′,2′-d]**[1,6]**diazecine-7,12-dicarboxylate** (**20**)**:** To a solution of *cis*- or *trans*-**5d** (54 mg, 0.1 mmol) in THF/water (3 + 3 mL) was added Pd/C (10%, 5 mg) and the mixture was stirred under H_2_ (2 bar) at r.t. for 2 h. After filtration and concentration, the crude product was purified by MPLC (EtOAc(25→40%)/heptane) to afforded 49 mg (92% from *cis*-**5d**), and 51 mg (94% from *trans*-**5d**) of **20**, respectively as colorless crystaline solid; m.p.: 172–173 °C. ^1^H-NMR δ = 7.93 (d, *J* = 8.7 Hz, 2H); 7.84 (br.d, *J* = 8.1 Hz, 2H); 7.33–7.44 (br.m, 4H); 7.14–7.21 (br.m, 4H); 3.84–3.93 (br.m, 2H); 3.50–3.66 (br.m, 2H); 1.78 (br.s, 2H); 1.52–1.63 (br.m, 2H); 0.99 (s, 18H). ^13^C-NMR δ = 139.0 (br.C); 133.8 (C); 132.9 (br.C); 132.1 (C); 129.3 (CH); 128.9 (br.CH); 127.2 (br.CH); 125.6 (CH); 125.5 (CH); 125.1 (br.CH); 79.9 (CH_2_); 48.9 (CH_2_); 27.9 (CH_3_). HRMS: calcd for C_34_H_39_N_2_O_4_ [M + H]^+^: 539.2910; found: 539.2909.

**7,8,9,10,11,12-Hexahydrodinaphtho[2,1-b:1′,2′-d]**[1,6]**diazecine** (**21**): To **20** (53 mg, 0.1 mmol) dissolved in DCM (1 mL) was added TFA (1 mL) and the solution was stirred at r.t. for 2 h. The solvents were removed under vacuum and the crude product was dissolved in DCM (10 mL). Solid Na_2_CO_3_ was added and the mixture was stirred for 30 min. After filtration and concentration the pure product was obtained by MPLC (EtOAc(10→80%)/heptane); yield: 27 mg (81%, colorless crystals); m.p.: 275–278 °C. ^1^H-NMR δ = 7.92 (d, *J* = 8.9 Hz, 2H); 7.82 (dm, *J* = 7.9 Hz, 2H); 7.40 (d, *J* = 8.9 Hz, 2H); 7.28 (ddd, *J* = 8.1, 6.8, 1.3 Hz, 2H); 7.19 (ddd, *J* = 8.4, 6.8, 1.4 Hz, 2H); 7.08 (dm, *J* = 8.4 Hz, 2H); 4.00 (br.d, *J* = 11.7 Hz, 2H); 3.74 (br.t, *J* = 12.7 Hz, 2H); 2.76 (br.t, *J* = 13.5 Hz, 2H); 1.69–1.78 (m, 2H); 1.32–1.40 (m, 2H). ^13^C-NMR δ = 144.3 (C); 134.5 (C); 129.8 (CH); 129.0 (C); 128.0 (CH); 126.8 (CH); 124.7 (CH); 123.1 (CH); 117.9 (CH); 117.7 (C); 46.5 (CH_2_); 25.9 (CH_2_). HRMS: calcd for C_24_H_23_N_2_ [M + H]^+^: 339.1861; found: 339.1855.

### 3.3. X-ray Structure Analysis

Suitable crystals were obtained by slow evaporation from solvent mixtures at r.t; DCM/heptane was used in for **11′** (Supplementary Materials), **12**, and **21** (Supplementary Materials), all other compounds crystallized from ethyl acetate/heptane. Details of X-ray structure analysis can be found in Table 1 and Table 2. Solid state biaryl angles are summarized in Table 3.

## Figures and Tables

**Table 1:** Crystal structure data of *cis*- and *trans*-**1**, **4a**, **4c**, and *cis*- and *trans*-**5a.**

	*cis*-1	*trans*-1	4a	4c	*cis*-5a	*trans*-5a
M [g/mol]	336.42	336.42	520.64	556.49	614.98	985.18
Space group	P2_1_/n	P2_1_2_1_2_1_	P2_1_2_1_2_1_	C2/c	P-1	P2_1_/n
a [Å]	14.133(5)	10.5405(4)	9.7339(11)	13.8966(4)	10.7270(5)	10.8879(4)
b [Å]	11.145(3)	11.5662(5)	11.2343(11)	13.3655(4)	11.3356(5)	13.6381(5)
c [Å]	22.787(6)	14.4010(5)	22.750(3)	13.9480(4)	12.4265(6)	15.6689(5)
α[°]	90	90	90	90	86.543(2)	90
β[°]	105.704(13)	90	90	103.583(2)	81.059(3)	90.396(2)
γ[°]	90	90	90	90	71.569(2)	90
V [Å^3^]	3455.2(17)	1755.68(12)	2487.8(5)	2518.17(13)	1416.00(12)	2326.62(14)
Z	8	4	4	4	2	2
D_calc_ [g/cm^3^]	1.293	1.273	1.39	1.468	1.442	1.406
R_int_	0.1641	0.0405	0.0988	0.0569	0.0303	0.0738
R_sigma_	0.2728	0.0255	0.0577	0.0272	0.0152	0.0424
R1 (I ≥ 2σ (I))	0.0772	0.0333	0.0348	0.0439	0.0482	0.0406
wR2 (all data)	0.2084	0.0848	0.0829	0.1186	0.154	0.1039

**Table 2:** Crystal structure data of *cis*- and *trans*-**5d**, **7**, **9**, and **12**.

	*cis*-5d	*trans*-5d	7	9	12
M [g/mol]	538.66	536.65	559.44	732.52	550.65
Space group	C2/c	C2/c	P2_1_/n	C2/c	C2/c
a [Å]	19.795(4)	19.7641(15)	9.4794(6)	23.929(2)	19.4742(11)
b [Å]	12.914(4)	12.6467(8)	21.6529(14)	12.375(2)	13.0437(11)
c [Å]	13.521(4)	14.2008(9)	12.2608(6)	24.078(3)	14.0412(12)
α[°]	90	90	90	90	90
β[°]	124.816(10)	127.156(3)	92.249(2)	111.178(4)	125.810(3)
γ[°]	90	90	90	90	90
V [Å^3^]	2837.5(12)	2828.9(3)	2514.7(3)	6648.4(15)	2892.5(4)
Z	4	4	4	8	4
D_calc_ [g/cm^3^]	1.261	1.26	1.478	1.464	1.264
R_int_	0.0515	0.0713	0.0683	0.0745	0.0549
R_sigma_	0.0585	0.0879	0.0555	0.1078	0.0286
R1 (I ≥ 2σ (I))	0.0604	0.0583	0.0411	0.0483	0.0374
wR2 (all data)	0.1535	0.1557	0.0997	0.0975	0.0977

**Table 3:** Biaryl angles in crystal structures.

	*cis*-1	*trans*-1	4a	4c	*cis*-5a	*trans*- 5a	*cis*-5d	*trans*-5d	7	9	11	12	21
biaryl angle/° ^1^	68.0/67.4 ^2^	98.9	72.2	77.8	96.3	97.3	95.8	101.1	66.5	70.0	68.0	99.9	73.2

^1^ Defined as angle between binaphthyl planes, values rounded to one digit after decimal point. ^2^ Two molecules in the asymmetric unit.

## Supplementary Materials

The following are available online, containing ^1^H- and ^13^C-NMR charts and details of crystal structure determinations.
