# Supplementary material for: Synthesis, Structure, and Reactivity of Binaphthyl Supported Dihydro[1,6]diazecines
Source: Molecules. 2019 Aug 26;24(17):3098. doi: 10.3390/molecules24173098 (PMC6749229; doi:10.3390/molecules24173098)
Supplement: Supplementary file 1 [file molecules-24-03098-s001.pdf]

Supporting Information

Synthesis, structure and reactivity of binaphthyl supported dihydro[1,6]diacecines

Miran Lemmerer,[a] Michael Abraham,[b] Bogdan R. Brutiu,[a] Alexander Roller,[c] and Michael Widhalm[b]*

[a] Institute of Organic Chemistry, University of Vienna, Währinger Straße 38, 1090 Wien, Austria.

[b] Institute of Chemical Catalysis, University of Vienna, Währinger Straße 38, 1090 Wien, Austria.

[c] Institute of Inorganic Chemistry, University of Vienna, Währinger Straße 42, 1090 Wien, Austria.

Contents

^{1}H and ^{13}C NMR spectra..1-57

X-ray structure analyses..58-86

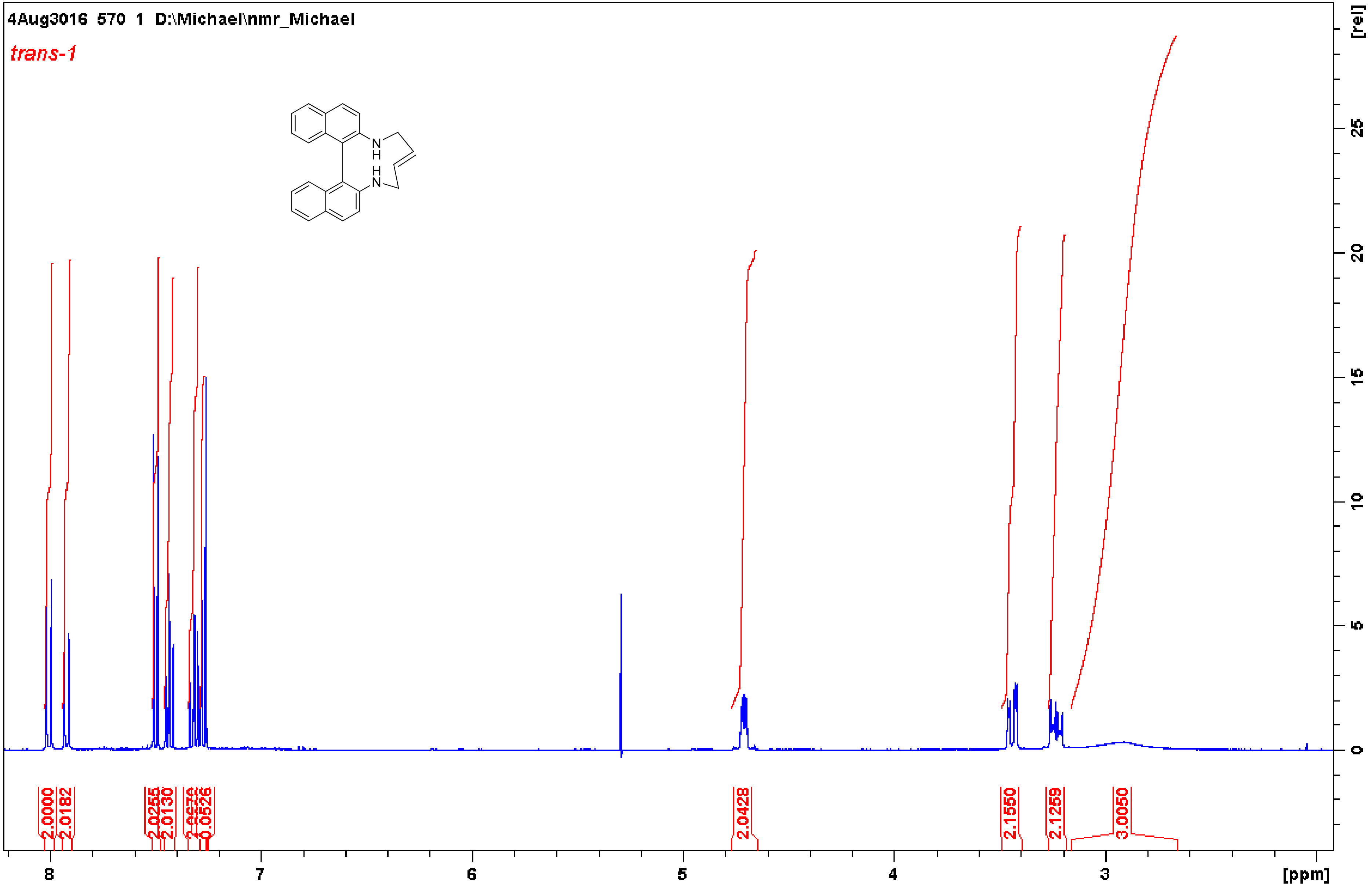
4Aug3016 570 1 D:\Michael\nmr_Michael
trans-1
N
H
H
N
2.0000
2.0182
2.0255
2.0130
2.0428
2.1550
2.1259
3.0050
8
7
6
5
4
3
[ppm]
[rel]
0
5
10
15
20
25

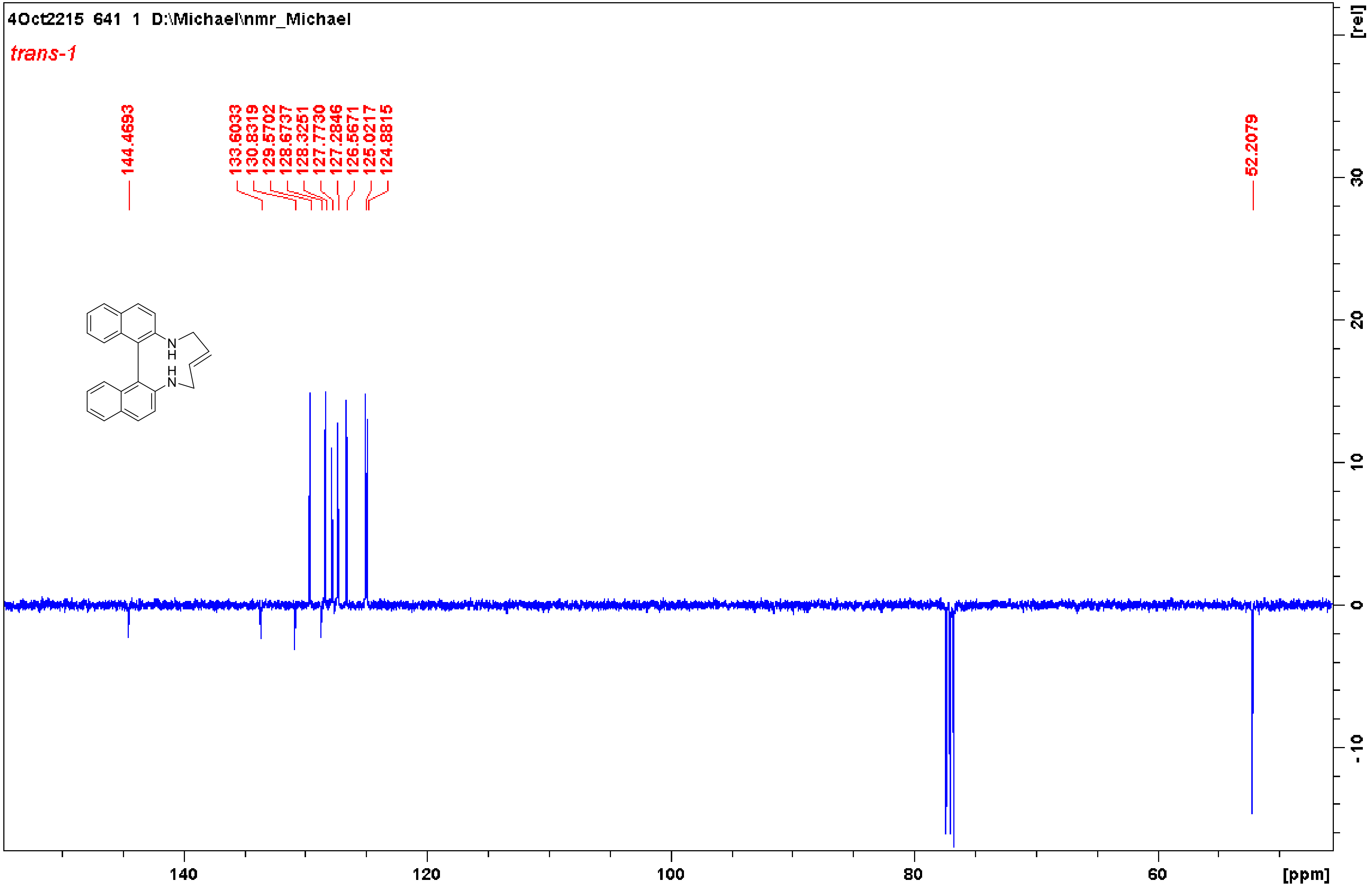

4Oct2215 641 1 D:\Michael\nmr_Michael
trans-1
144.4693
133.6033
130.8319
129.5702
128.6737
128.3251
127.7730
127.2846
126.5671
125.0217
124.8815
52.2079
N
H
H
N
[rel]
30
20
10
0
-10
140
120
100
80
60
[ppm]

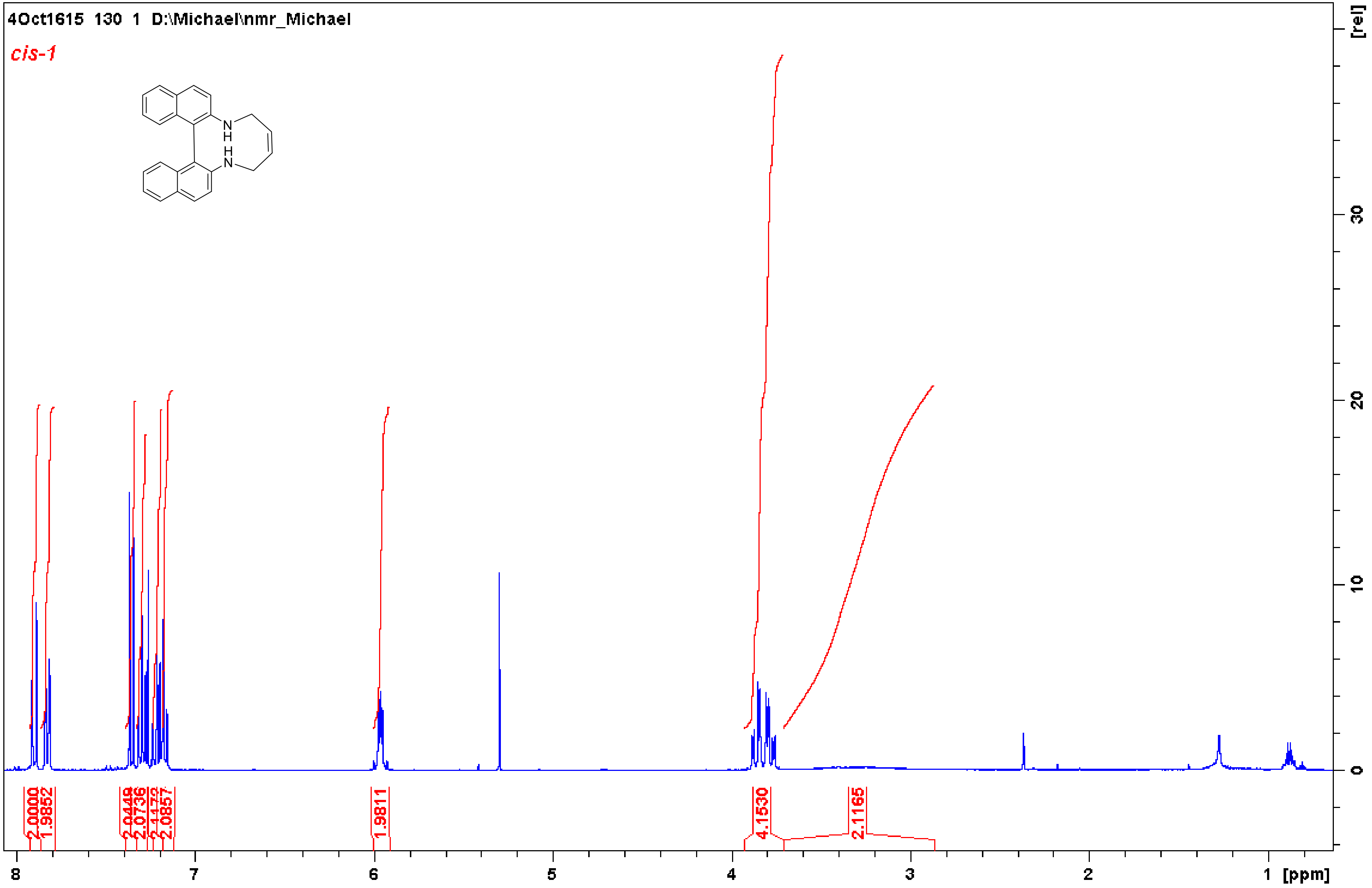
4Oct1615 130 1 D:\Michael\nmr_Michael
cis-1
[rel]
30
20
10
0
2.0000
1.9852
2.0449
2.0736
2.1172
2.0857
1.9811
4.1530
2.1165
8
7
6
5
4
3
2
1 [ppm]

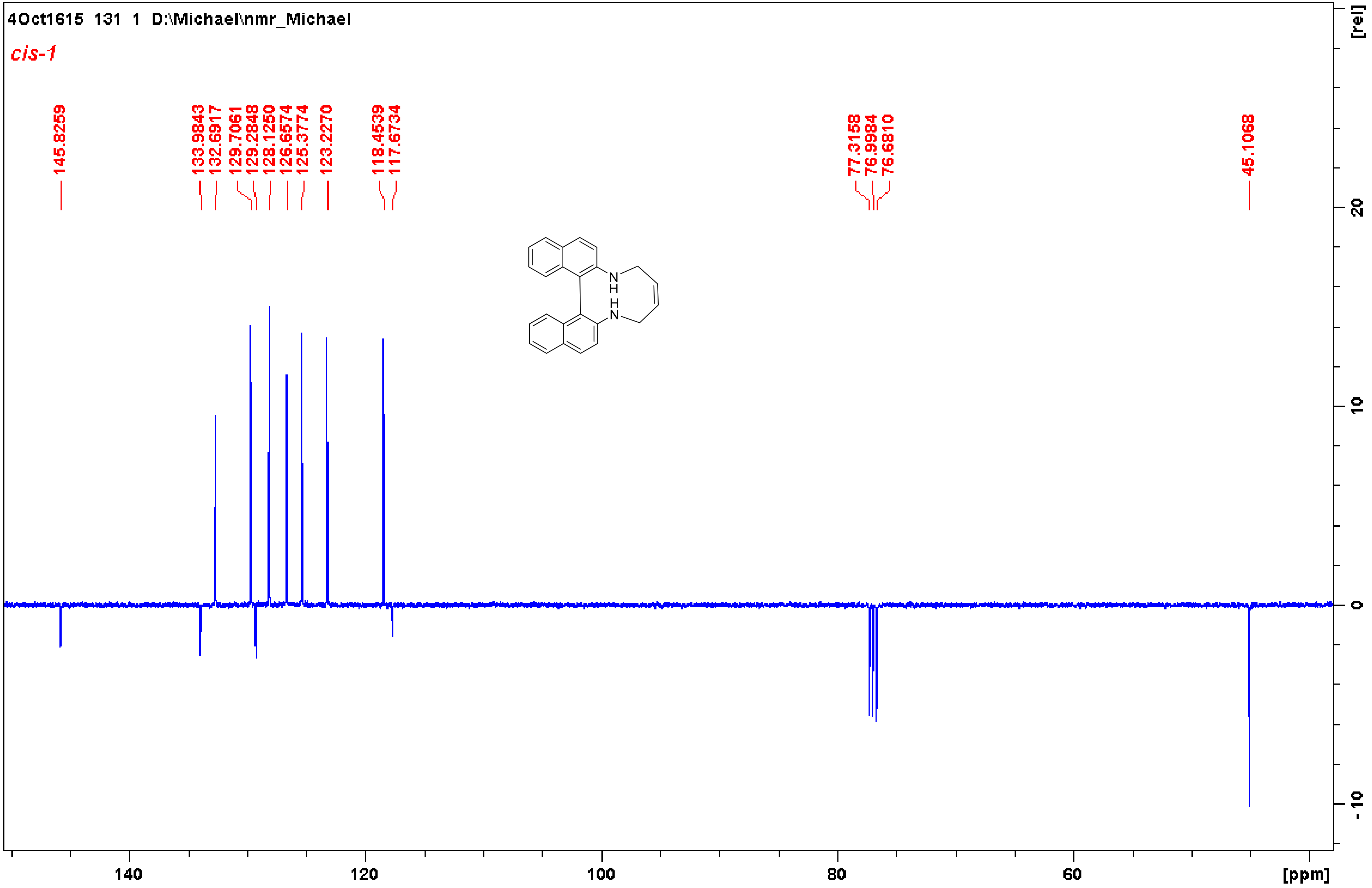
4Oct1615 131 1 D:\Michael\nmr_Michael
cis-1
145.8259
133.9843
132.6917
129.7061
129.2848
128.1250
126.6574
125.3774
123.2270
118.4539
117.6734
77.3158
76.9984
76.6810
45.1068
N
H
H
N
[rel]
20
10
0
-10
140
120
100
80
60
[ppm]

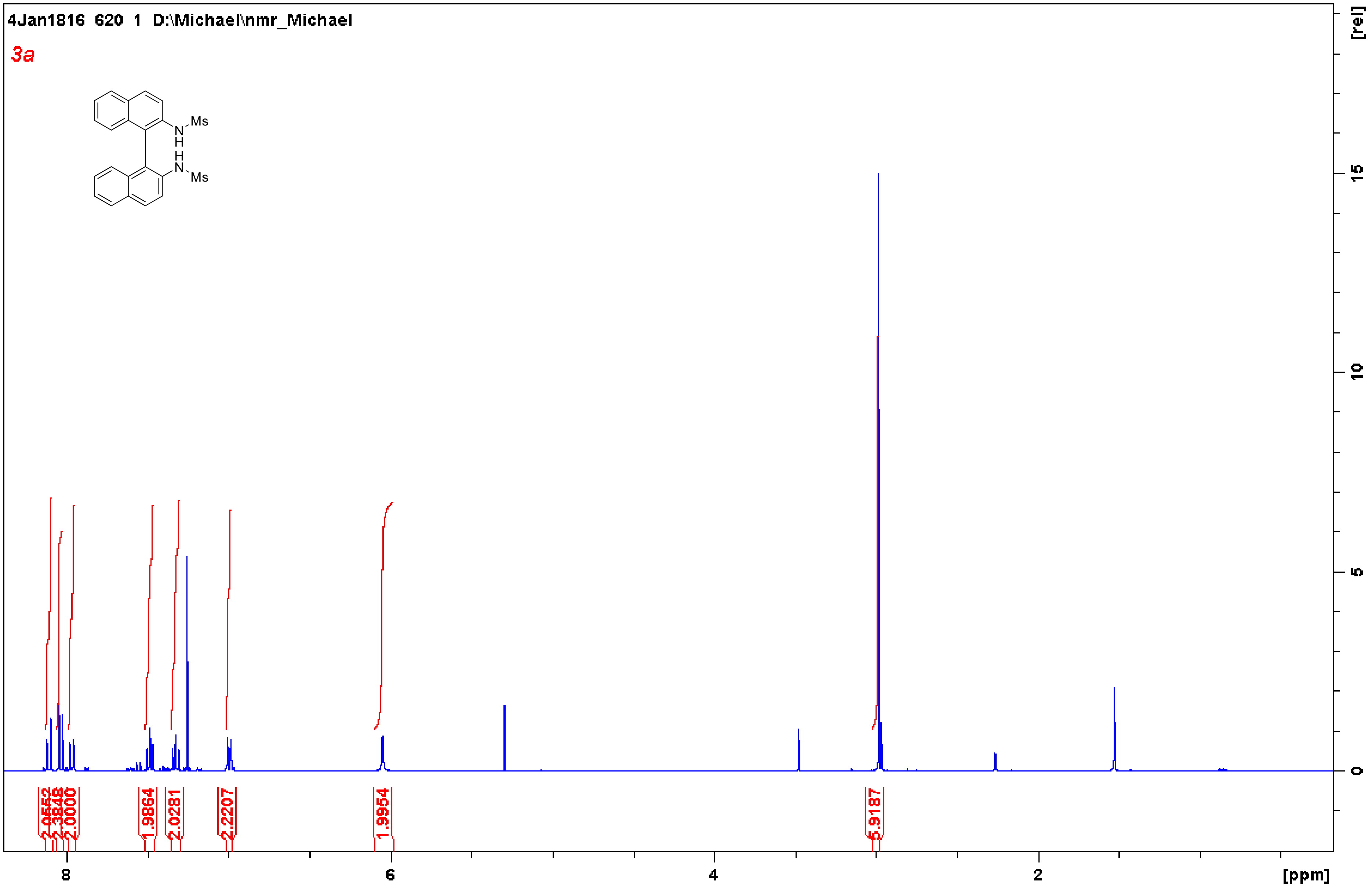
4Jan1816 620 1 D:\Michael\nmr_Michael
3a
N
H
Ms
H
N
Ms
2.0552
2.3848
2.0000
1.9864
2.0281
2.2207
1.9954
5.9187
8
6
4
2
[ppm]
0
5
10
15
[rel]

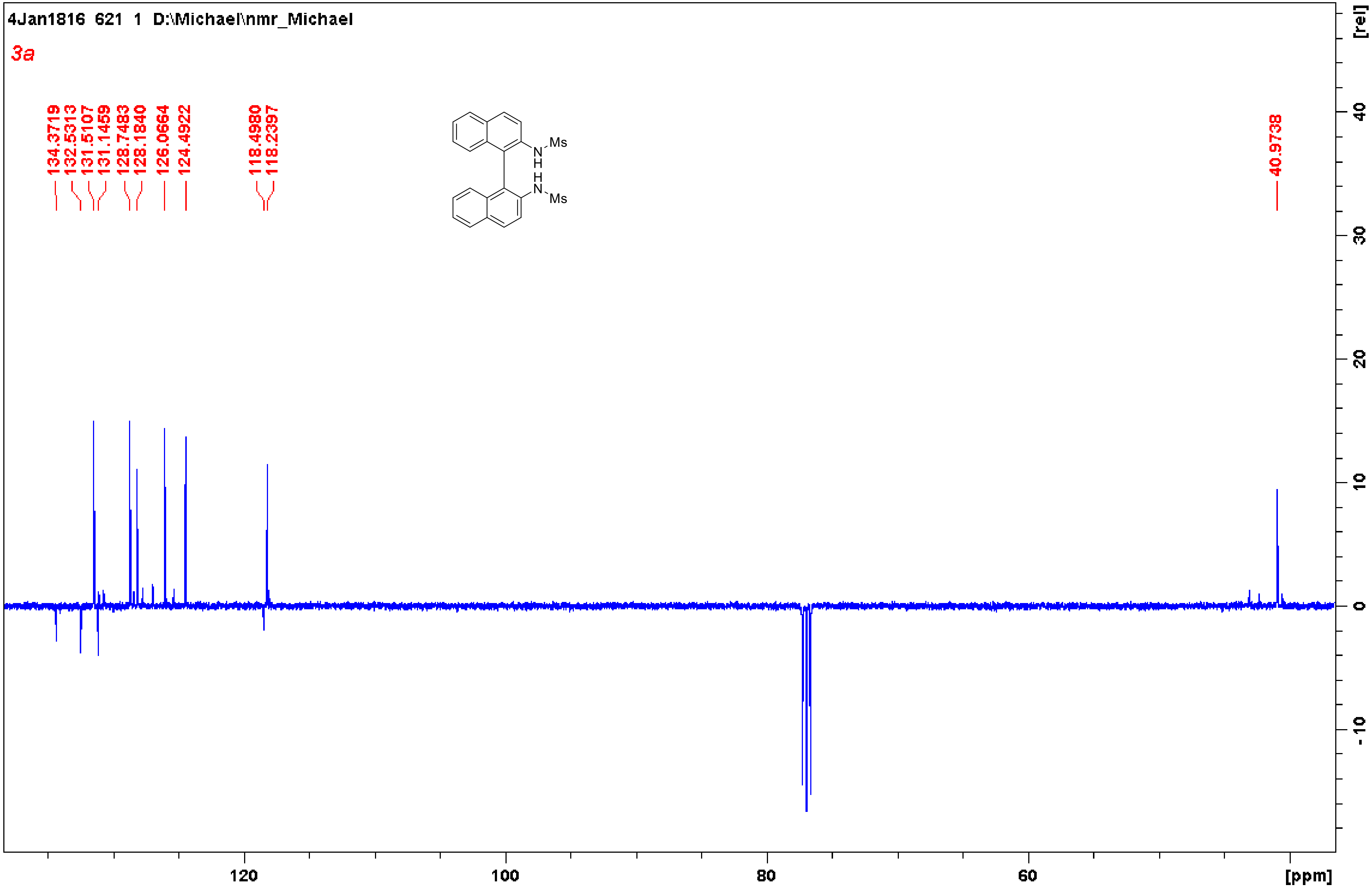
4Jan1816 621 1 D:\Michael\nmr_Michael
3a
134.3719
132.5313
131.5107
131.1459
128.7483
128.1840
126.0664
124.4922
118.4980
118.2397
40.9738
N
H
Ms
H
N
Ms
[rel]
40
30
20
10
0
-10
120
100
80
60
[ppm]

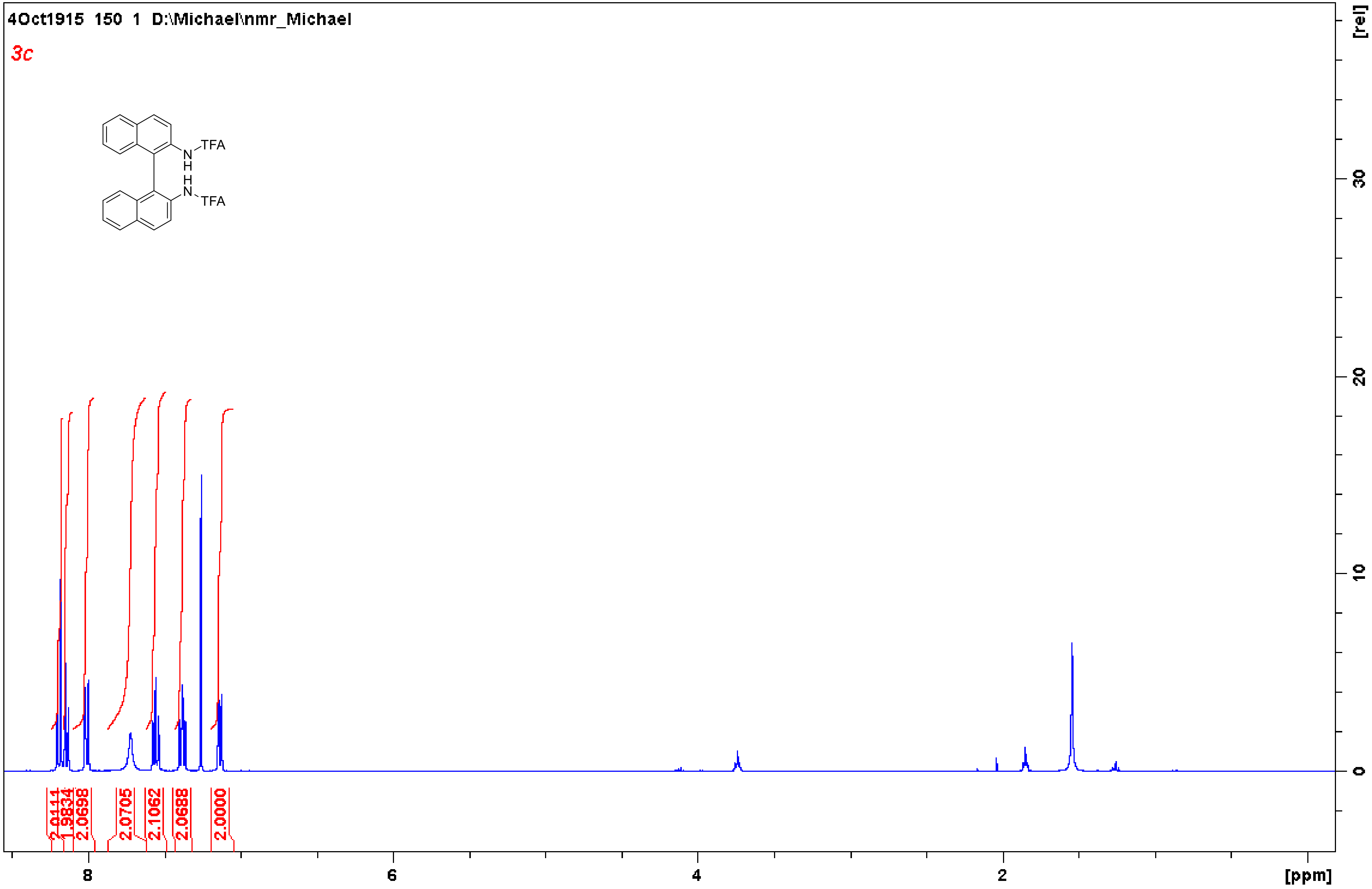

4Oct1915 150 1 D:\Michael\nmr_Michael
3c
TFA
TFA
2.0111
1.9834
2.0698
2.0705
2.1062
2.0688
2.0000
[rel]
[ppm]

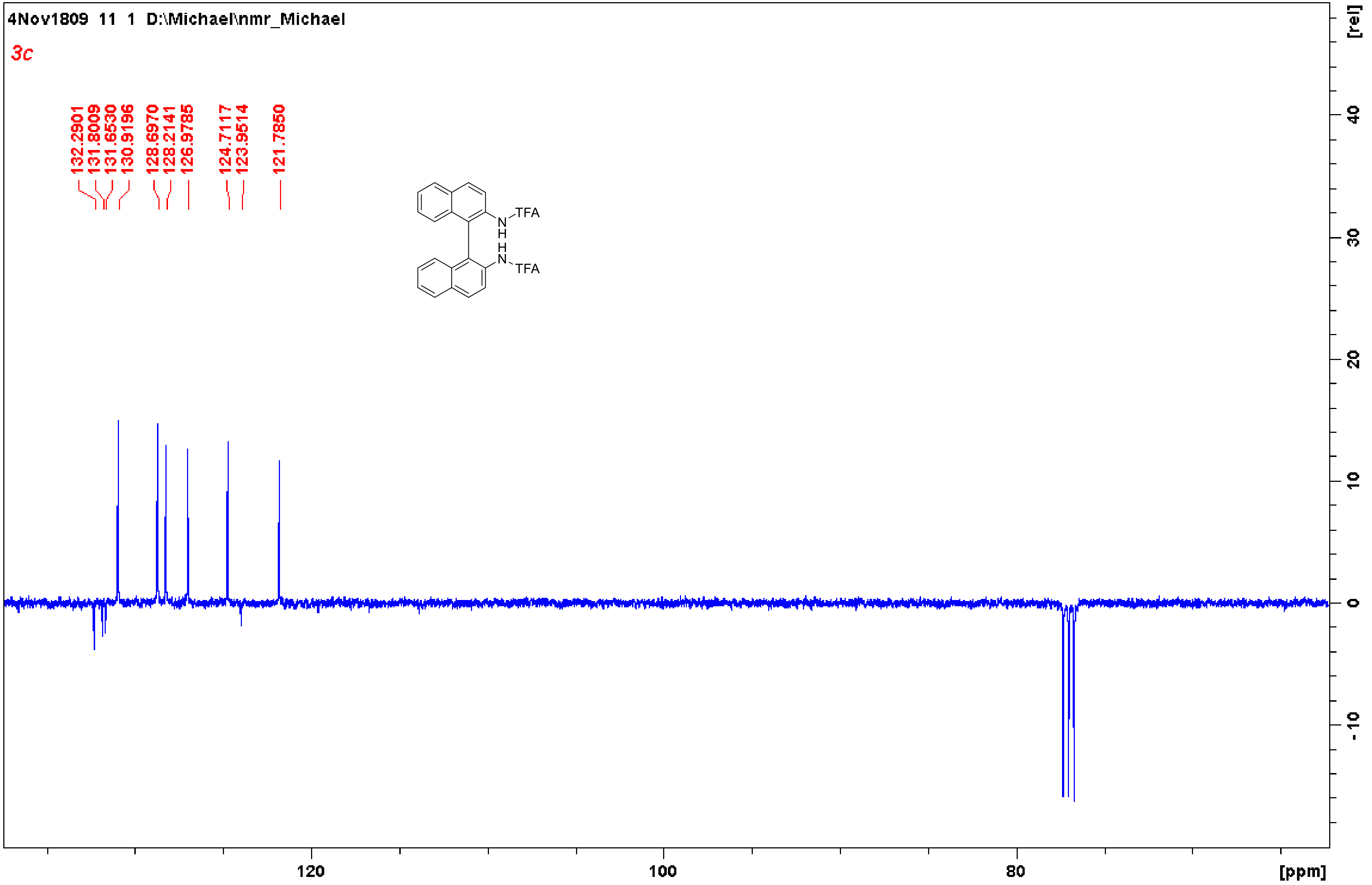
4Nov1809 11 1 D:\Michael\nmr_Michael
3c
132.2901
131.8009
131.6530
130.9196
128.6970
128.2141
126.9785
124.7117
123.9514
121.7850
TFA
N
H
TFA
[rel]
40
30
20
10
0
-10
120
100
80
[ppm]

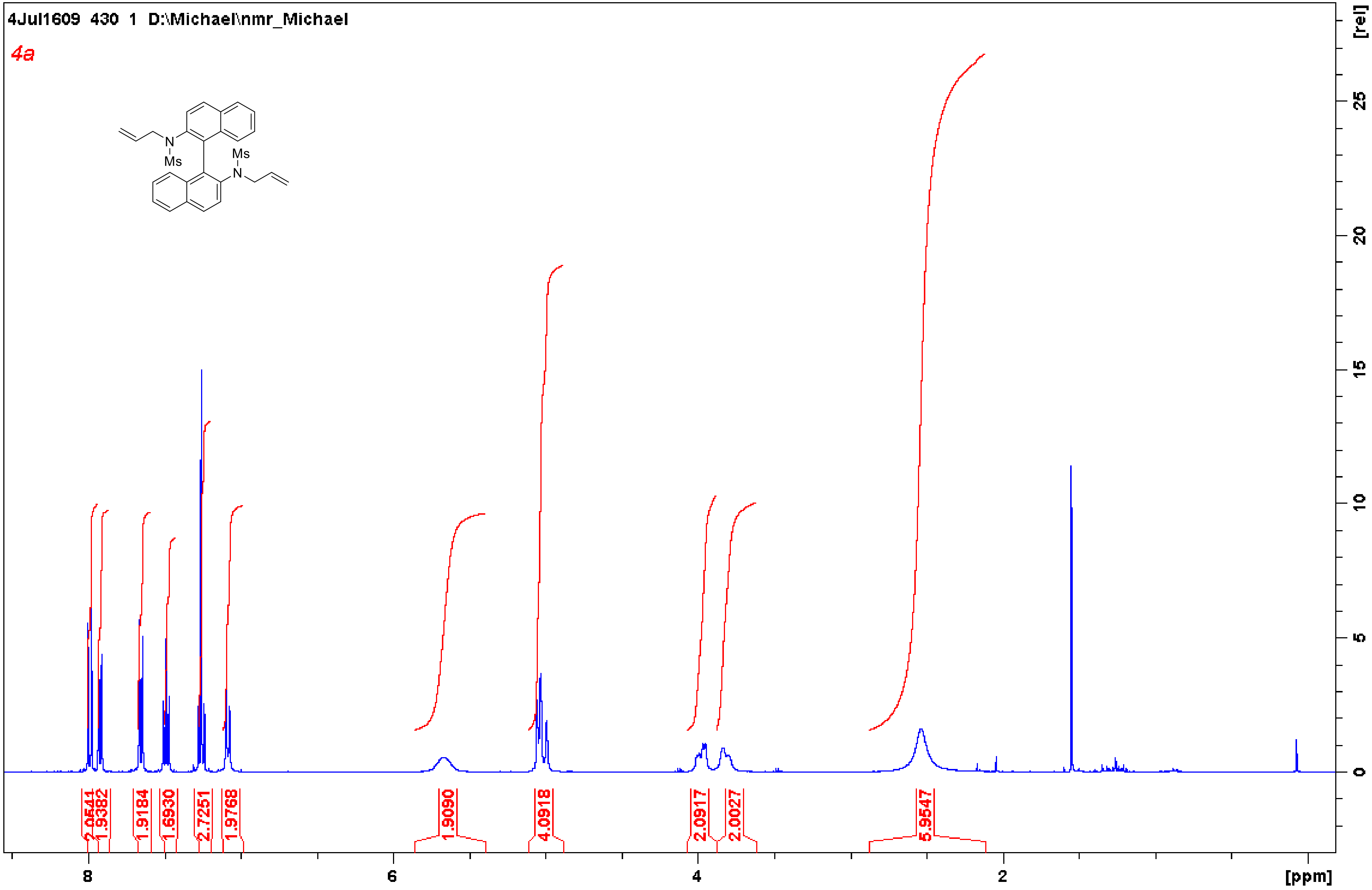
4Jul1609 430 1 D:\Michael\nmr_Michael
4a
Ms
N
Ms
N
2.0541
1.9382
1.9184
1.6930
2.7251
1.9768
1.9090
4.0918
2.0917
2.0027
5.9547
[rel]
25
20
15
10
5
0
8
6
4
2
[ppm]

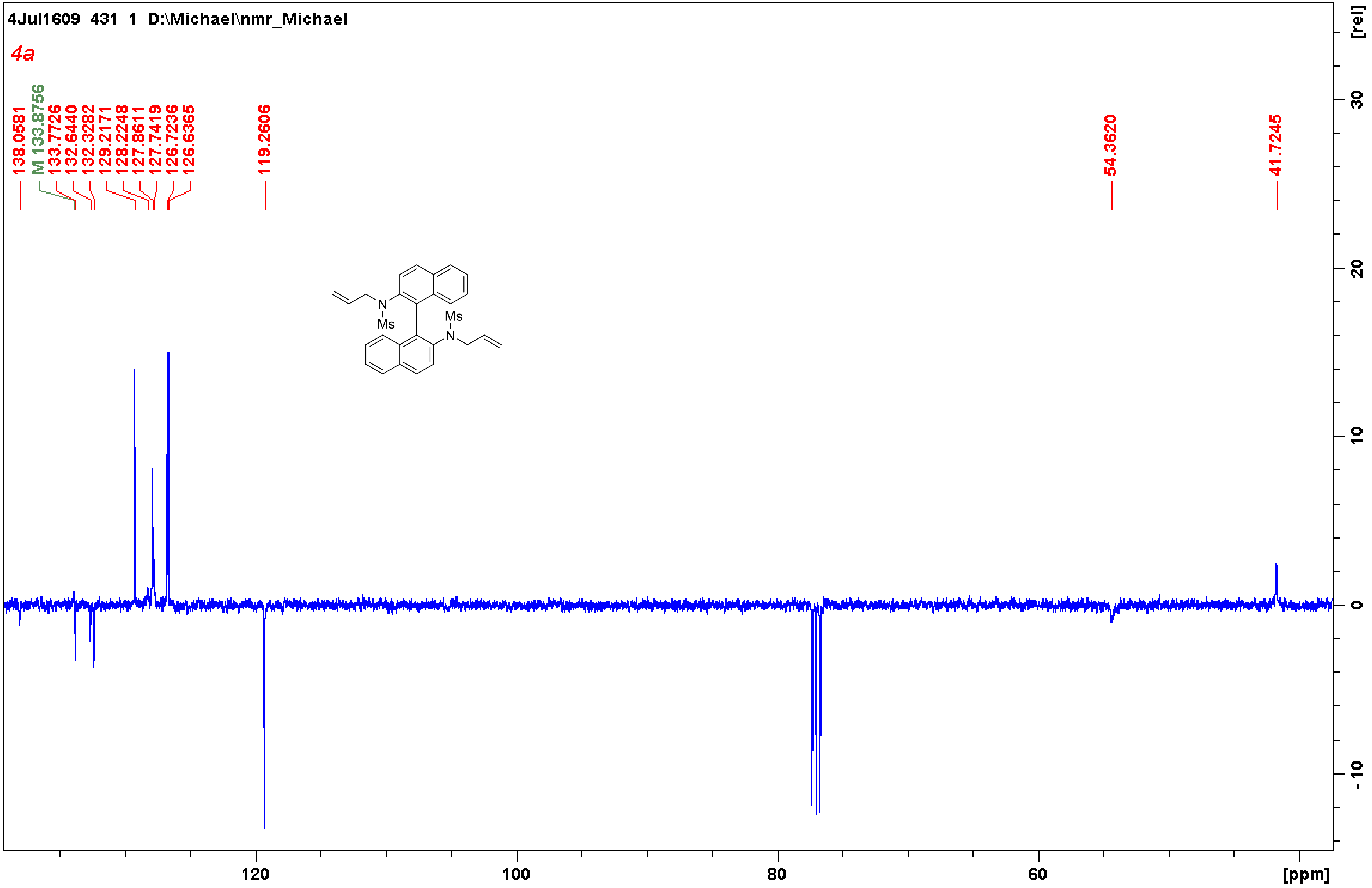
4Jul1609 431 1 D:\Michael\nmr_Michael
4a
138.0581
M 133.8756
133.7726
132.6440
132.3282
129.2171
128.2248
127.8611
127.7419
126.7236
126.6365
119.2606
54.3620
41.7245
N
Ms
Ms
N
[rel]
30
20
10
0
-10
120
100
80
60
[ppm]

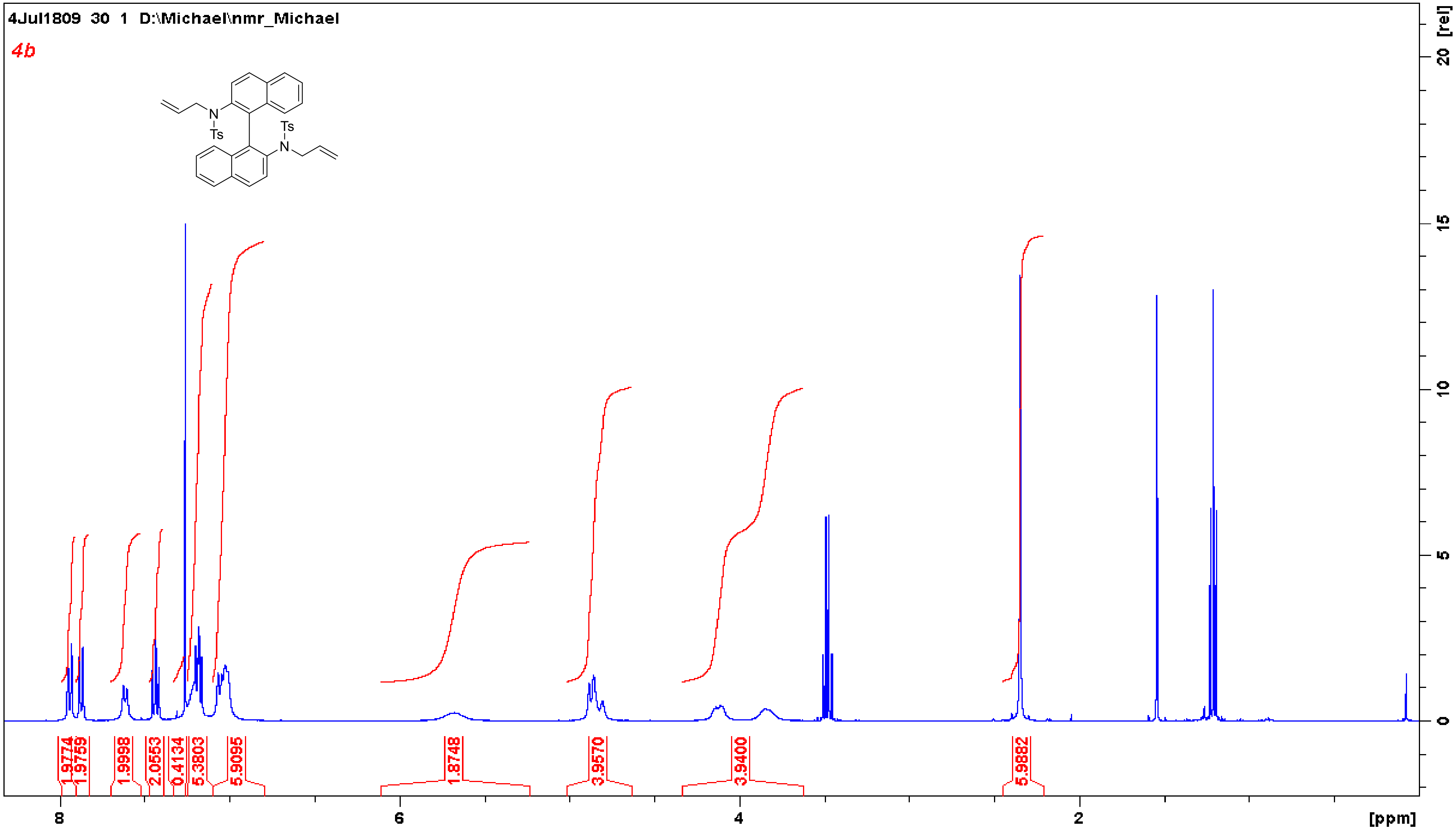
4Jul1809 30 1 D:\Michael\nmr_Michael
4b
N
Ts
Ts
N
1.9774
1.9759
1.9998
2.0553
0.4134
5.3803
5.9095
1.8748
3.9570
3.9400
5.9882
8
6
4
2
[ppm]
0
5
10
15
20
[rel]

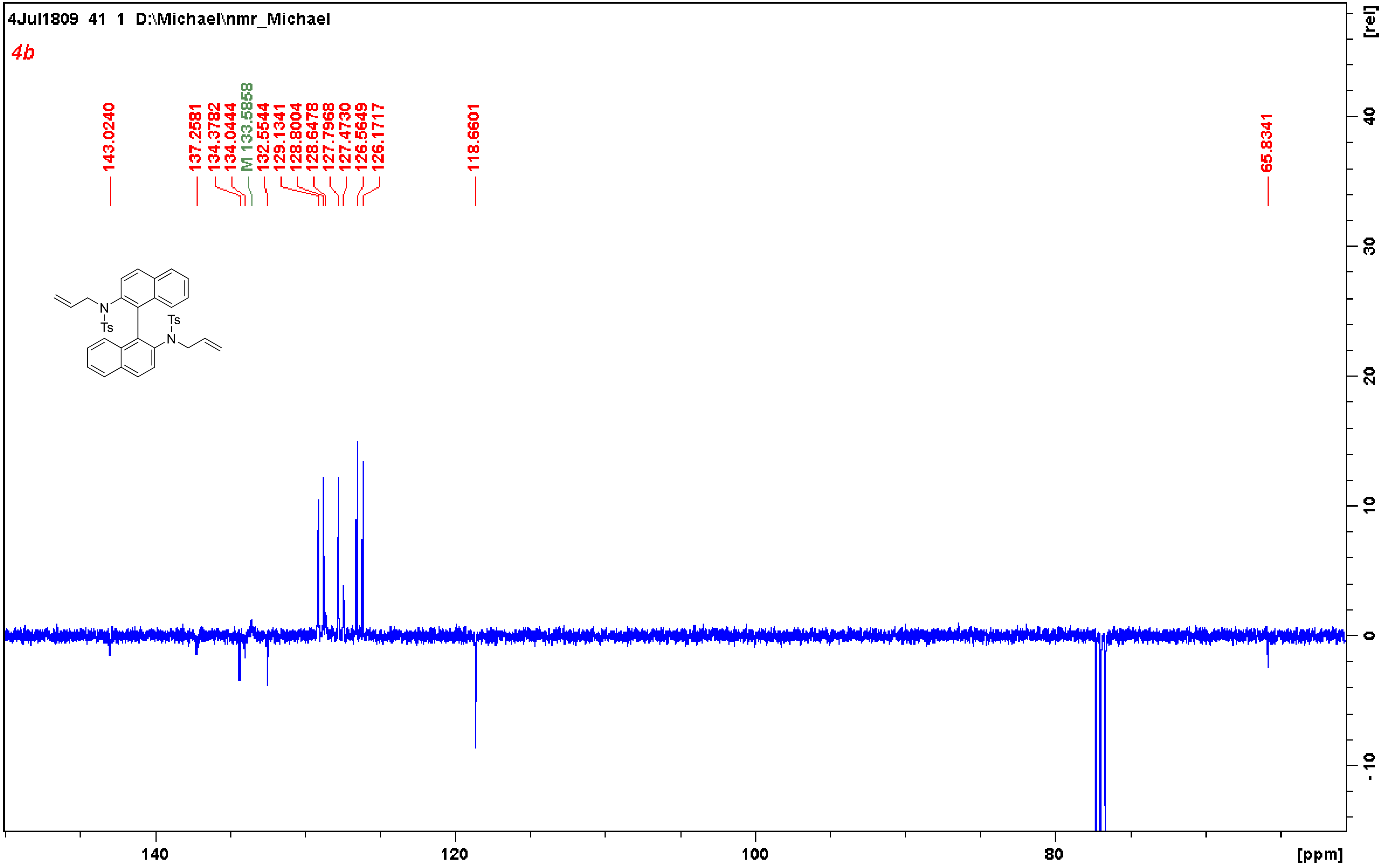
4Jul1809 41 1 D:\Michael\nmr_Michael
4b
143.0240
137.2581
134.3782
134.0444
M 133.5858
132.5544
129.1341
128.8004
128.6478
127.7968
127.4730
126.5649
126.1717
118.6601
65.8341
Ts
N
[rel]
40
30
20
10
0
- 10
140
120
100
80
[ppm]

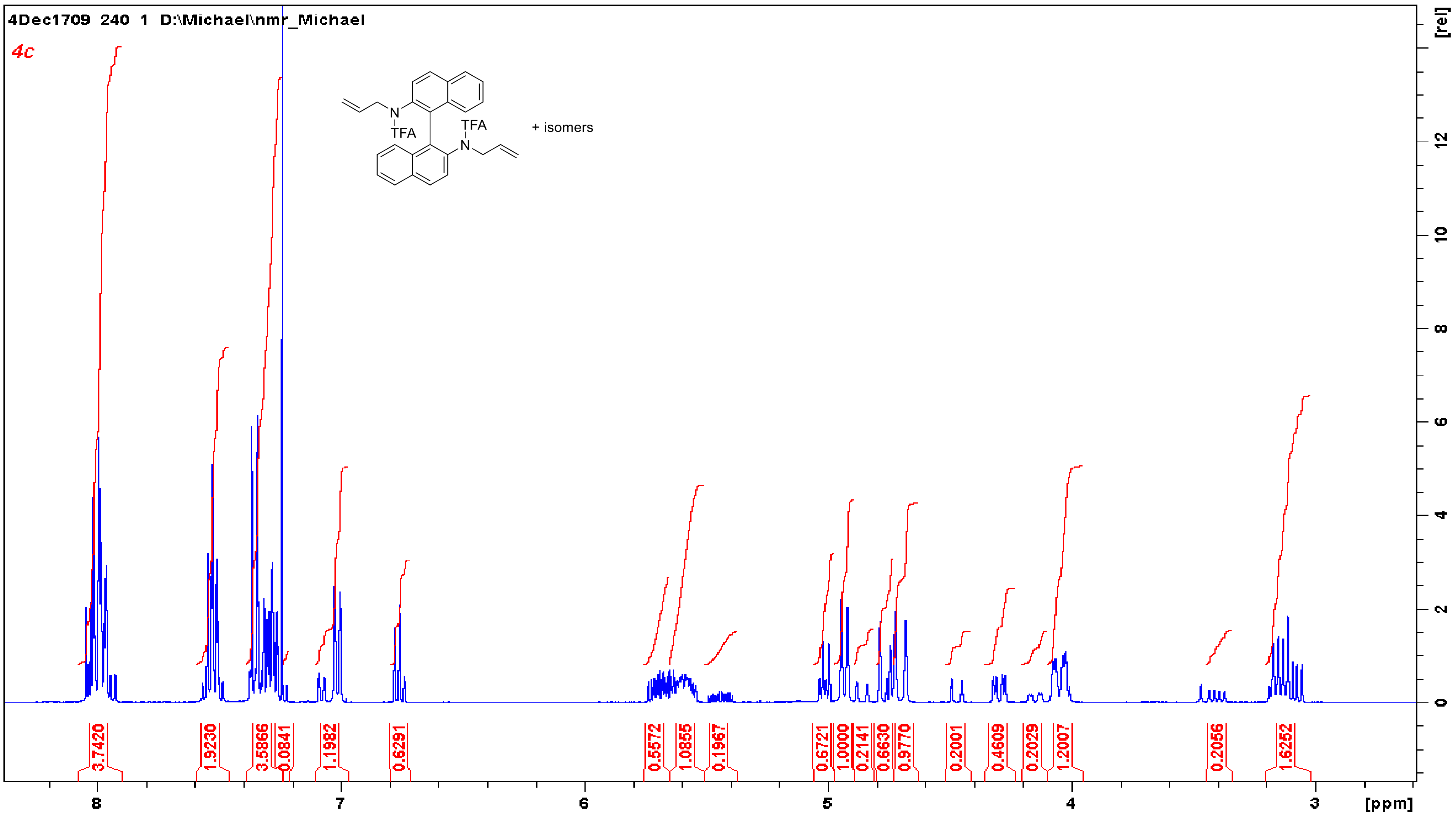
4Dec1709 240 1 D:\Michael\nmr_Michael
4c
TFA
N
TFA
N
+ isomers
[rel]
[ppm]
3.7420
1.9230
3.5866
0.0841
1.1982
0.6291
0.5572
1.0855
0.1967
0.6721
1.0000
0.2141
0.6630
0.9770
0.2001
0.4609
0.2029
1.2007
0.2056
1.6252

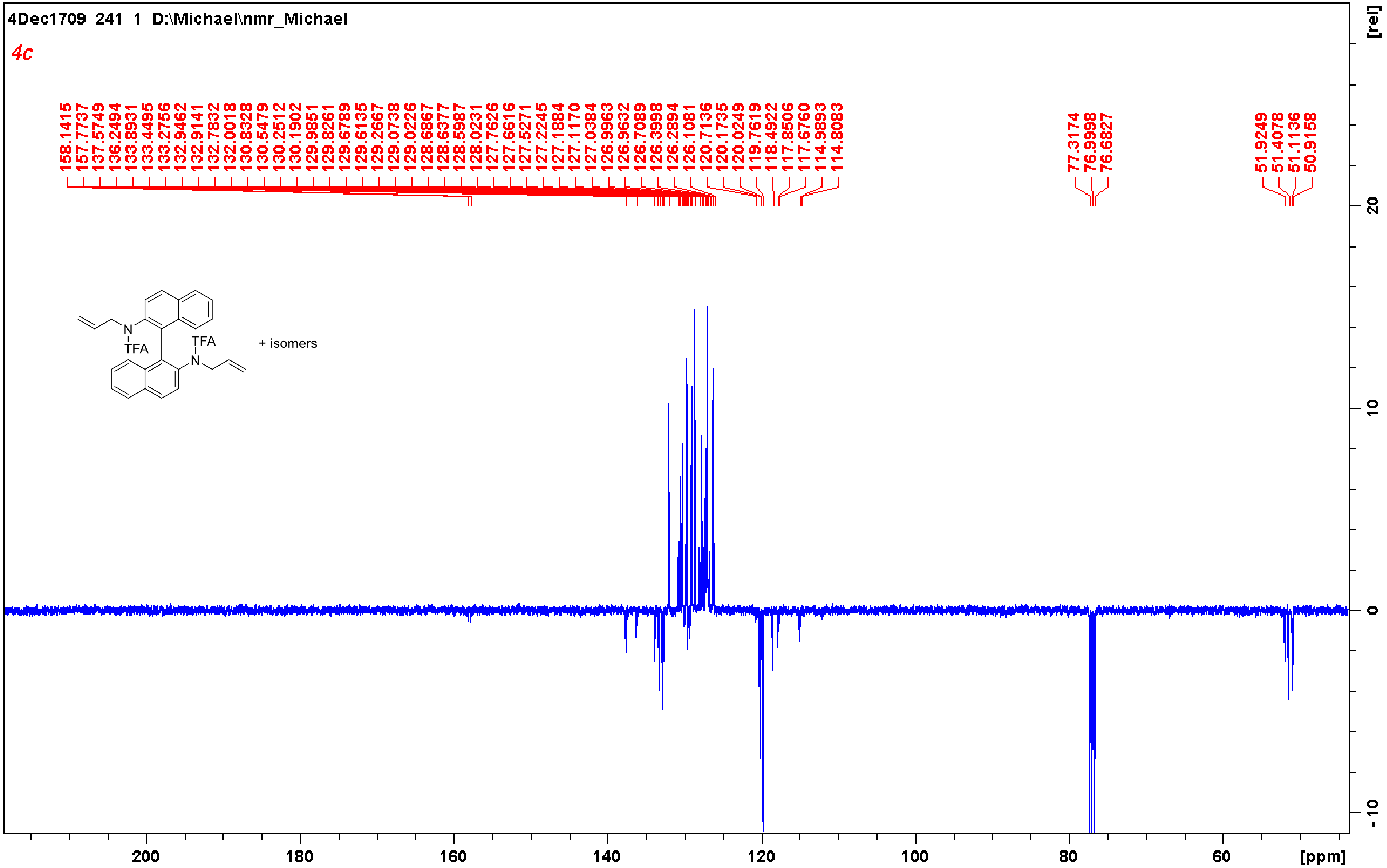
4Dec1709 241 1 D:\Michael\nmr_Michael
4c
158.1415
157.7737
137.5749
136.2494
133.8931
133.4495
133.2756
132.9462
132.9141
132.7832
132.0018
130.8328
130.5479
130.2512
130.1902
129.9851
129.8261
129.6789
129.6135
129.2667
129.0738
129.0226
128.6867
128.6377
128.5987
128.0231
127.7626
127.6616
127.5271
127.2245
127.1884
127.1170
127.0384
126.9963
126.9632
126.7089
126.3998
126.2894
126.1081
120.7136
120.1735
120.0249
119.7619
118.4922
117.8506
117.6760
114.9893
114.8083
77.3174
76.9998
76.6827
51.9249
51.4078
51.1136
50.9158
N
TFA
TFA
N
+ isomers
[rel]
20
10
0
-10
200
180
160
140
120
100
80
60
[ppm]

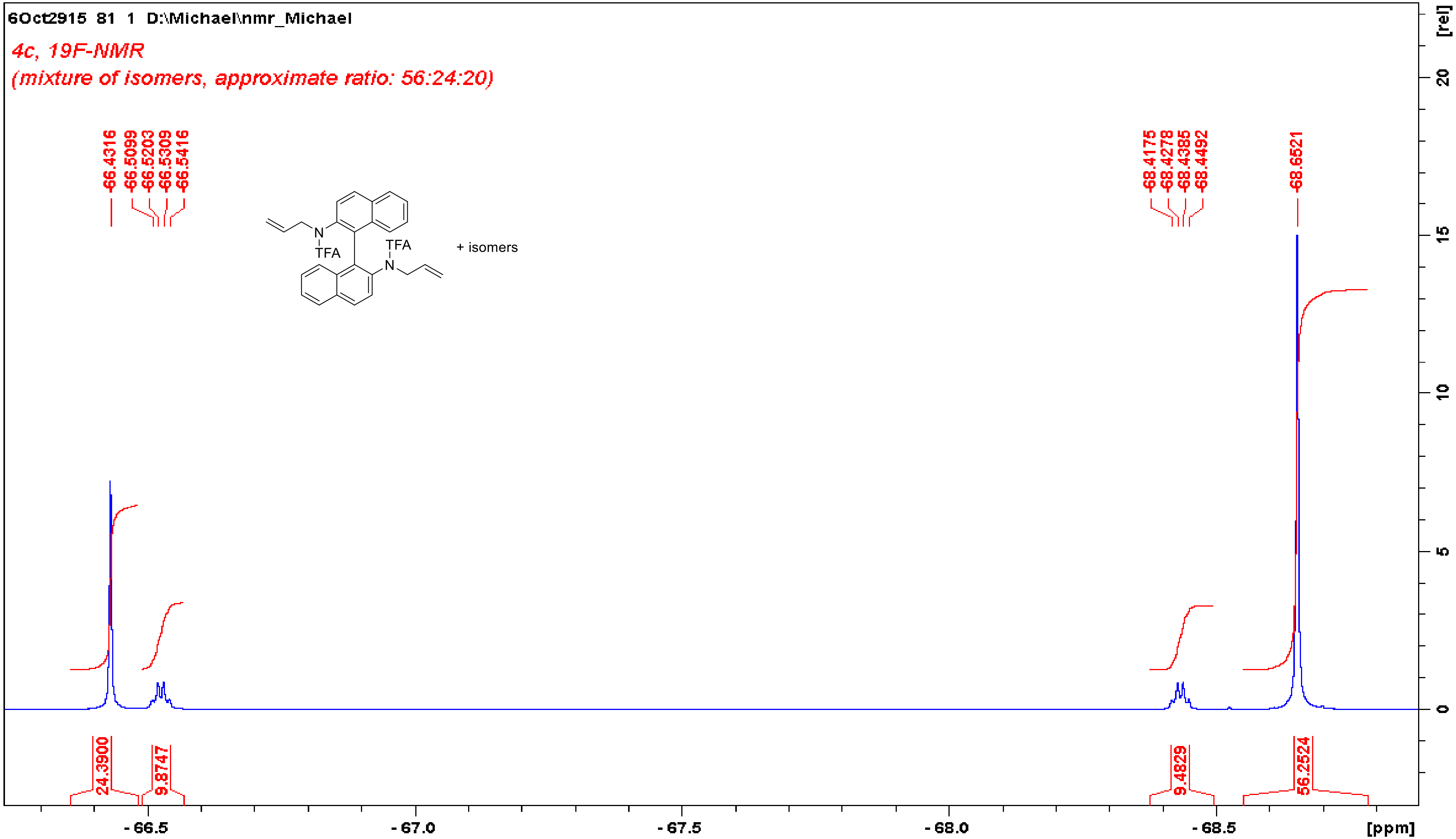
6Oct2915 81 1 D:\Michael\nmr_Michael
4c, 19F-NMR
(mixture of isomers, approximate ratio: 56:24:20)
-66.4316
-66.5099
-66.5203
-66.5309
-66.5416
-68.4175
-68.4278
-68.4385
-68.4492
-68.6521
N
TFA
TFA
N
+ isomers
24.3900
9.8747
9.4829
56.2524
- 66.5
- 67.0
- 67.5
- 68.0
- 68.5
[ppm]
[rel]
20
15
10
5
0

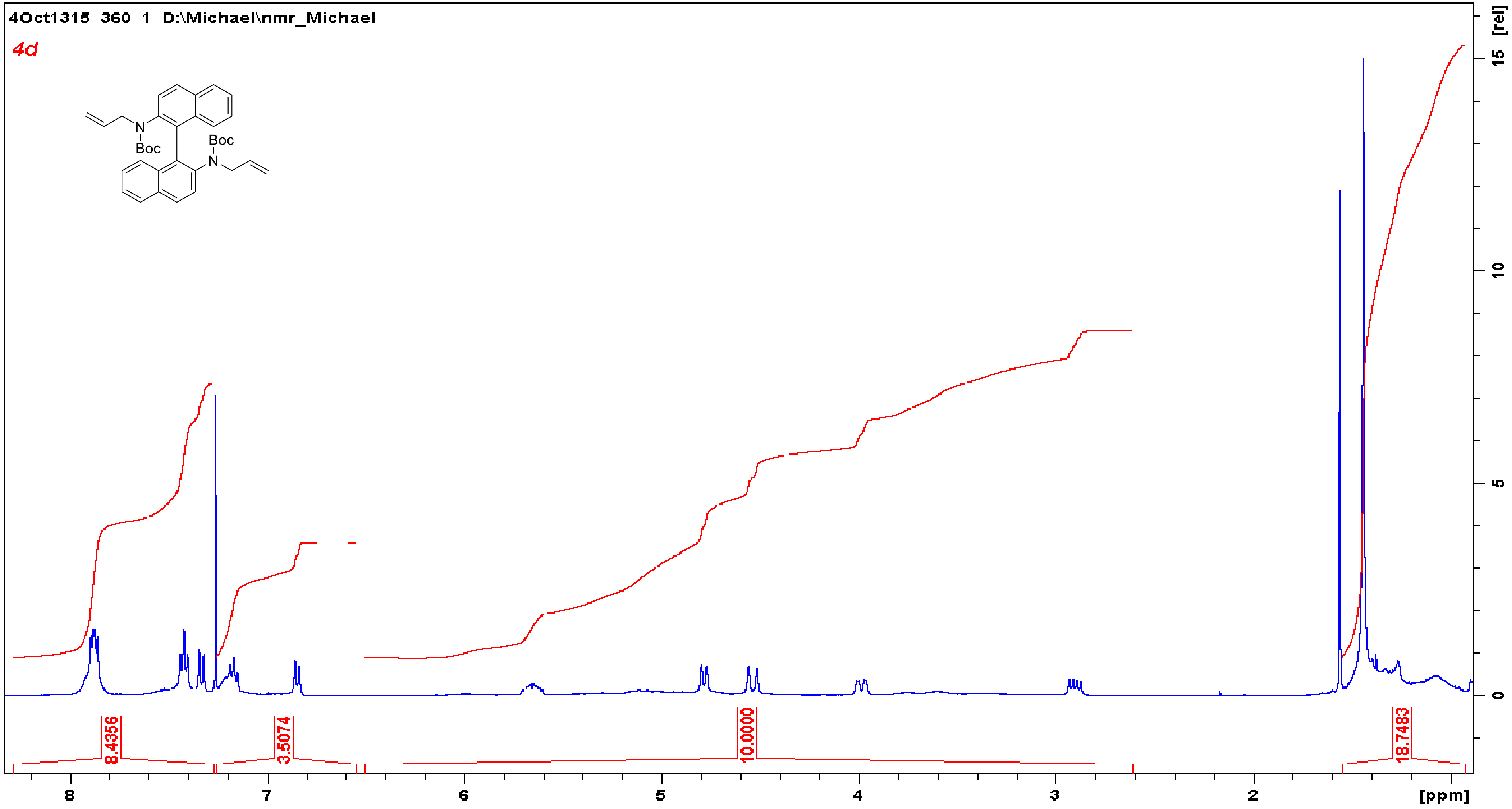
4Oct1315 360 1 D:\Michael\nmr_Michael
4d
N
Boc
Boc
N
8.4356
3.5074
10.0000
18.7483
8
7
6
5
4
3
2
[ppm]
0
5
10
15
[rel]

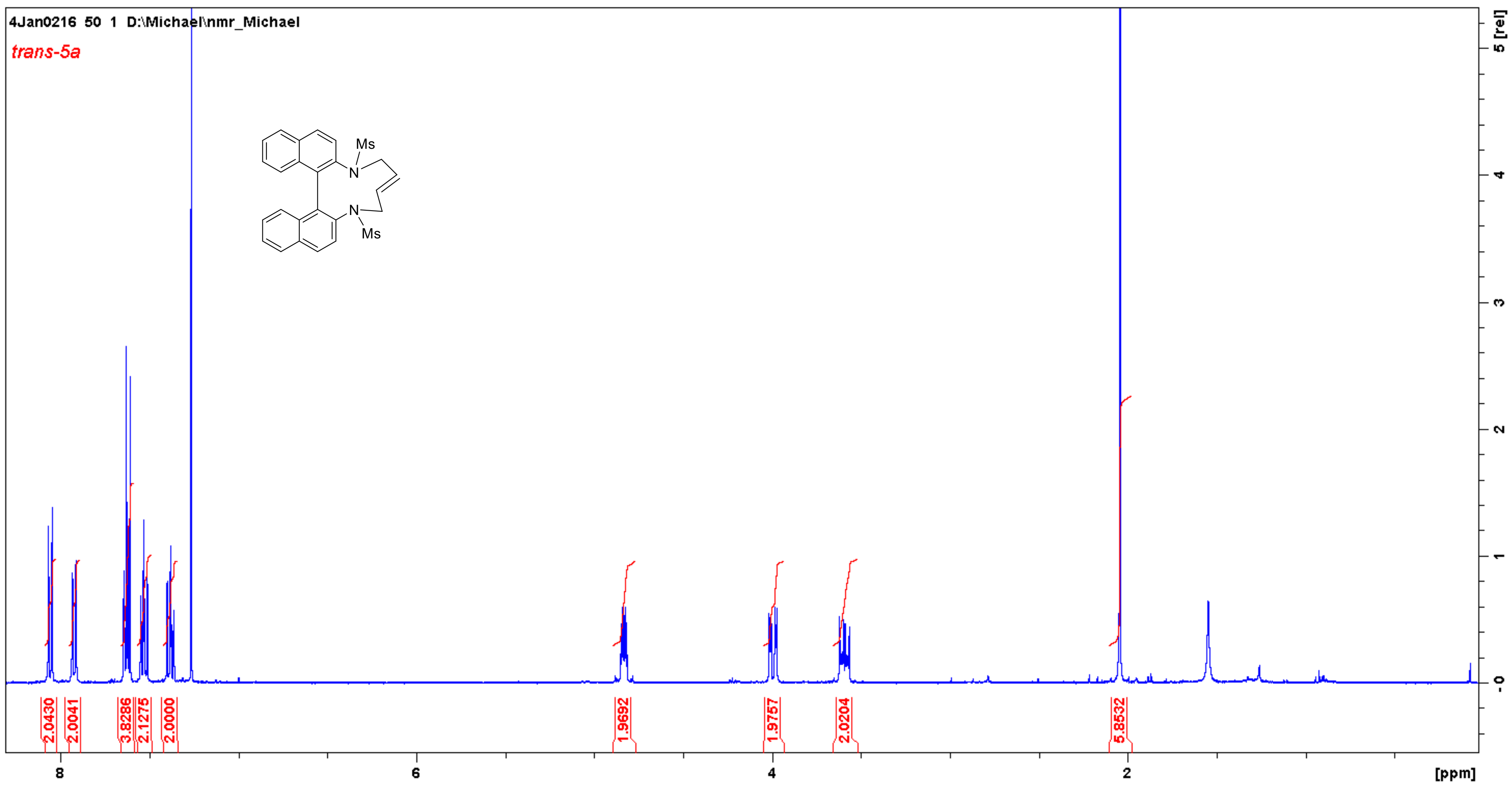
4Jan0216 50 1 D:\Michael\nmr_Michael
trans-5a
Ms
N
N
Ms
2.0430
2.0041
3.8286
2.1275
2.0000
1.9692
1.9757
2.0204
5.8532
8
6
4
2
[ppm]
5 [rel]
4
3
2
1
-0

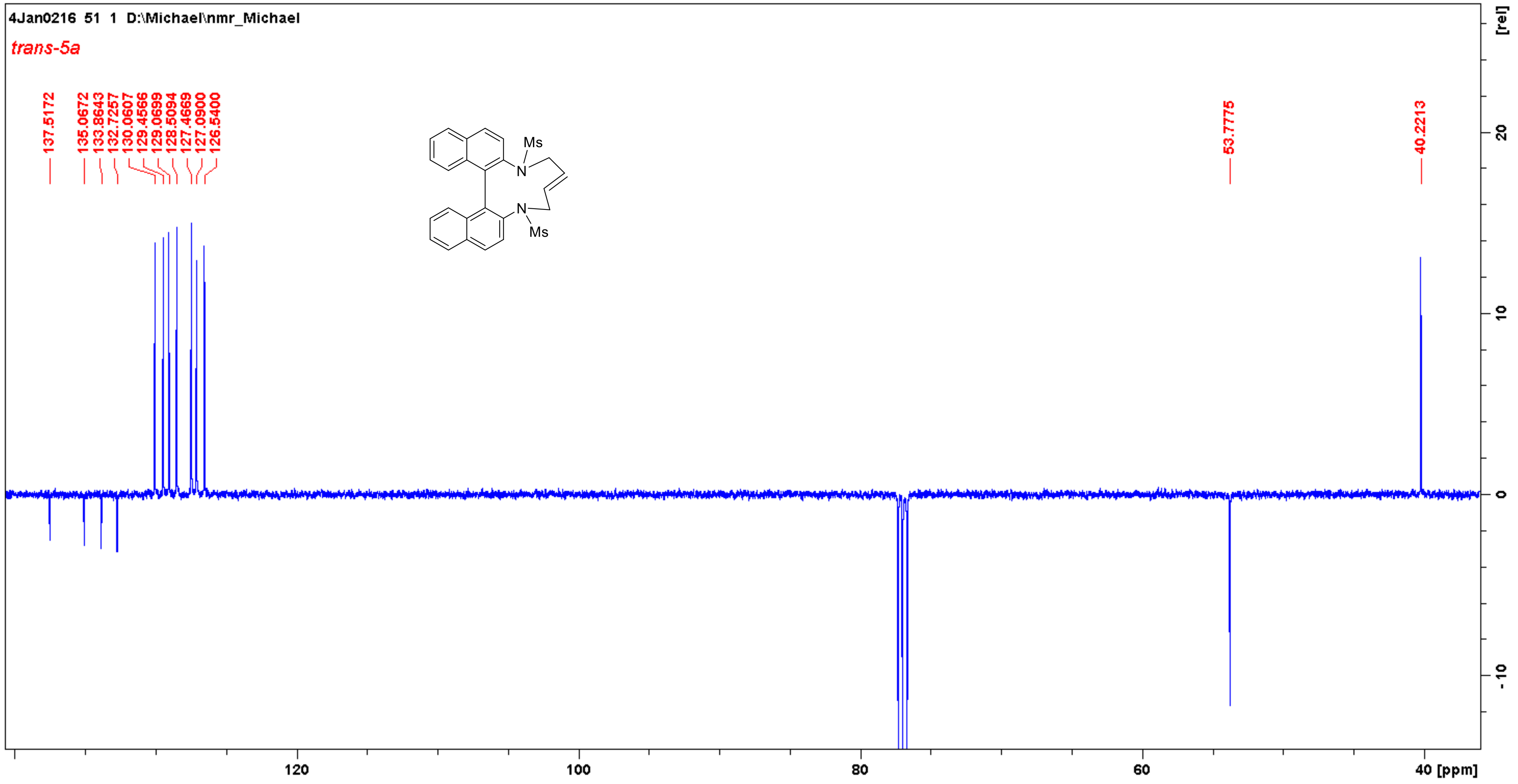
4Jan0216 51 1 D:\Michael\nmr_Michael
trans-5a
137.5172
135.0672
133.8643
132.7257
130.0607
129.4566
129.0699
128.5094
127.4669
127.0900
126.5400
53.7775
40.2213
Ms
N
N
Ms
120
100
80
60
40 [ppm]
[rel]
20
10
0
-10

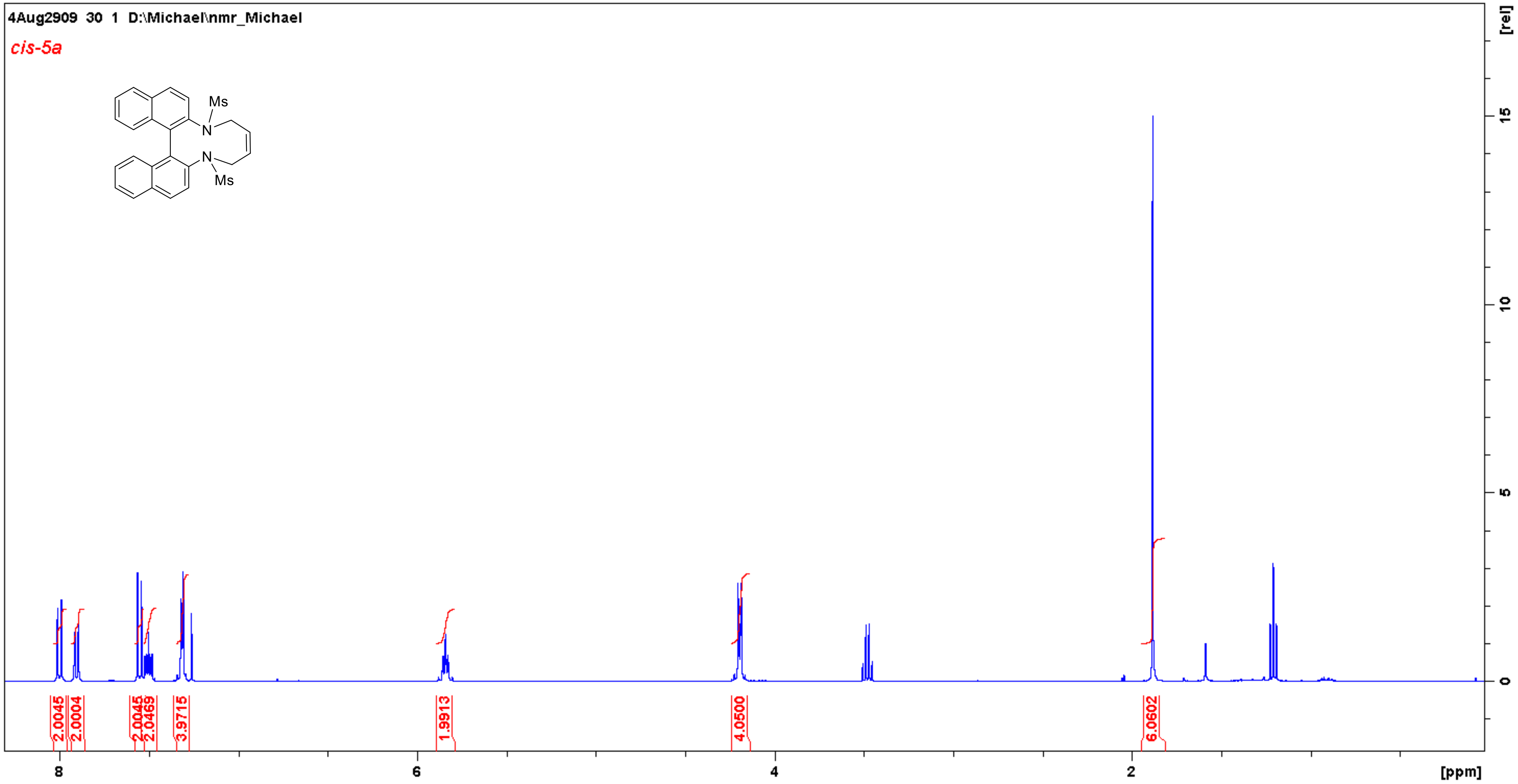

4Aug2909 30 1 D:\Michael\nmr_Michael
cis-5a
Ms
N
N
Ms
2.0045
2.0004
2.0045
2.0469
3.9715
1.9913
4.0500
6.0602
8
6
4
2
[ppm]
[rel]
0
5
10
15

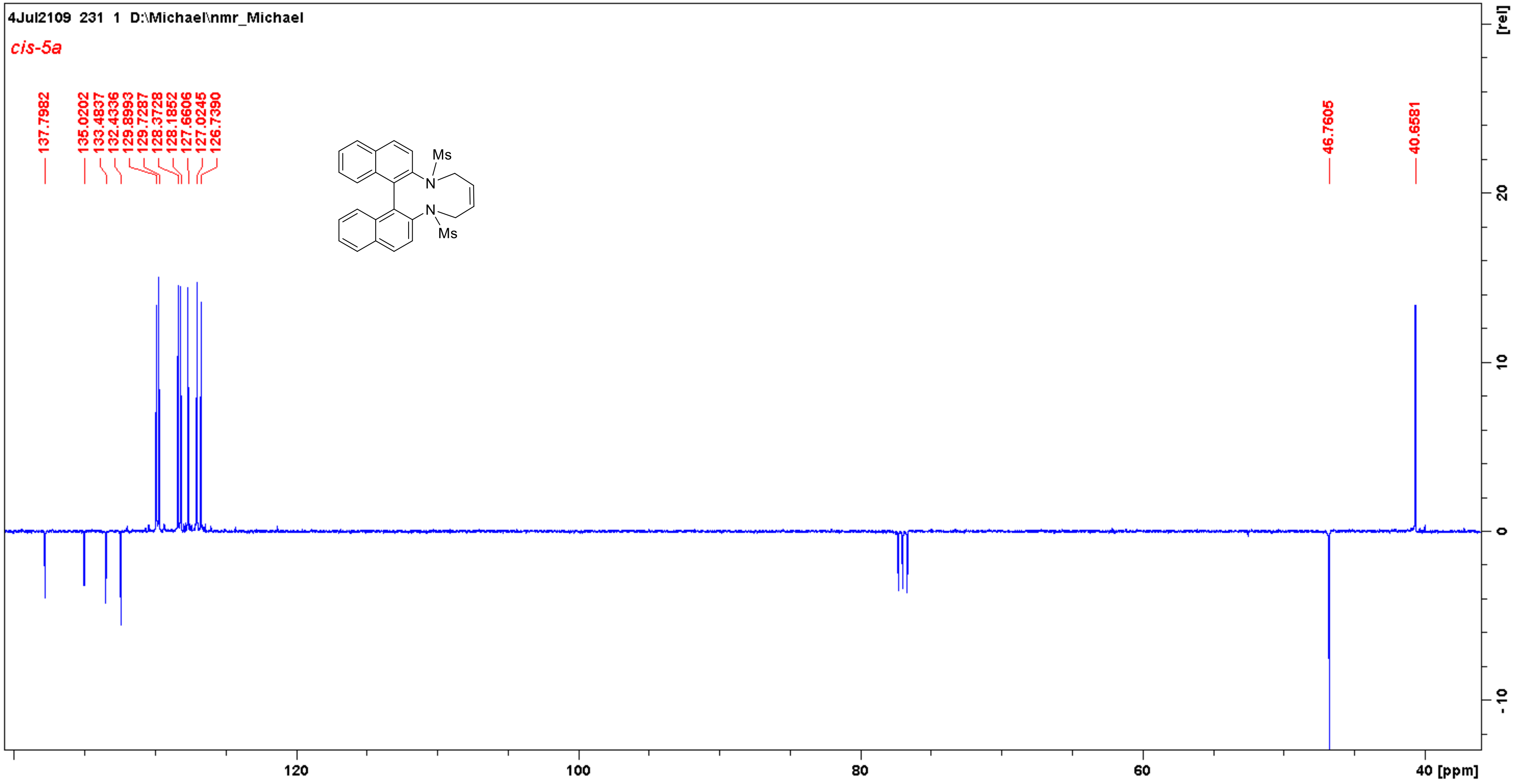
4Jul2109 231 1 D:\Michael\nmr_Michael
cis-5a
137.7982
135.0202
133.4837
132.4336
129.8993
129.7287
128.3728
128.1852
127.6606
127.0245
126.7390
46.7605
40.6581
Ms
N
N
Ms
[rel]
20
10
0
-10
120
100
80
60
40 [ppm]

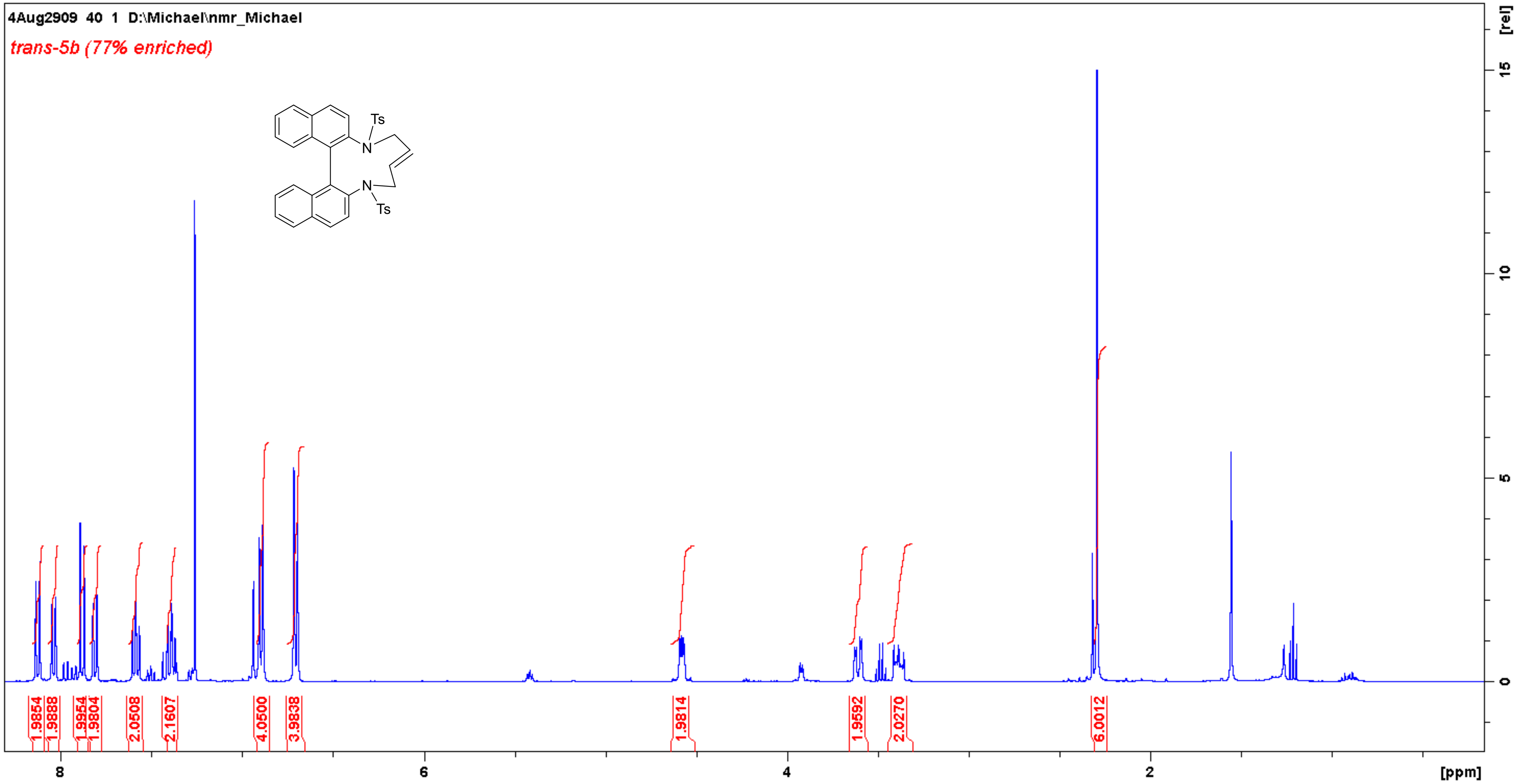
4Aug2909 40 1 D:\Michael\nmr_Michael
trans-5b (77% enriched)
Ts
N
N
Ts
1.9854
1.9888
1.9954
1.9804
2.0508
2.1607
4.0500
3.9838
1.9814
1.9592
2.0270
6.0012
8
6
4
2
[ppm]
[rel]
15
10
5
0

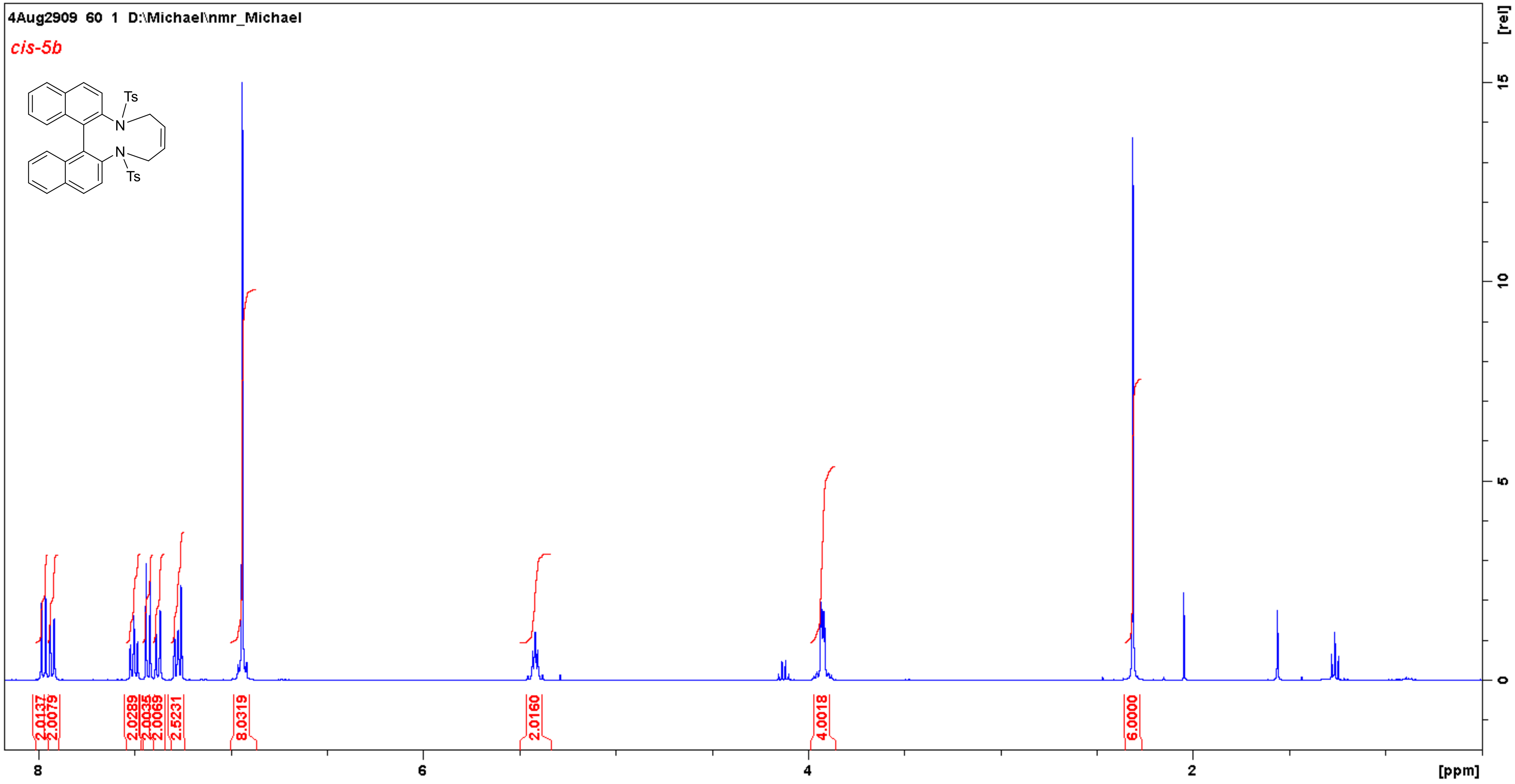
4Aug2909 60 1 D:\Michael\nmr_Michael
cis-5b
Ts
N
N
Ts
[rel]
15
10
5
0
2.0137
2.0079
2.0289
2.0035
2.0069
2.5231
8.0319
2.0160
4.0018
6.0000
8
6
4
2
[ppm]

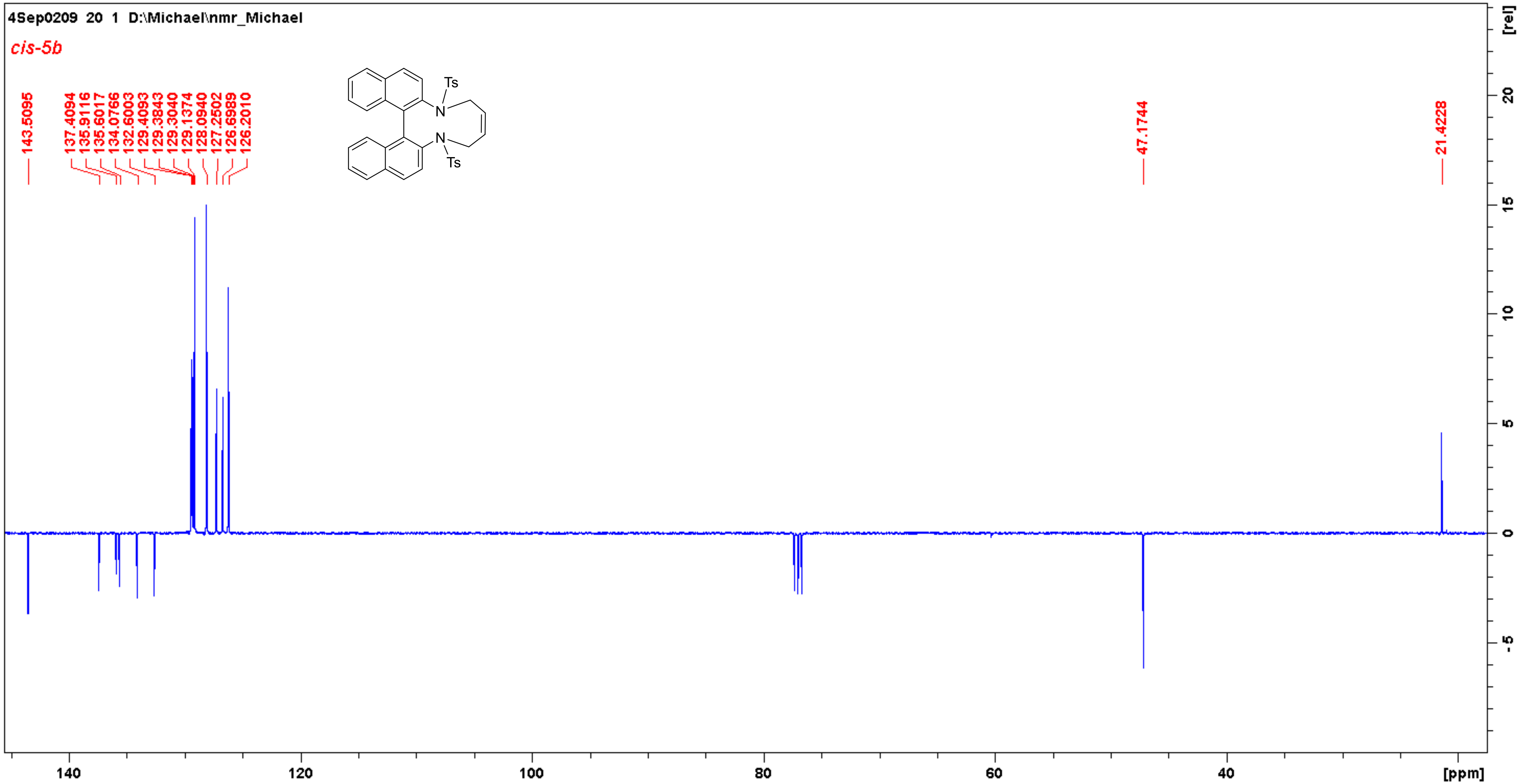
4Sep0209 20 1 D:\Michael\nmr_Michael
cis-5b
143.5095
137.4094
135.9116
135.6017
134.0766
132.6003
129.4093
129.3843
129.3040
129.1374
128.0940
127.2502
126.6989
126.2010
47.1744
21.4228
Ts
N
N
Ts
[rel]
20
15
10
5
0
-5
140
120
100
80
60
40
[ppm]

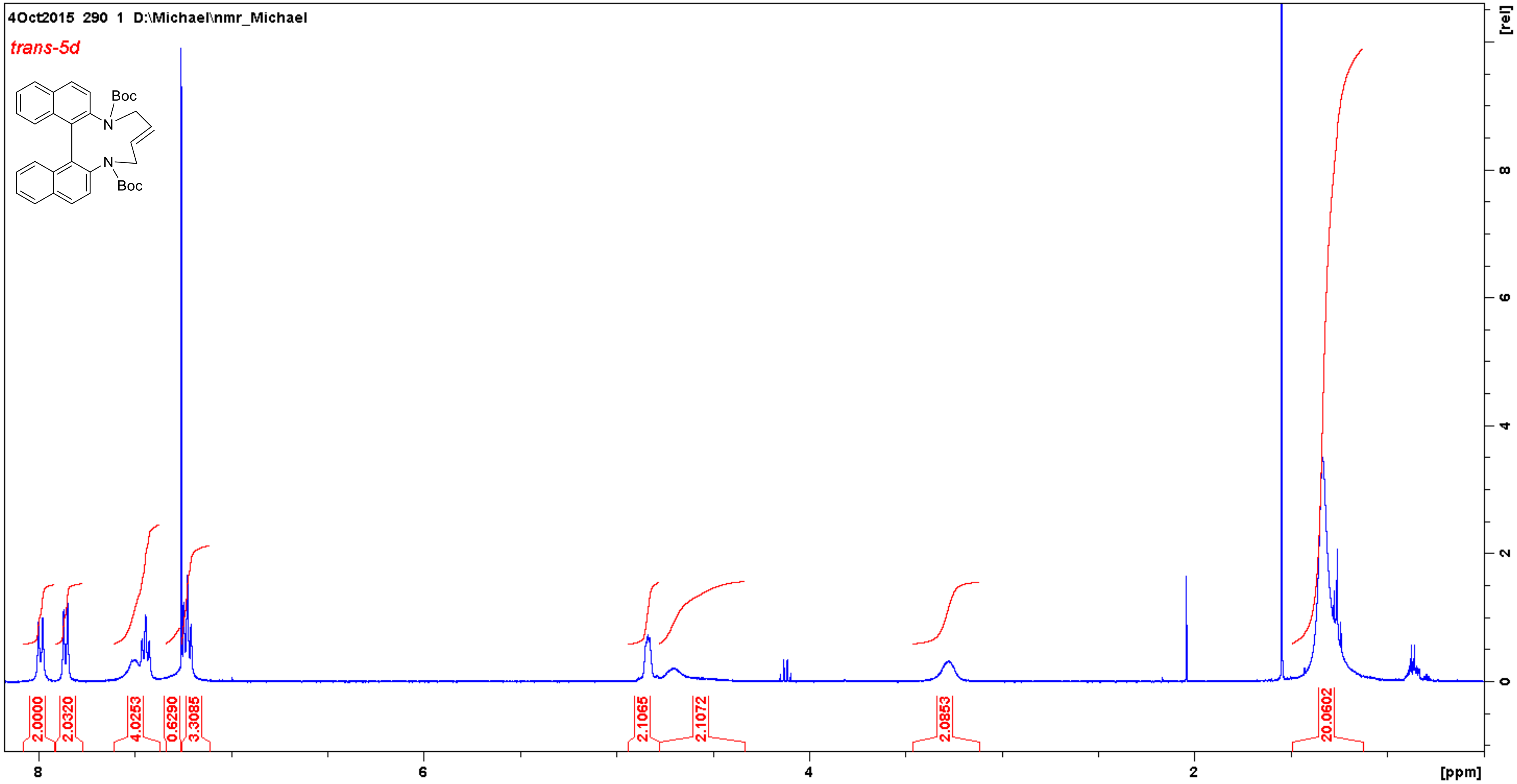

4Oct2015 290 1 D:\Michael\nmr_Michael
trans-5d
Boc
N
N
Boc
2.0000
2.0320
4.0253
0.6290
3.3085
2.1065
2.1072
2.0853
20.0602
8
6
4
2
[ppm]
[rel]
0
2
4
6
8

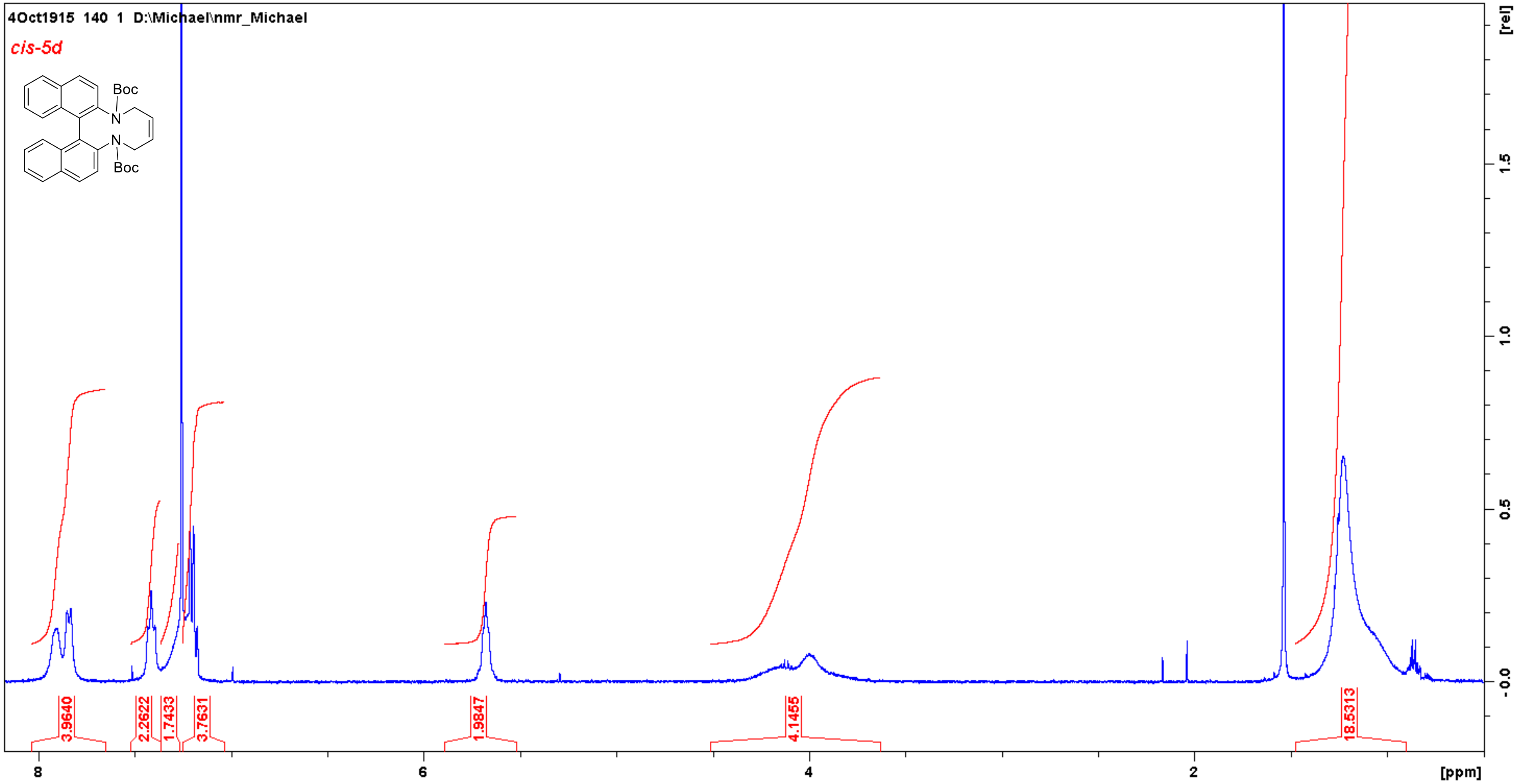
4Oct1915 140 1 D:\Michael\nmr_Michael
cis-5d
Boc
N
N
Boc
3.9640
2.2622
1.7433
3.7631
1.9847
4.1455
18.5313
8
6
4
2
[ppm]
[rel]
0.0
0.5
1.0
1.5

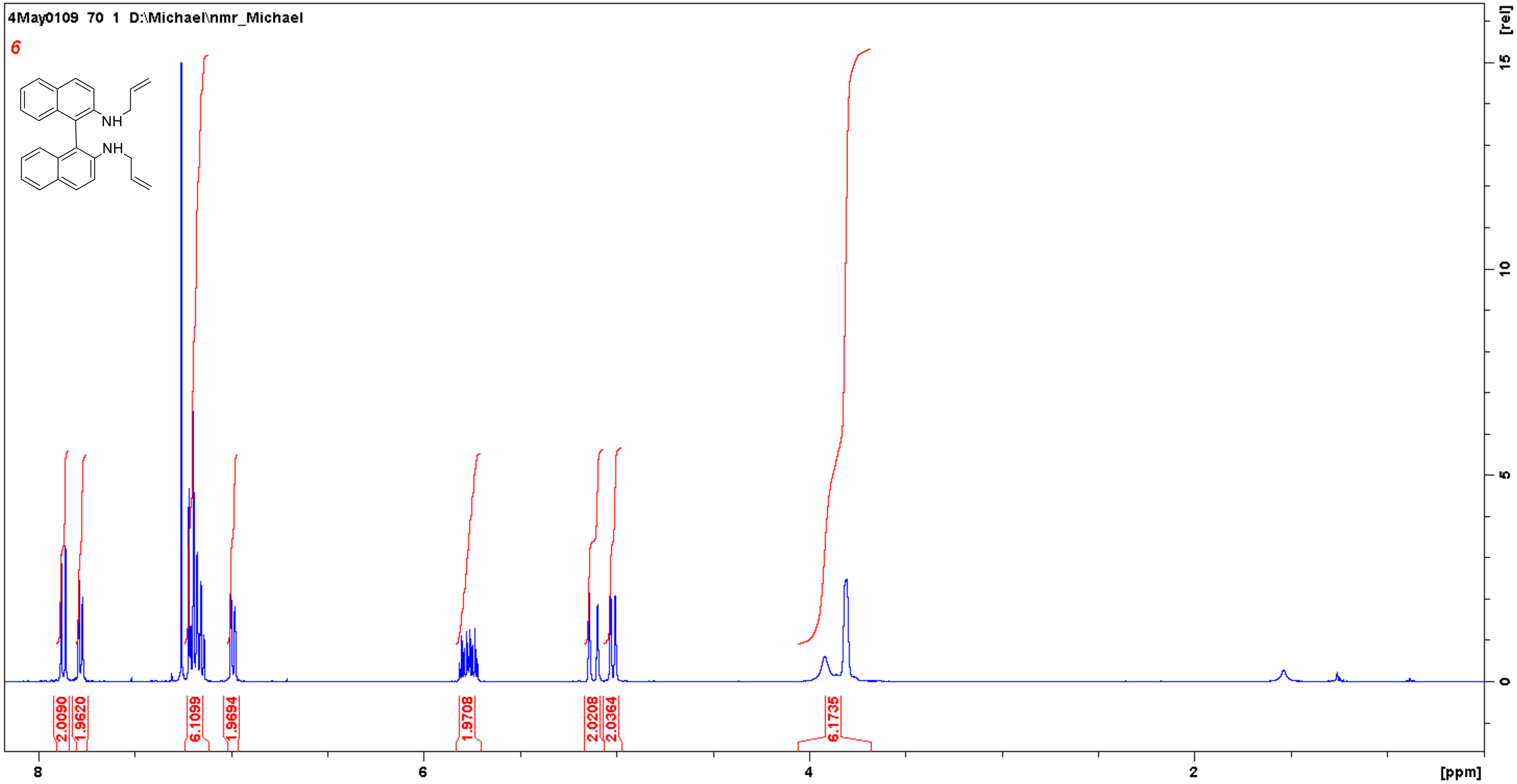
4May0109 70 1 D:\Michael\nmr_Michael
6
NH
NH
2.0090
1.9620
6.1099
1.9694
1.9708
2.0208
2.0364
6.1735
8
6
4
2
[ppm]
[rel]
15
10
5
0

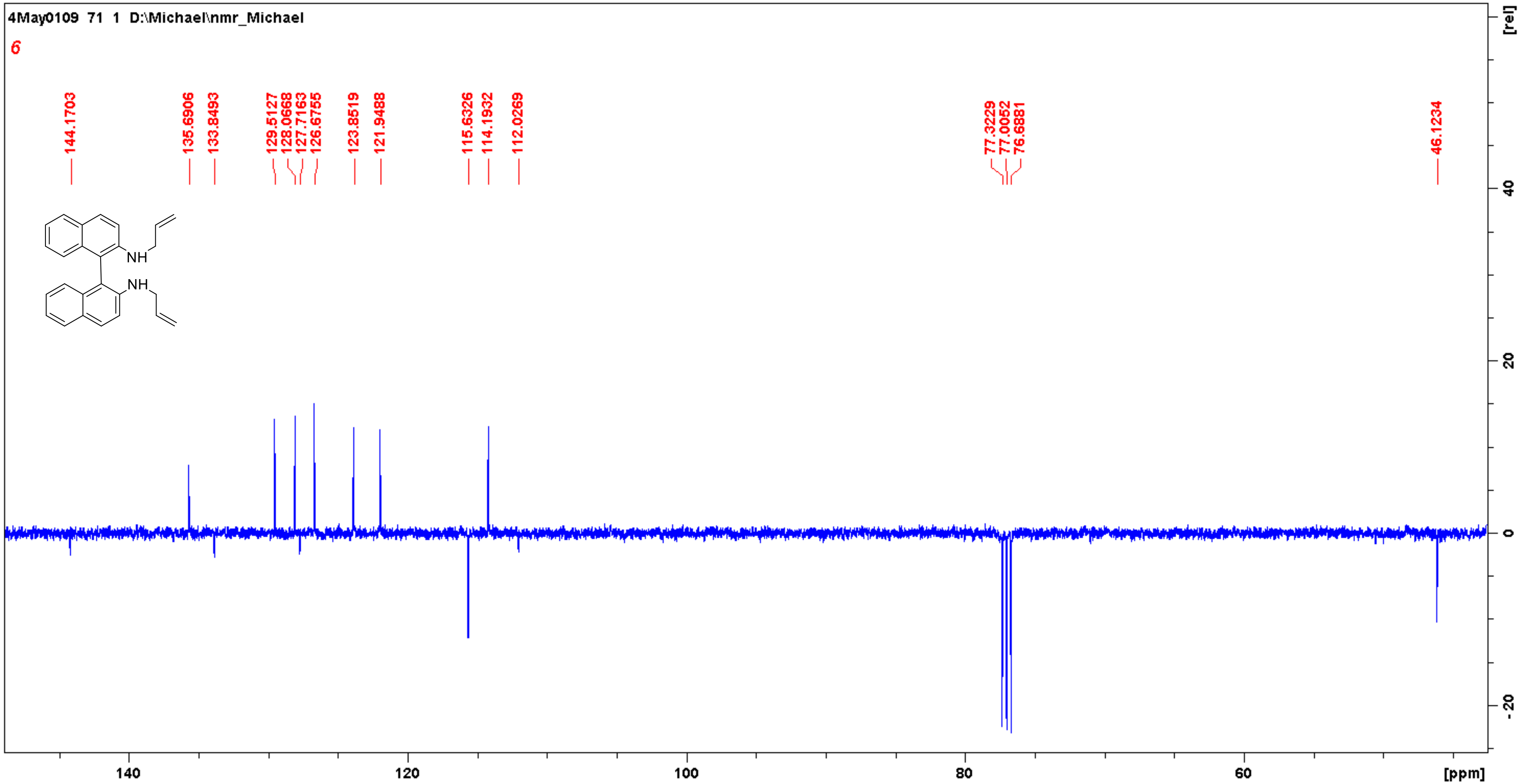
4May0109 71 1 D:\Michael\nmr_Michael
6
144.1703
135.6906
133.8493
129.5127
128.0668
127.7163
126.6755
123.8519
121.9488
115.6326
114.1932
112.0269
77.3229
77.0052
76.6881
46.1234
NH
NH
[rel]
40
20
0
-20
140
120
100
80
60
[ppm]

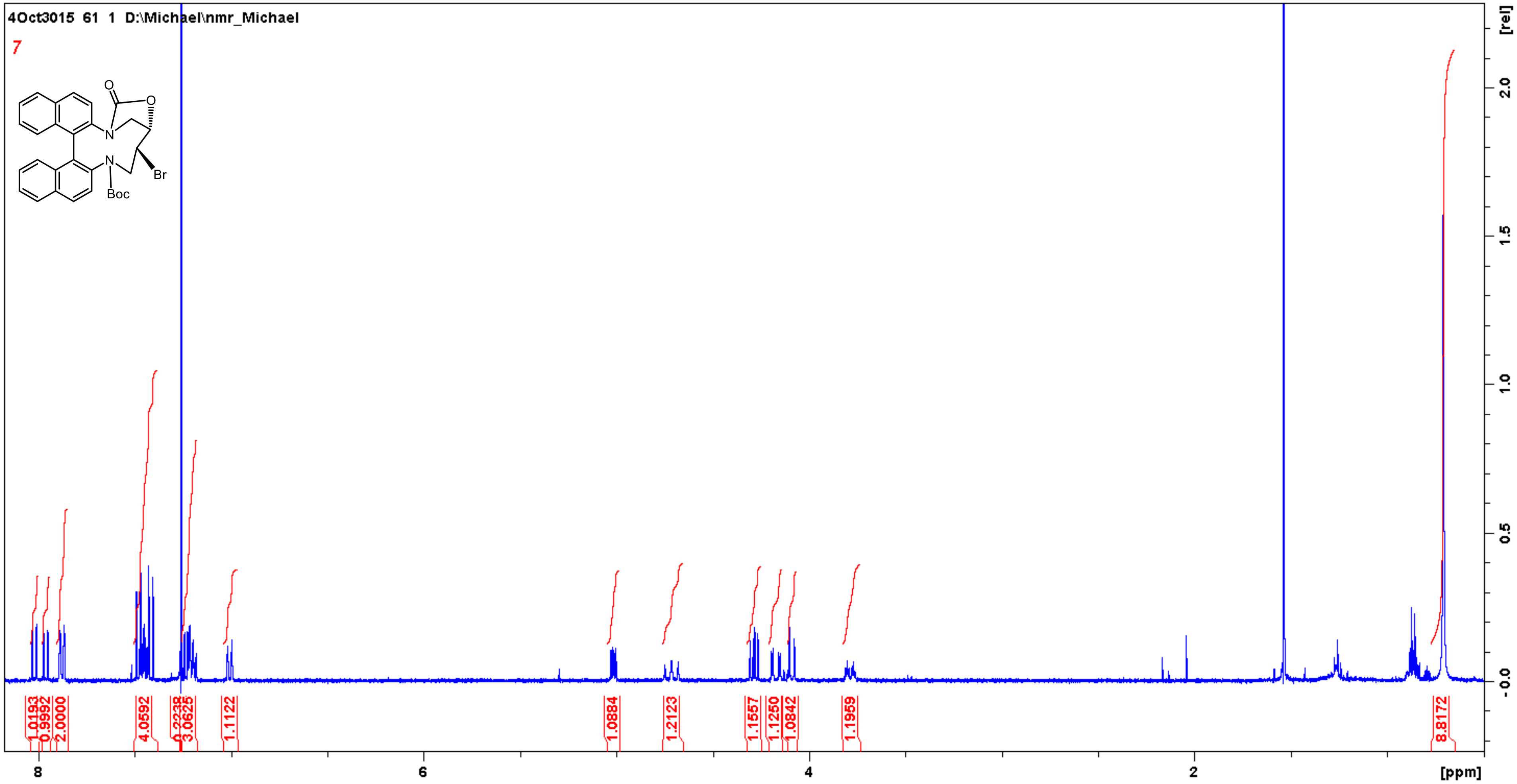
4Oct3015 61 1 D:\Michael\nmr_Michael
7
O
O
N
N
Br
Boc
1.0193
0.9992
2.0000
4.0592
0.2232
3.0625
1.1122
1.0884
1.2123
1.1557
1.1250
1.0842
1.1959
8.8172
[rel]
2.0
1.5
1.0
0.5
-0.0
8
6
4
2
[ppm]

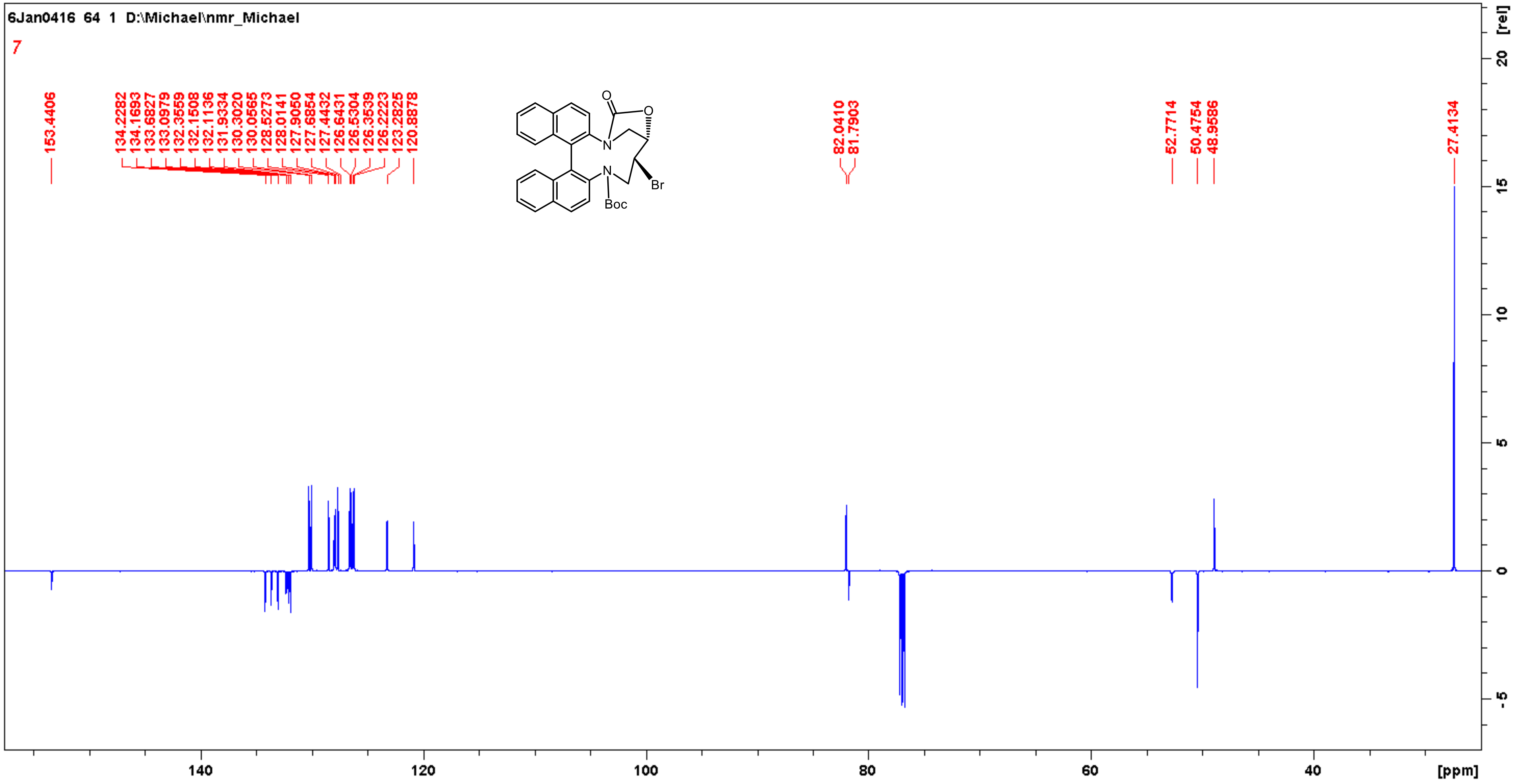
6Jan0416 64 1 D:\Michael\nmr_Michael
7
153.4406
134.2282
134.1693
133.6827
133.0979
132.3559
132.1508
132.1136
131.9334
130.3020
130.0565
128.5273
128.0141
127.9050
127.6854
127.4432
126.6431
126.5304
126.3539
126.2223
123.2825
120.8878
82.0410
81.7903
52.7714
50.4754
48.9586
27.4134
O
O
N
N
Br
Boc
[rel]
20
15
10
5
0
-5
140
120
100
80
60
40
[ppm]

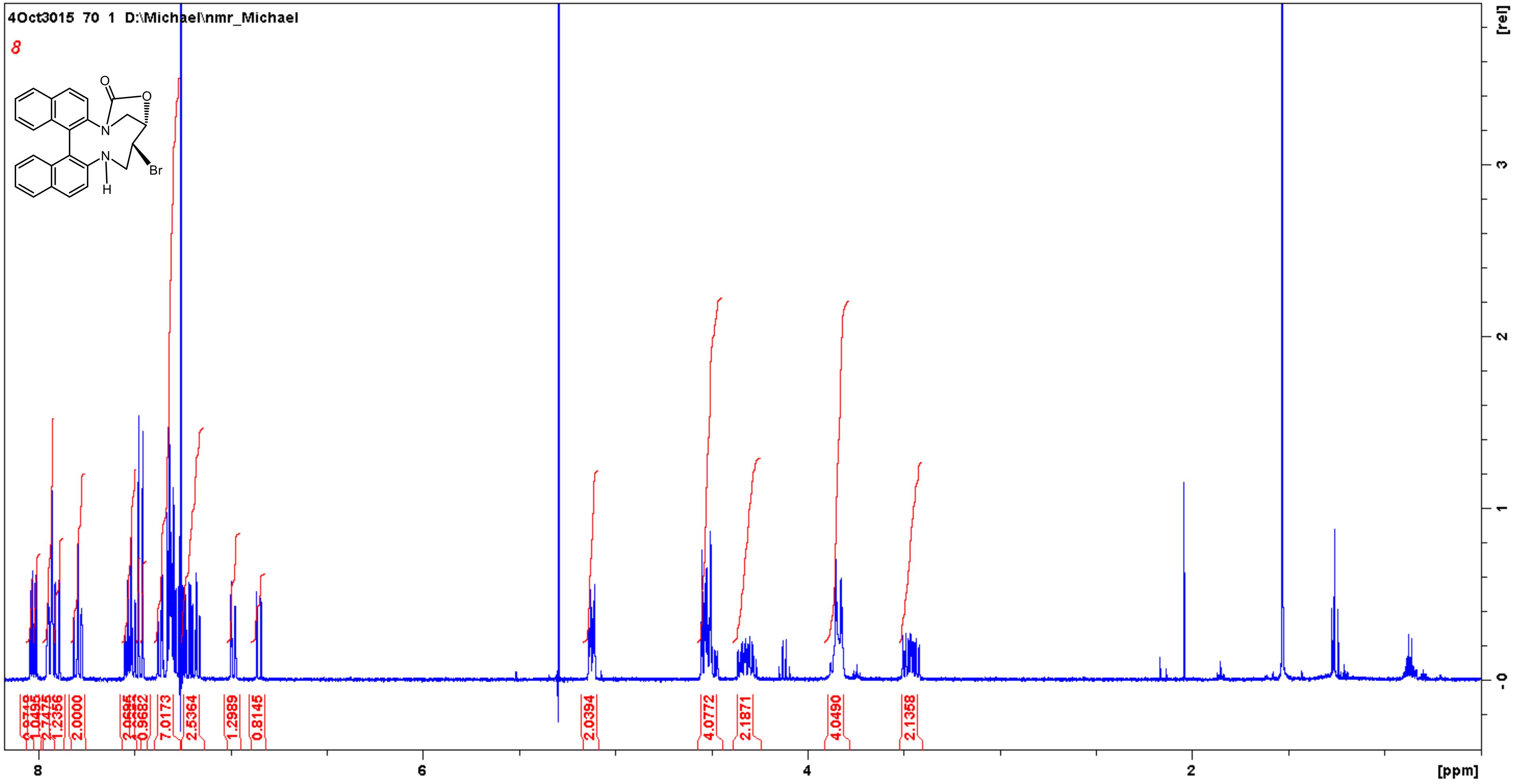

4Oct3015 70 1 D:\Michael\nmr_Michael
8
[rel]
2.0000
7.0173
2.5364
1.2989
0.8145
2.0394
4.0772
2.1871
4.0490
2.1358
8
6
4
2
[ppm]

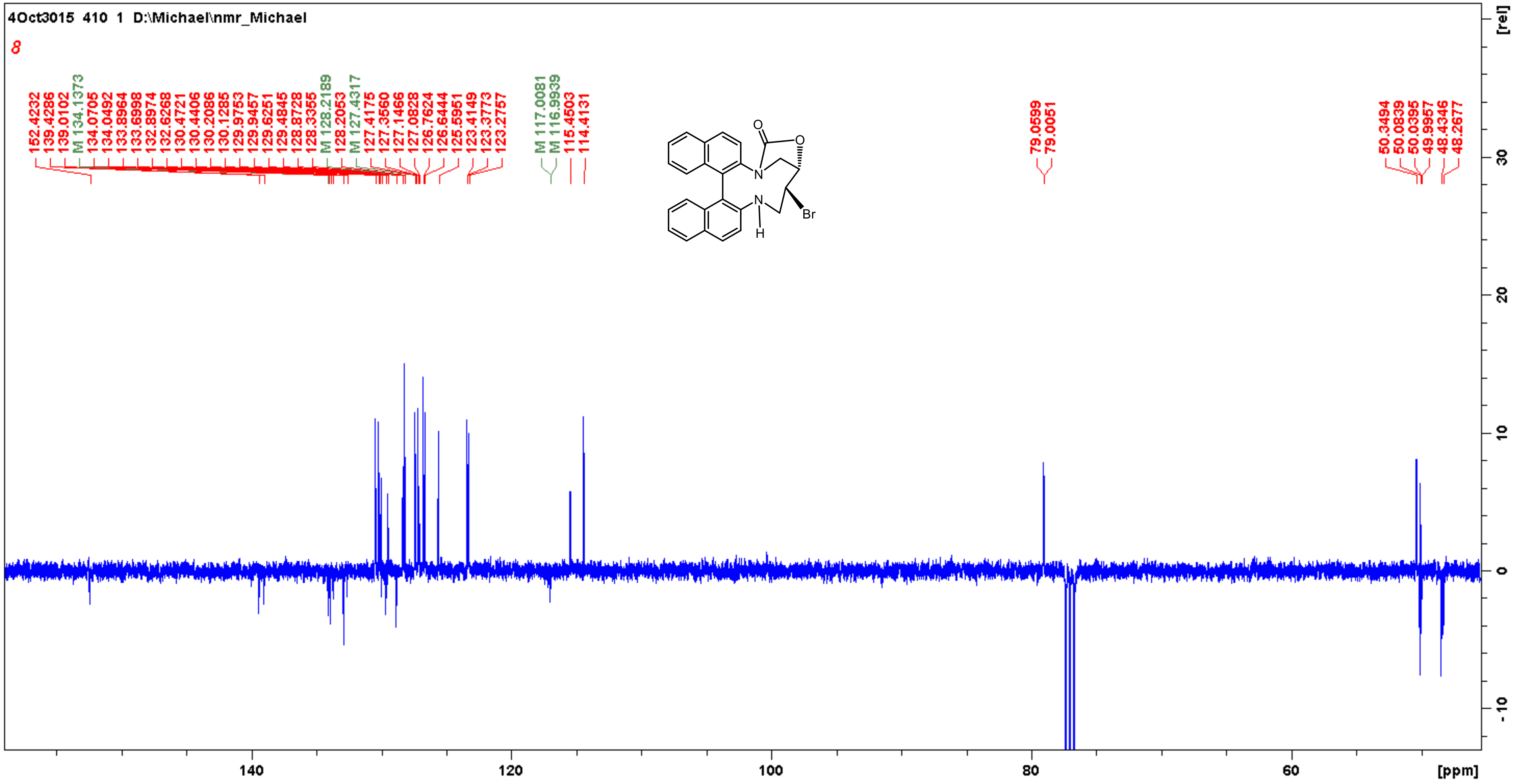

4Oct3015 410 1 D:\Michael\nmr_Michael
8
152.4232
139.4286
139.0102
M 134.1373
134.0705
134.0492
133.8964
133.6998
132.8974
132.6268
130.4721
130.4406
130.2086
130.1285
129.9753
129.9457
129.6251
129.4845
128.8728
128.3355
M 128.2189
128.2053
M 127.4317
127.4175
127.3560
127.1466
127.0828
126.7624
126.6444
125.5951
123.4149
123.3773
123.2757
M 117.0081
M 116.9939
115.4503
114.4131
79.0599
79.0051
50.3494
50.0839
50.0395
49.9957
48.4346
48.2677
O
O
N
N
H
Br
[rel]
30
20
10
0
-10
140
120
100
80
60
[ppm]

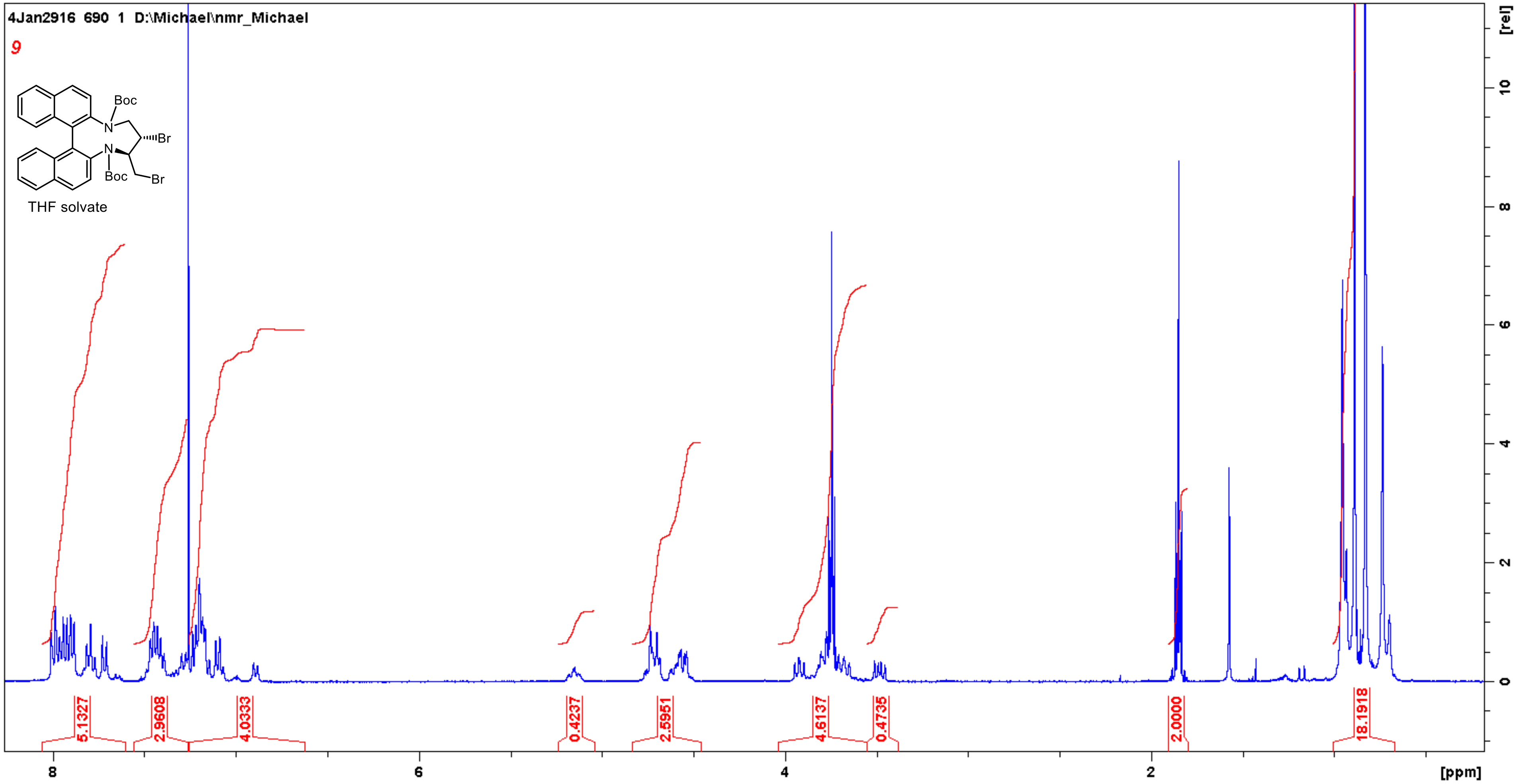

4Jan2916 690 1 D:\Michael\nmr_Michael
9
Boc
N
Br
N
Boc
Br
THF solvate
5.1327
2.9608
4.0333
0.4237
2.5951
4.6137
0.4735
2.0000
18.1918
8
6
4
2
[ppm]
[rel]
0
2
4
6
8
10

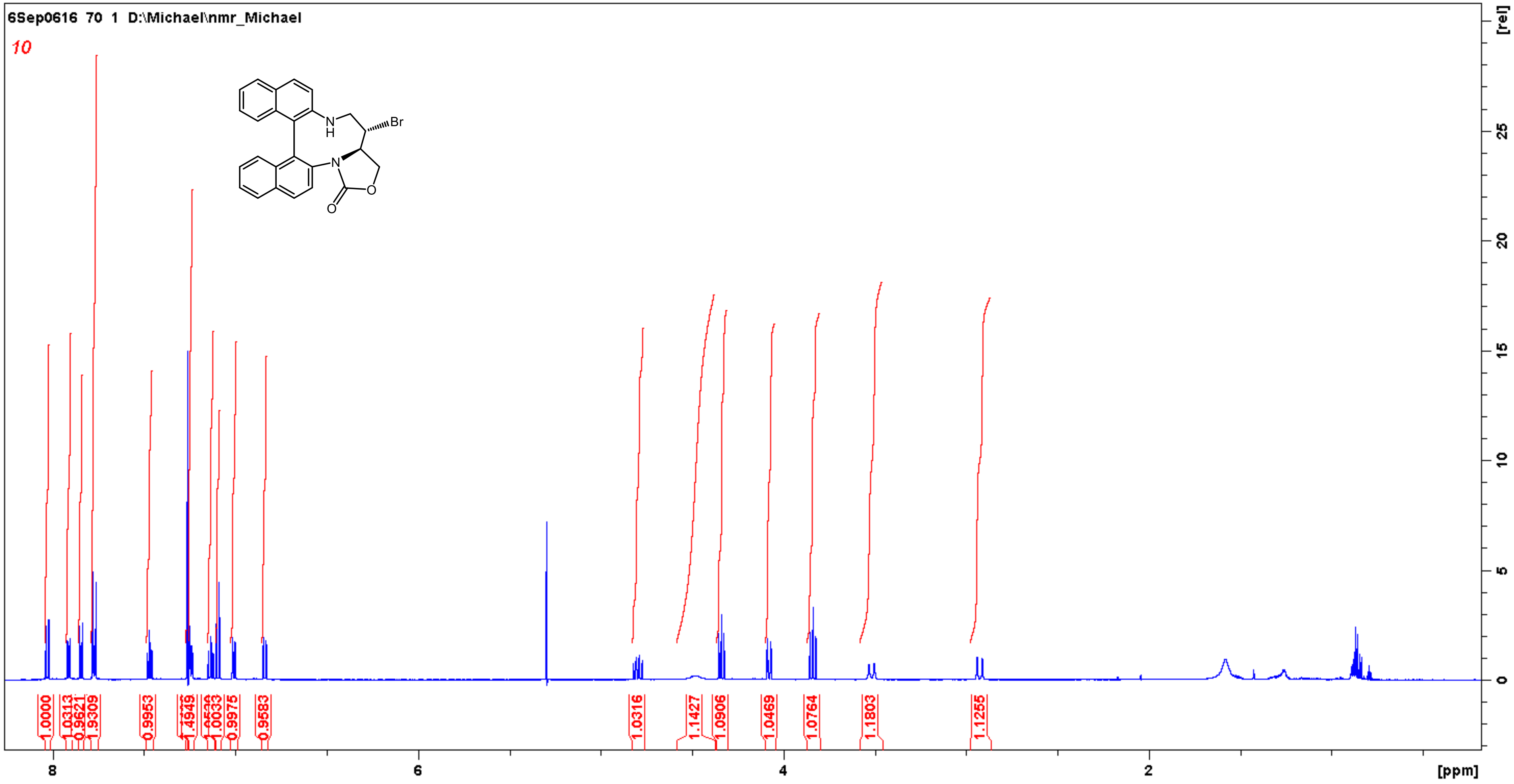
6Sep0616 70 1 D:\Michael\nmr_Michael
10
N
H
Br
N
O
O
[rel]
25
20
15
10
5
0
1.0000
1.0313
0.9621
1.9309
0.9953
1.4949
1.0033
0.9975
0.9583
1.0316
1.1427
1.0906
1.0469
1.0764
1.1803
1.1255
8
6
4
2
[ppm]

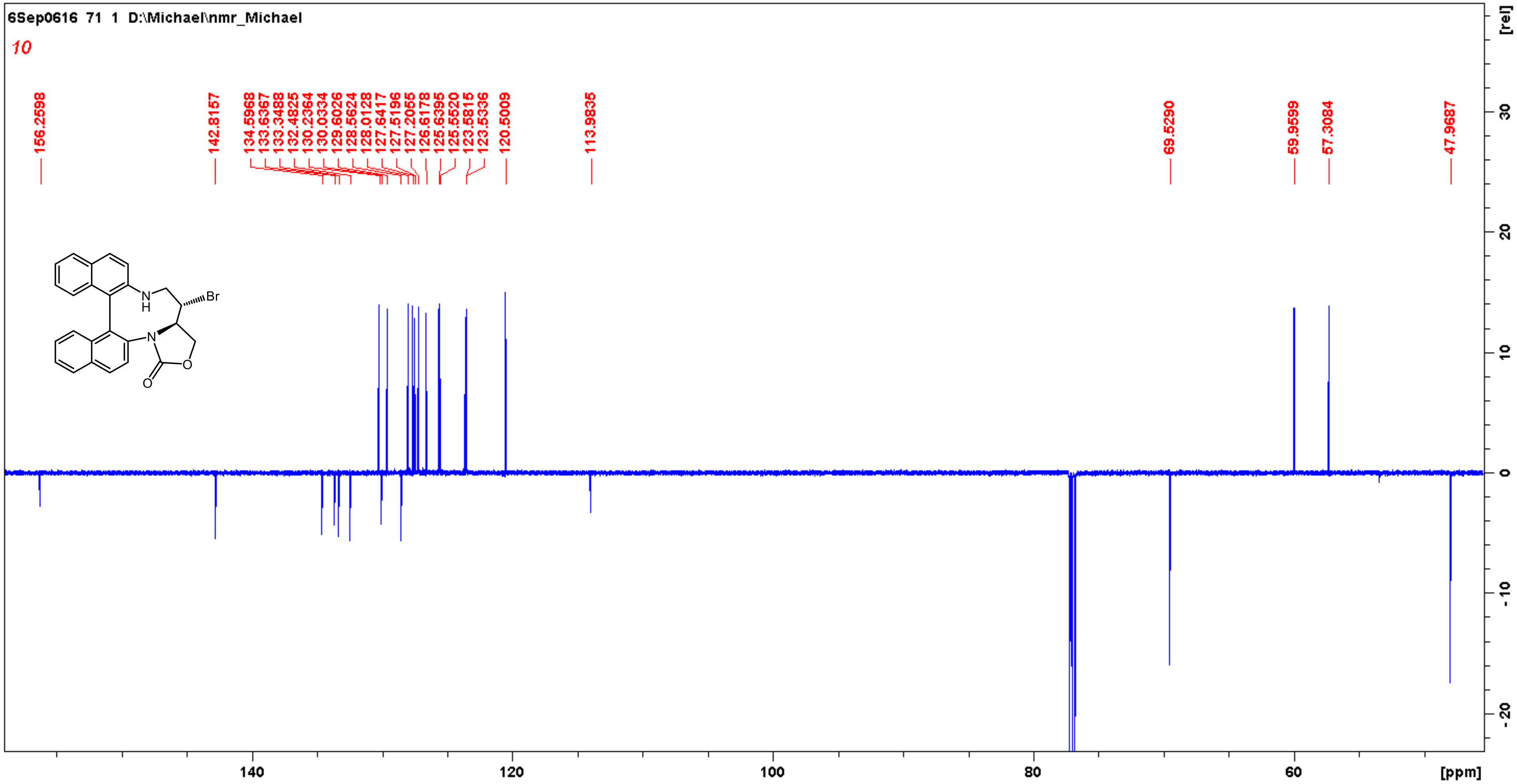
6Sep0616 71 1 D:\Michael\nmr_Michael
10
156.2598
142.8157
134.5968
133.6367
133.3488
132.4825
130.2364
130.0334
129.6026
128.5624
128.0128
127.6417
127.5196
127.2055
126.6178
125.6395
125.5520
123.5815
123.5336
120.5009
113.9835
69.5290
59.9599
57.3084
47.9687
[rel]
30
20
10
0
-10
-20
140
120
100
80
60
[ppm]
N
H
Br
N
O
O

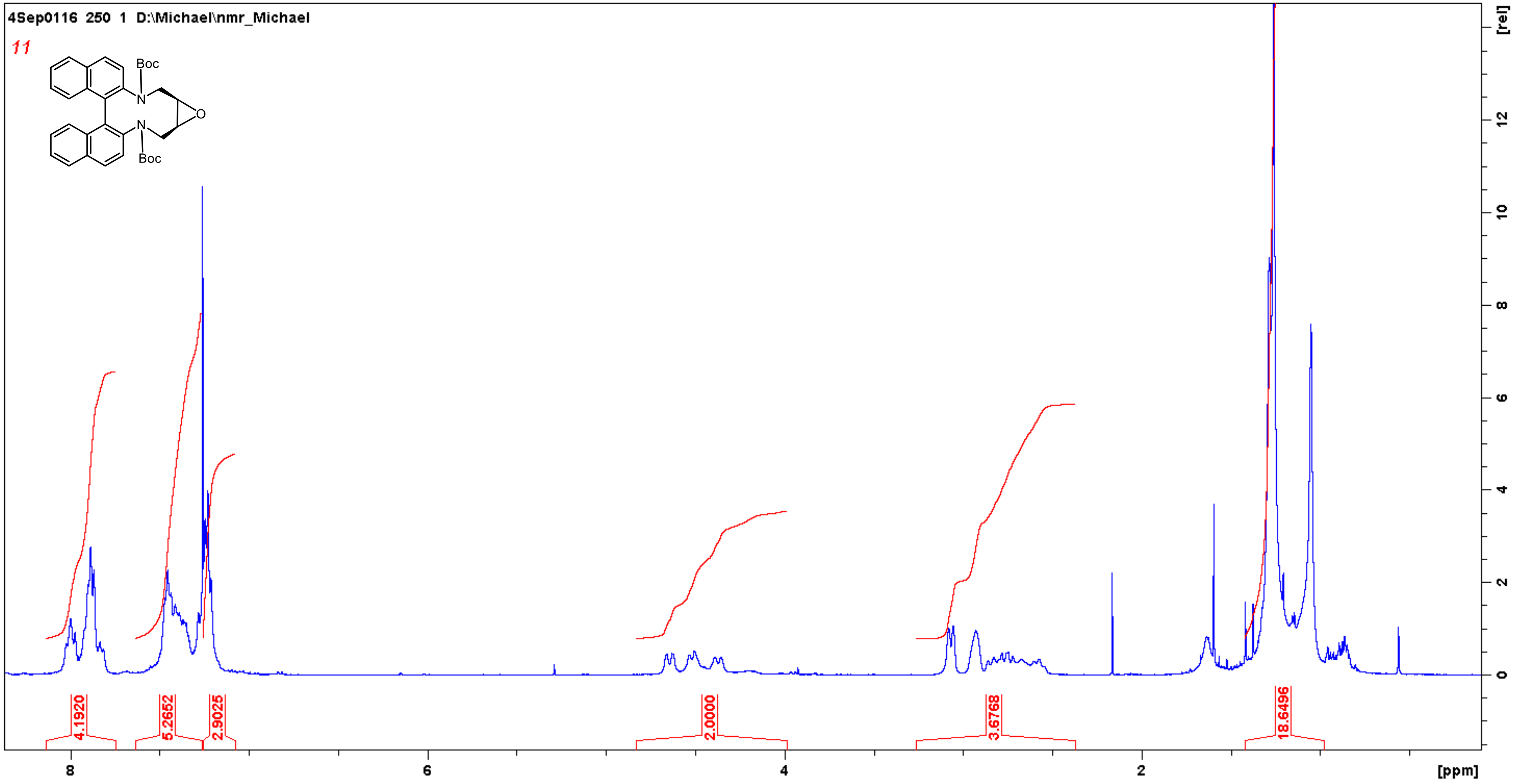
4Sep0116 250 1 D:\Michael\nmr_Michael
11
Boc
N
N
O
Boc
[rel]
12
10
8
6
4
2
0
4.1920
5.2652
2.9025
2.0000
3.6768
18.6496
8
6
4
2
[ppm]

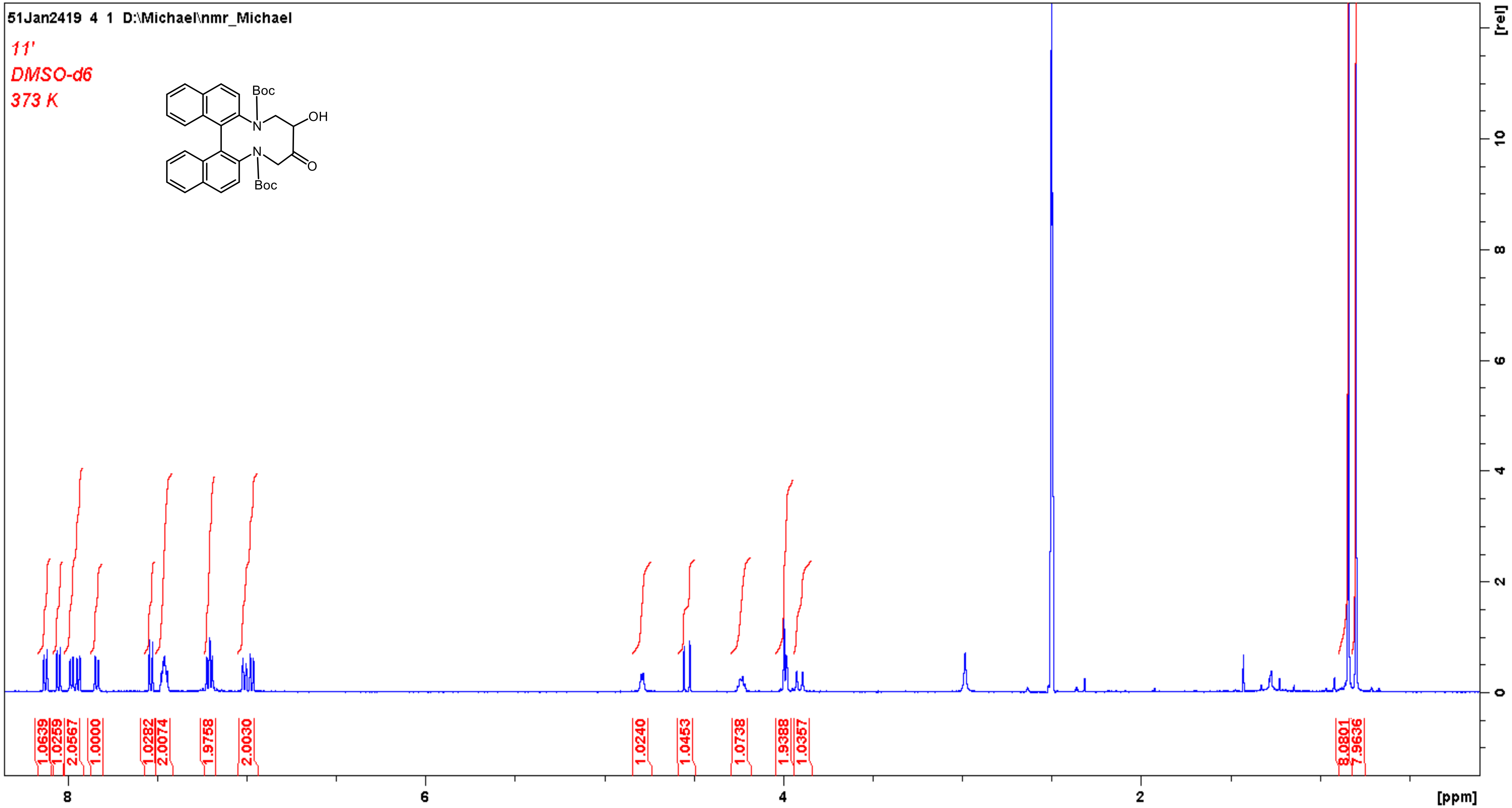
51Jan2419 4 1 D:\Michael\nmr_Michael
11'
DMSO-d6
373 K
Boc
OH
N
N
O
Boc
1.0639
1.0259
2.0567
1.0000
1.0282
2.0074
1.9758
2.0030
1.0240
1.0453
1.0738
1.9388
1.0357
8.0801
7.9636
8
6
4
2
[ppm]
[rel]
10
8
6
4
2
0

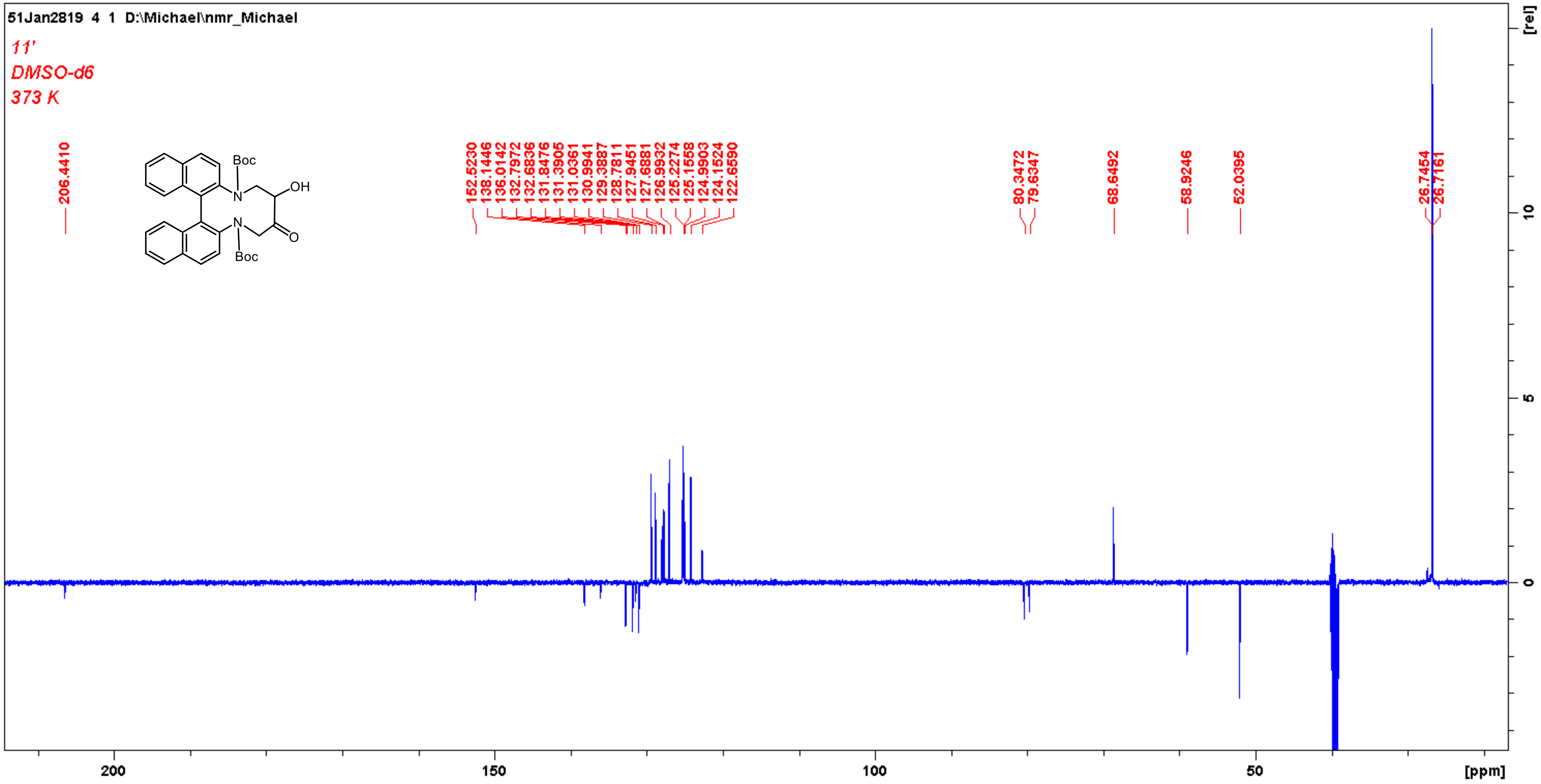

51Jan2819 4 1 D:\Michael\nmr_Michael
11'
DMSO-d6
373 K
Boc
OH
N
N
O
Boc
206.4410
152.5230
138.1446
136.0142
132.7972
132.6836
131.8476
131.3905
131.0361
130.9941
129.3887
128.7811
127.9451
127.6881
126.9932
125.2274
125.1558
124.9903
124.1524
122.6590
80.3472
79.6347
68.6492
58.9246
52.0395
26.7454
26.7161
[rel]
10
5
0
200
150
100
50
[ppm]

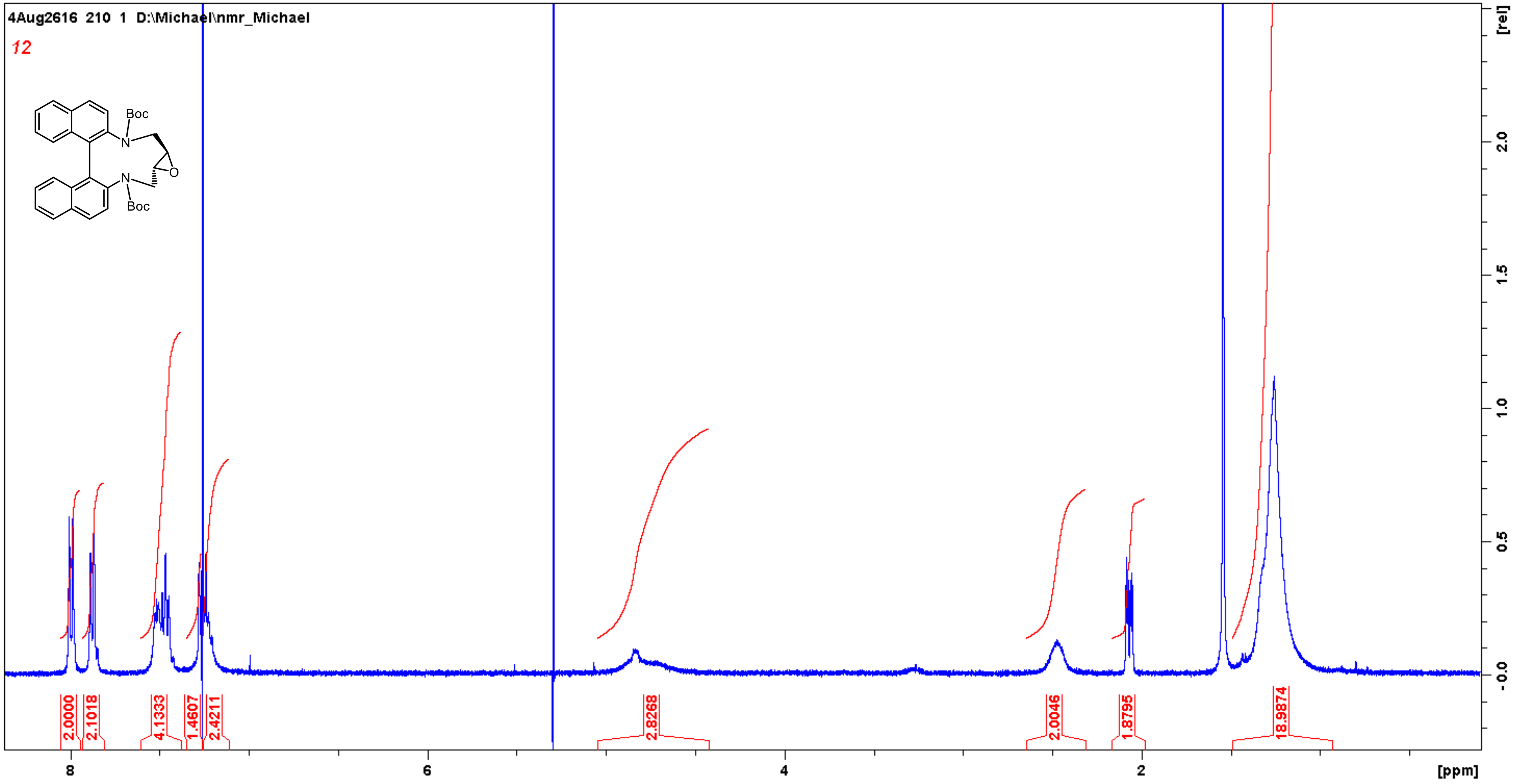
4Aug2616 210 1 D:\Michael\nmr_Michael
12
Boc
N
O
N
Boc
2.0000
2.1018
4.1333
1.4607
2.4211
2.8268
2.0046
1.8795
18.9874
8
6
4
2
[ppm]
[rel]
2.0
1.5
1.0
0.5
-0.0

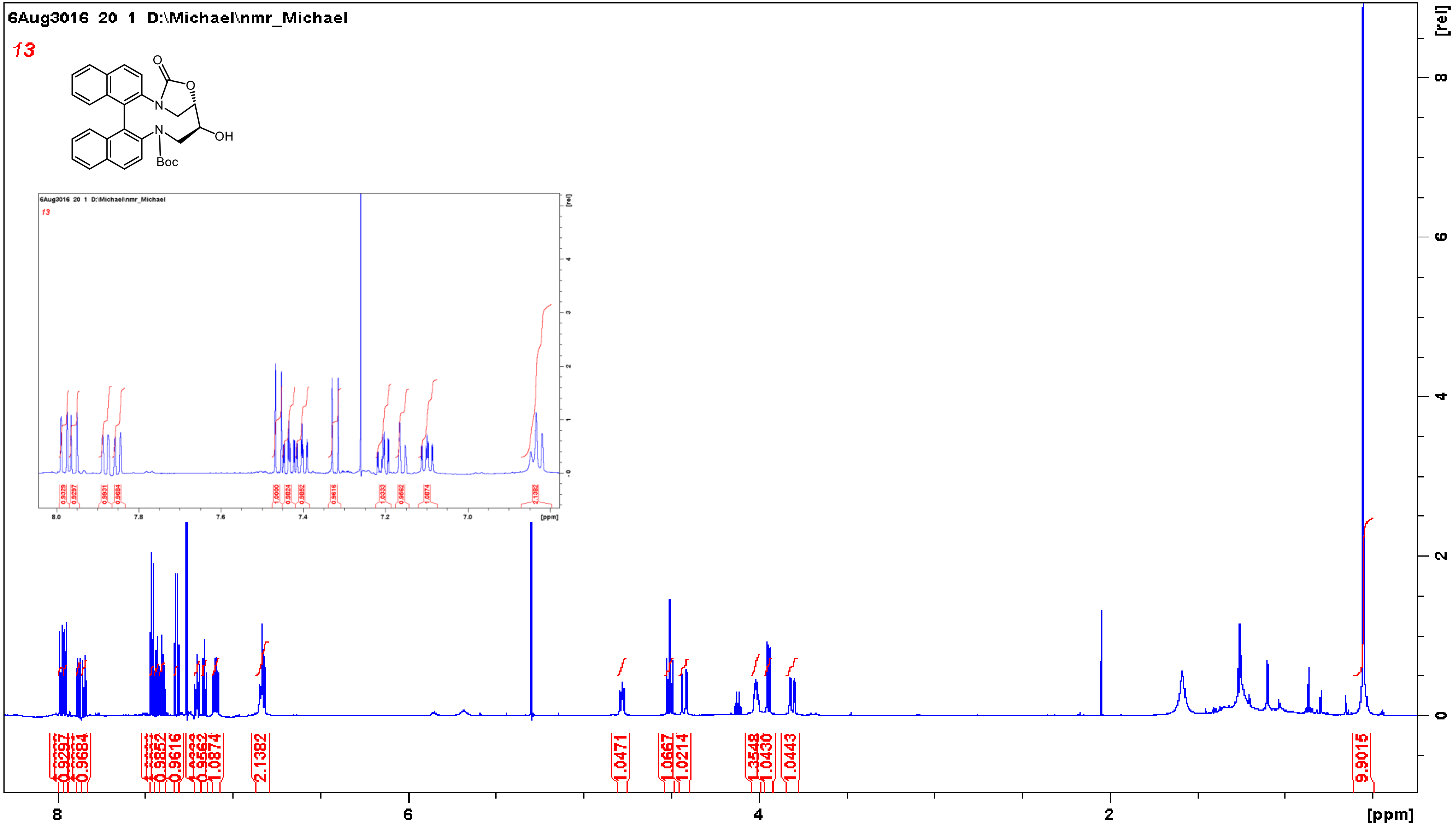
6Aug3016 20 1 D:\Michael\nmr_Michael
13
O
O
N
N
OH
Boc
[rel]
[ppm]
8
6
4
2
0
0.9297
0.9684
0.9852
0.9616
0.9562
1.0874
2.1382
1.0471
1.0667
1.0214
1.3548
1.0430
1.0443
9.9015

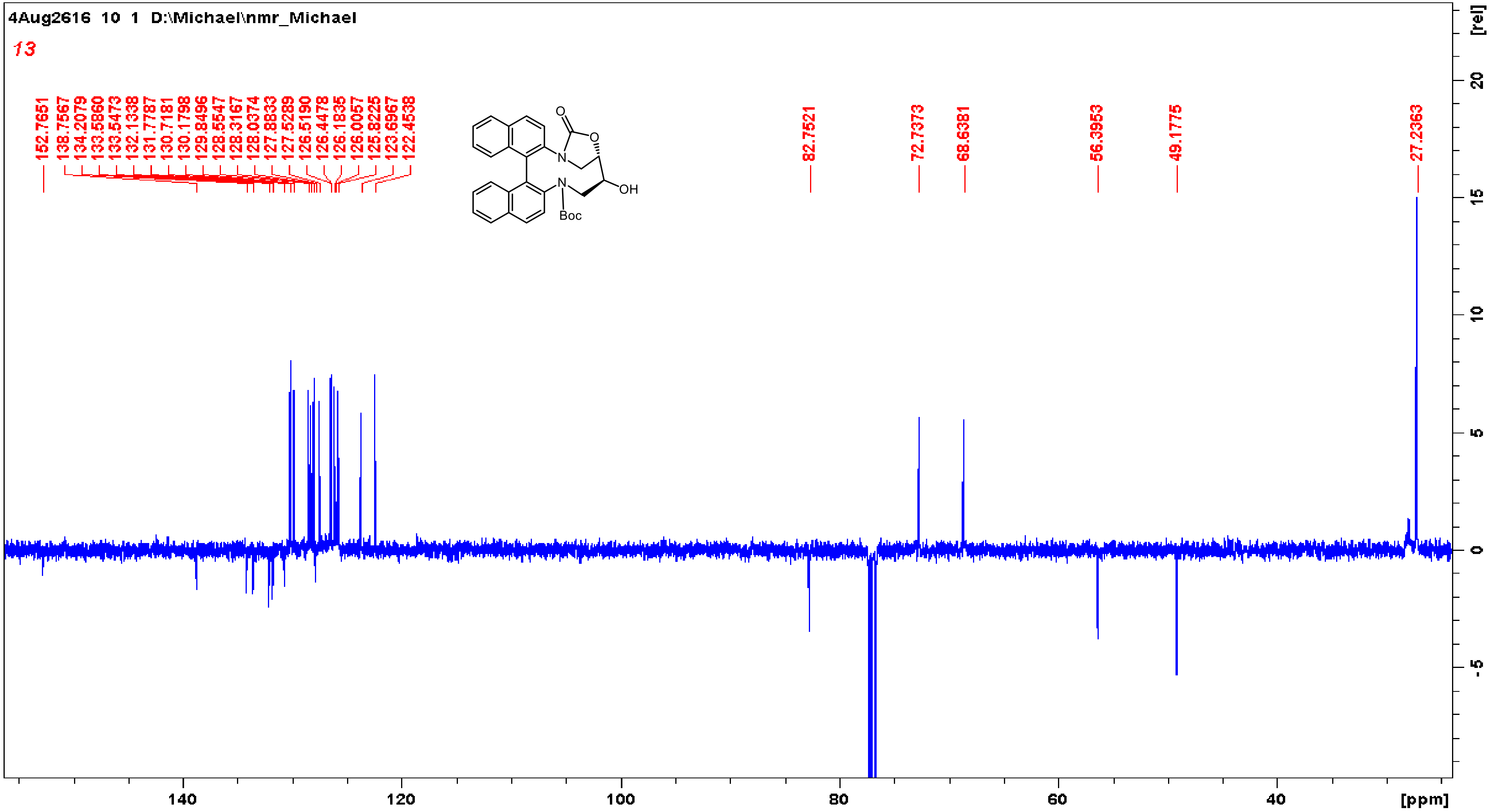
4Aug2616 10 1 D:\Michael\nmr_Michael
13
152.7651
138.7567
134.2079
133.5860
133.5473
132.1338
131.7787
130.7181
130.1798
129.8496
128.5547
128.3167
128.0374
127.8833
127.5289
126.5190
126.4478
126.1835
126.0057
125.8225
123.6967
122.4538
82.7521
72.7373
68.6381
56.3953
49.1775
27.2363
O
O
N
N
OH
Boc
[rel]
20
15
10
5
0
-5
140
120
100
80
60
40
[ppm]

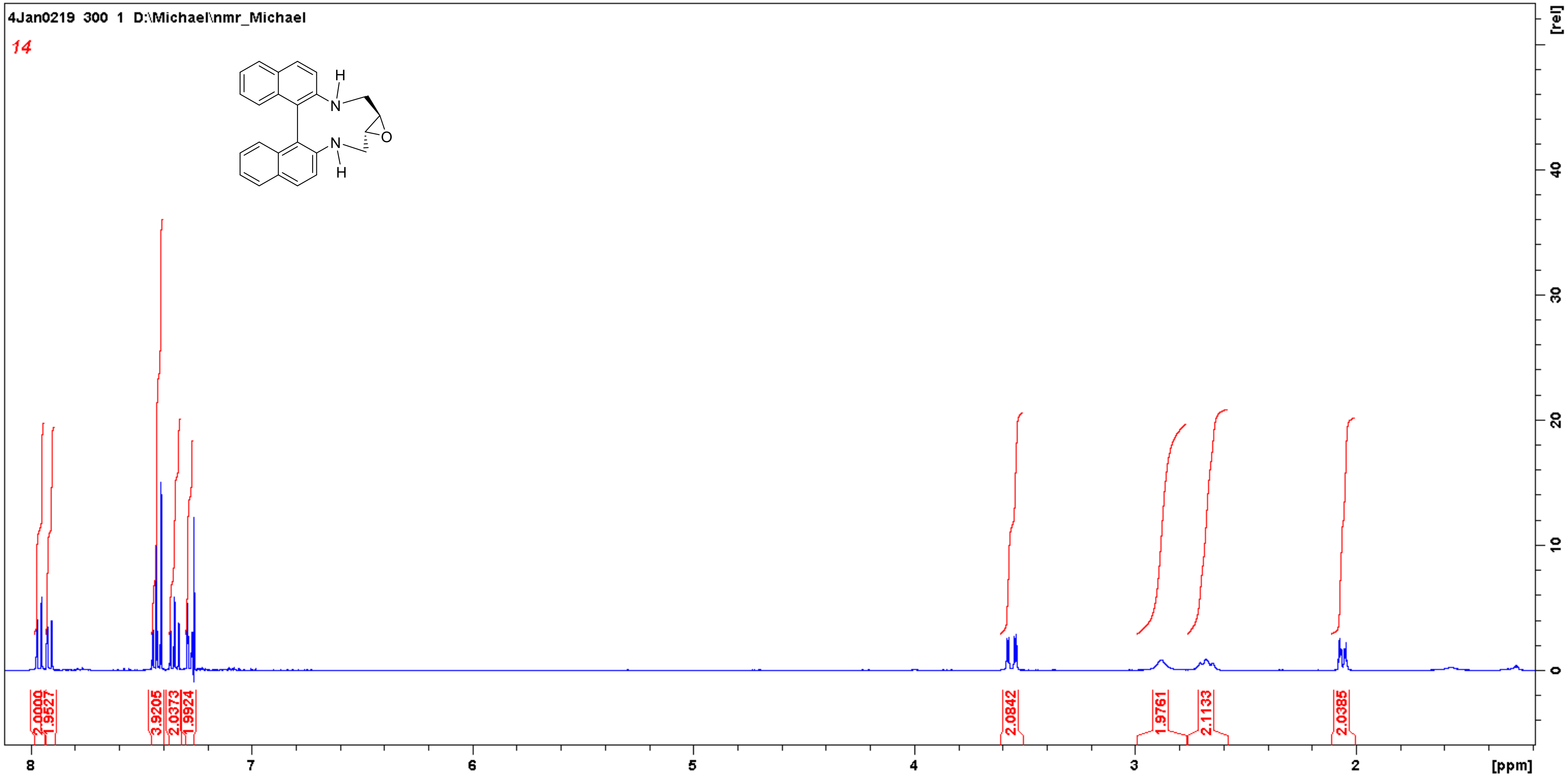
4Jan0219 300 1 D:\Michael\nmr_Michael
14
H
N
O
N
H
2.0000
1.9527
3.9205
2.0373
1.9924
2.0842
1.9761
2.1133
2.0385
[ppm]
[rel]

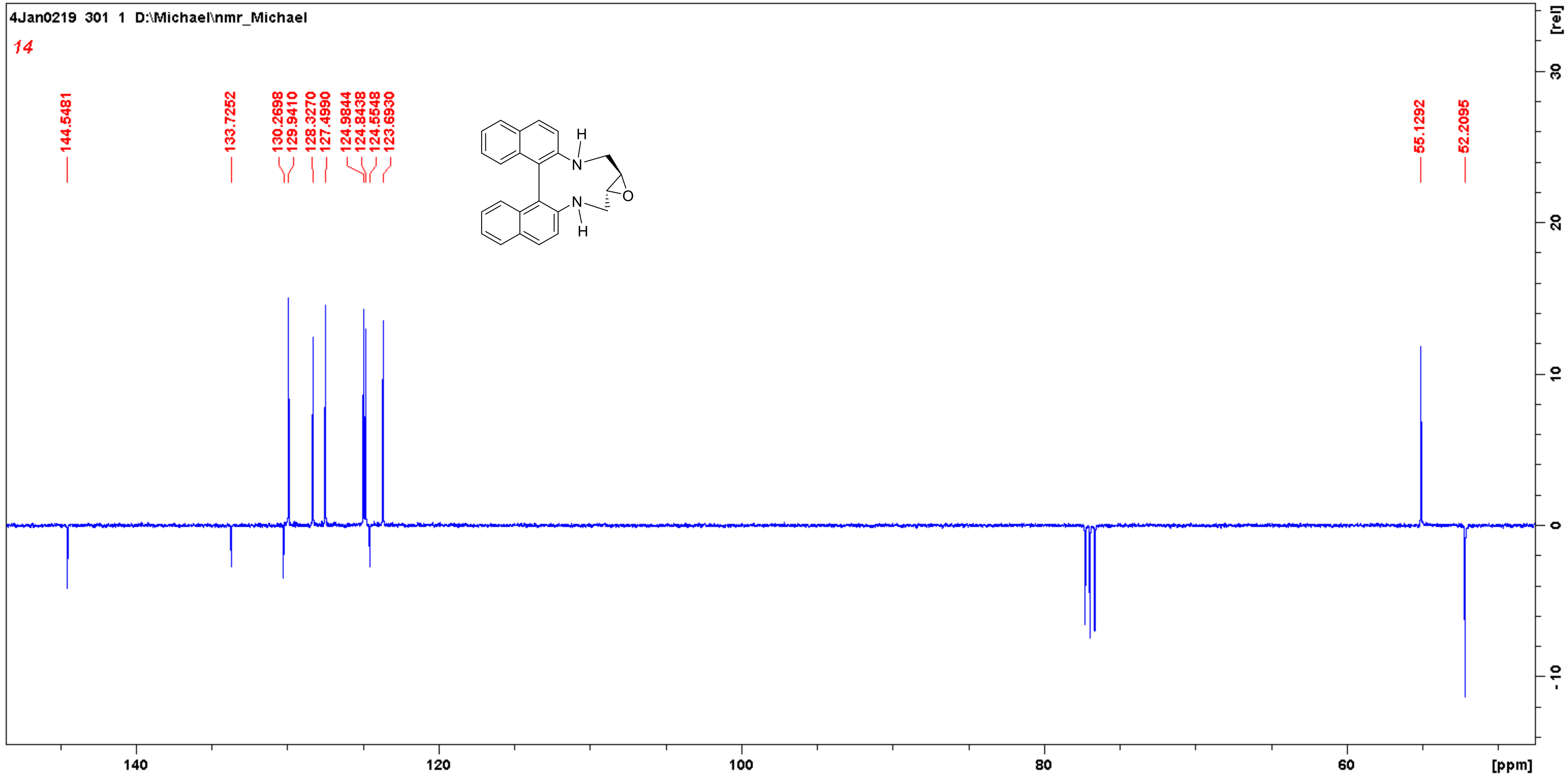
4Jan0219 301 1 D:\Michael\nmr_Michael
14
144.5481
133.7252
130.2698
129.9410
128.3270
127.4990
124.9844
124.8438
124.5548
123.6930
55.1292
52.2095
[rel]
[ppm]

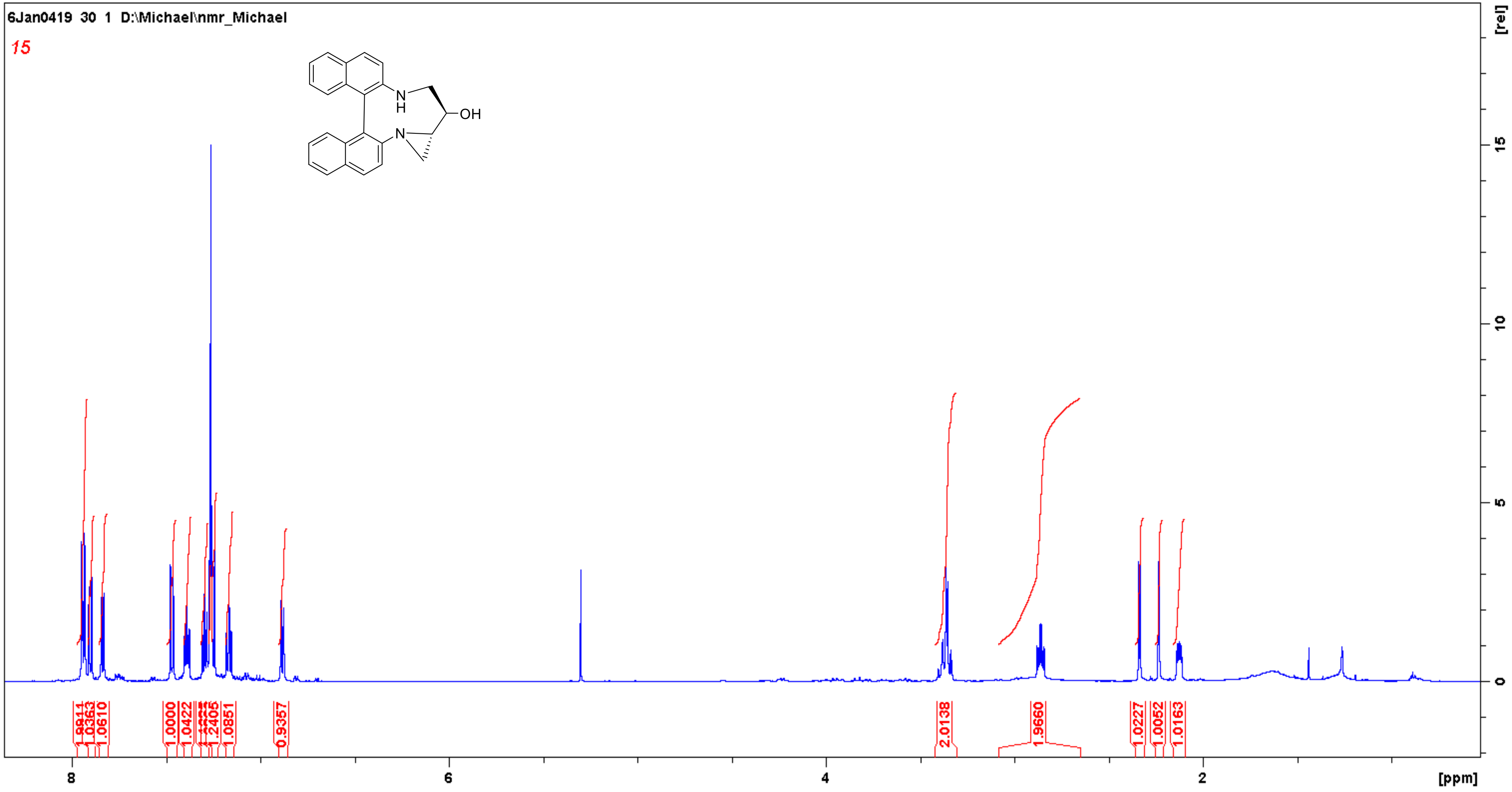
6Jan0419 30 1 D:\Michael\nmr_Michael
15
N
H
OH
N
[rel]
15
10
5
0
1.0363
1.0610
1.0000
1.0422
1.2405
1.0851
0.9357
2.0138
1.9660
1.0227
1.0052
1.0163
8
6
4
2
[ppm]

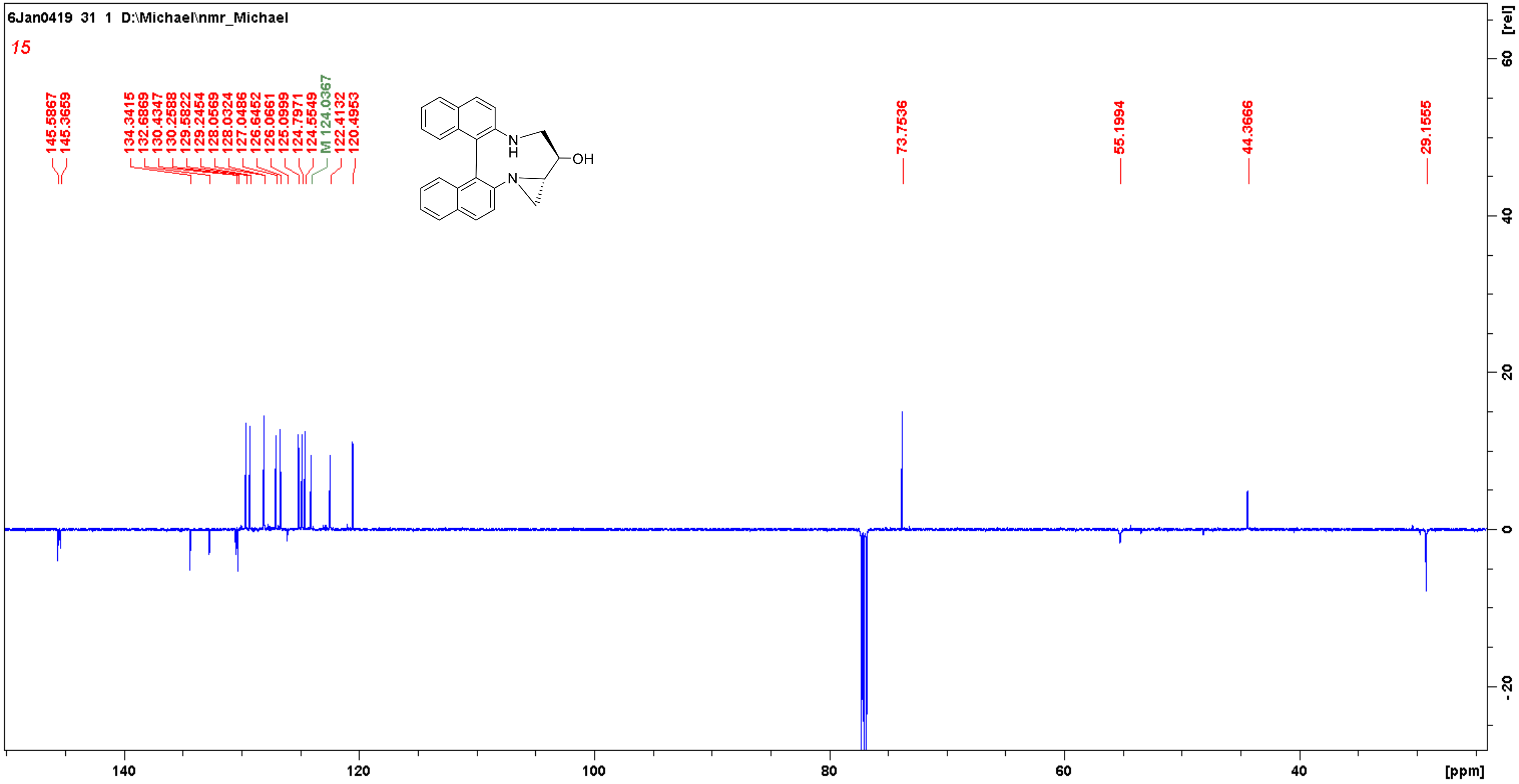
6Jan0419 31 1 D:\Michael\nmr_Michael
15
145.5867
145.3659
134.3415
132.6869
130.4347
130.2588
129.5822
129.2454
128.0569
128.0324
127.0486
126.6452
126.0661
125.0999
124.7971
124.5549
M 124.0367
122.4132
120.4953
73.7536
55.1994
44.3666
29.1555
N
H
OH
N
[rel]
60
40
20
0
-20
140
120
100
80
60
40
[ppm]

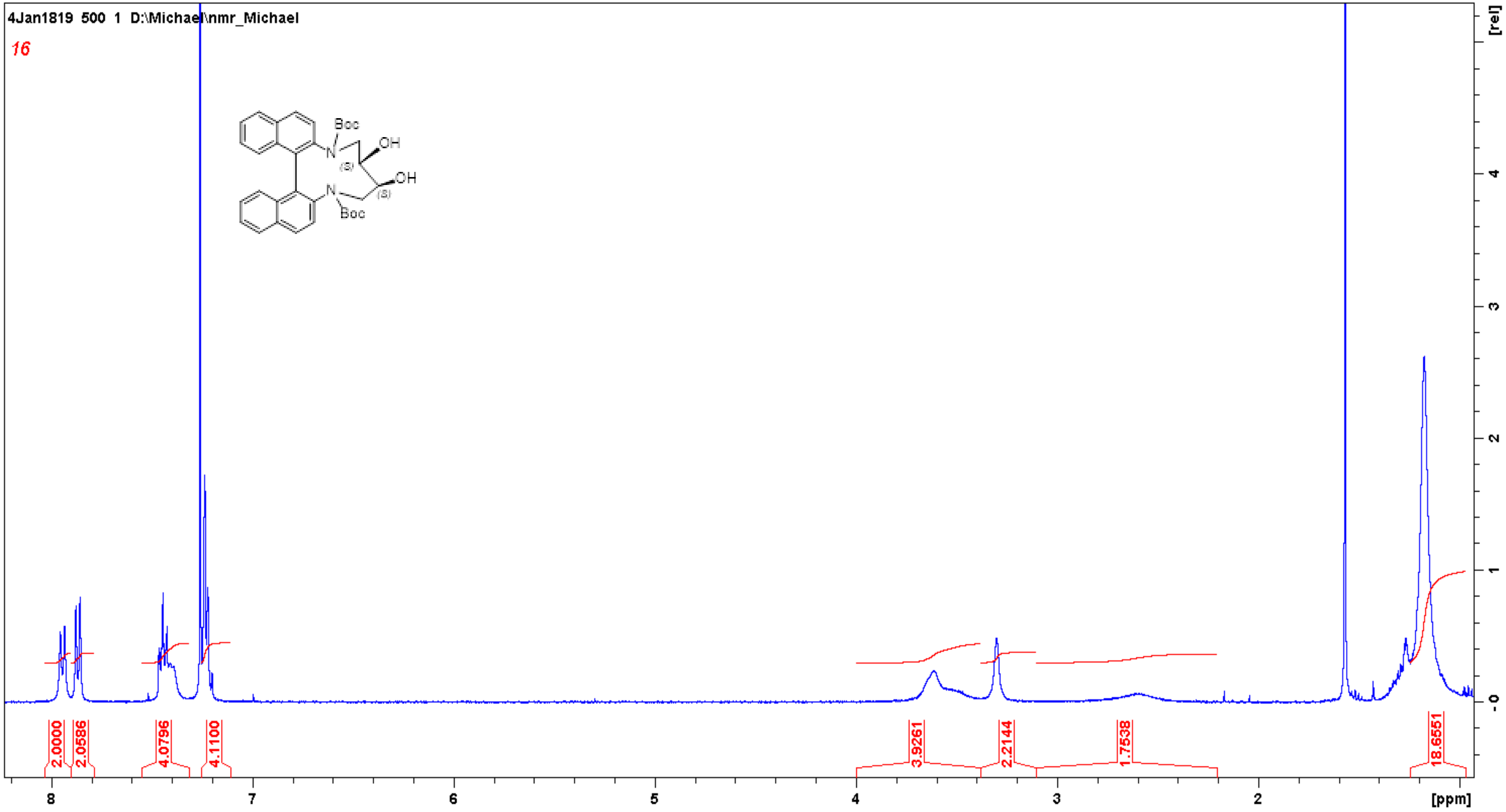
4Jan1819 500 1 D:\Michael\nmr_Michael
16
Boc
OH
OH
N
N
(S)
(S)
Boc
2.0000
2.0586
4.0796
4.1100
3.9261
2.2144
1.7538
18.6551
[rel]
[ppm]

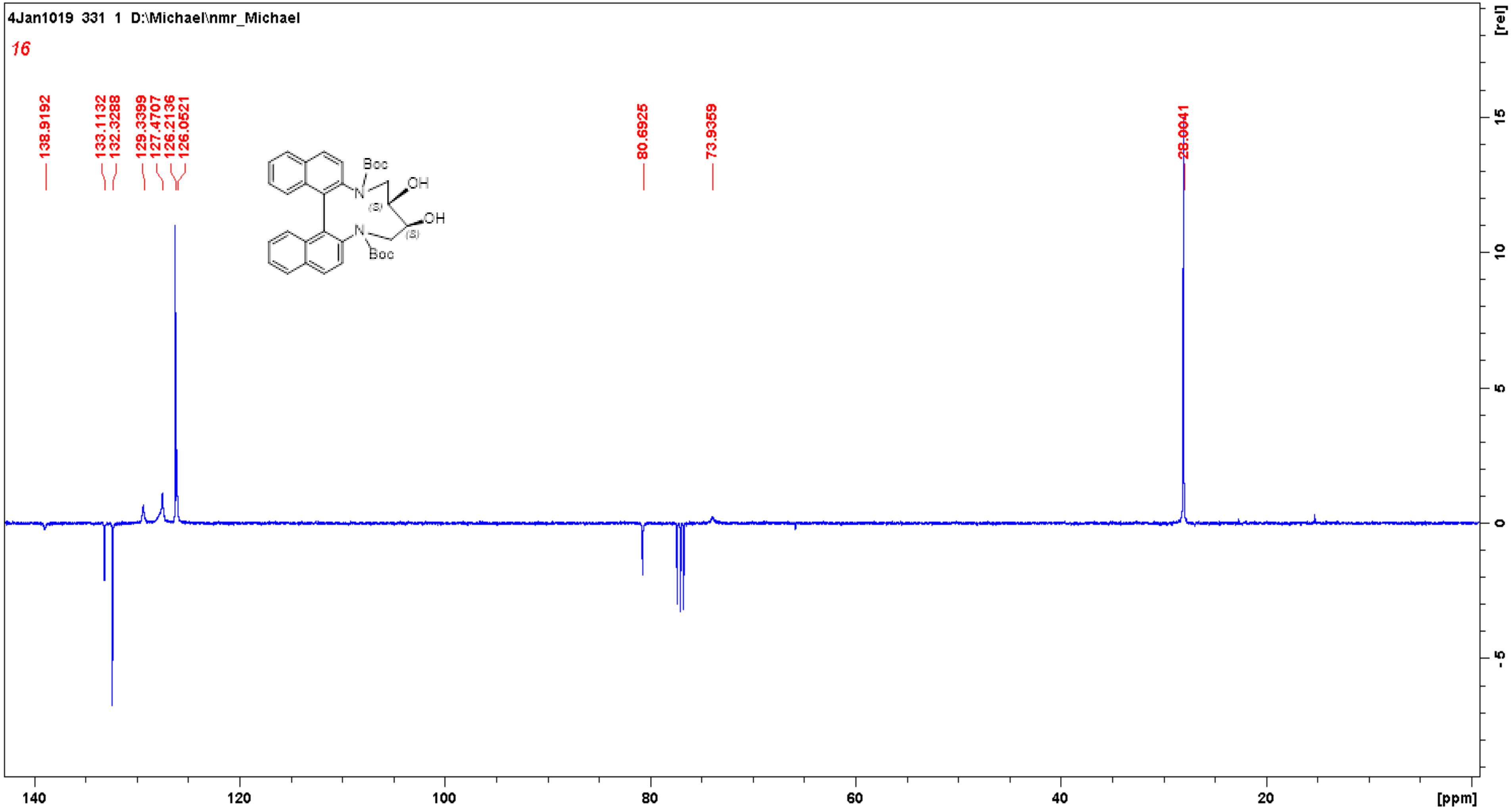
4Jan1019 331 1 D:\Michael\nmr_Michael
16
138.9192
133.1132
132.3288
129.3399
127.4707
126.2136
126.0521
80.6925
73.9359
28.0041
Boc
OH
OH
N
N
Boc
(S)
(S)
[rel]
[ppm]

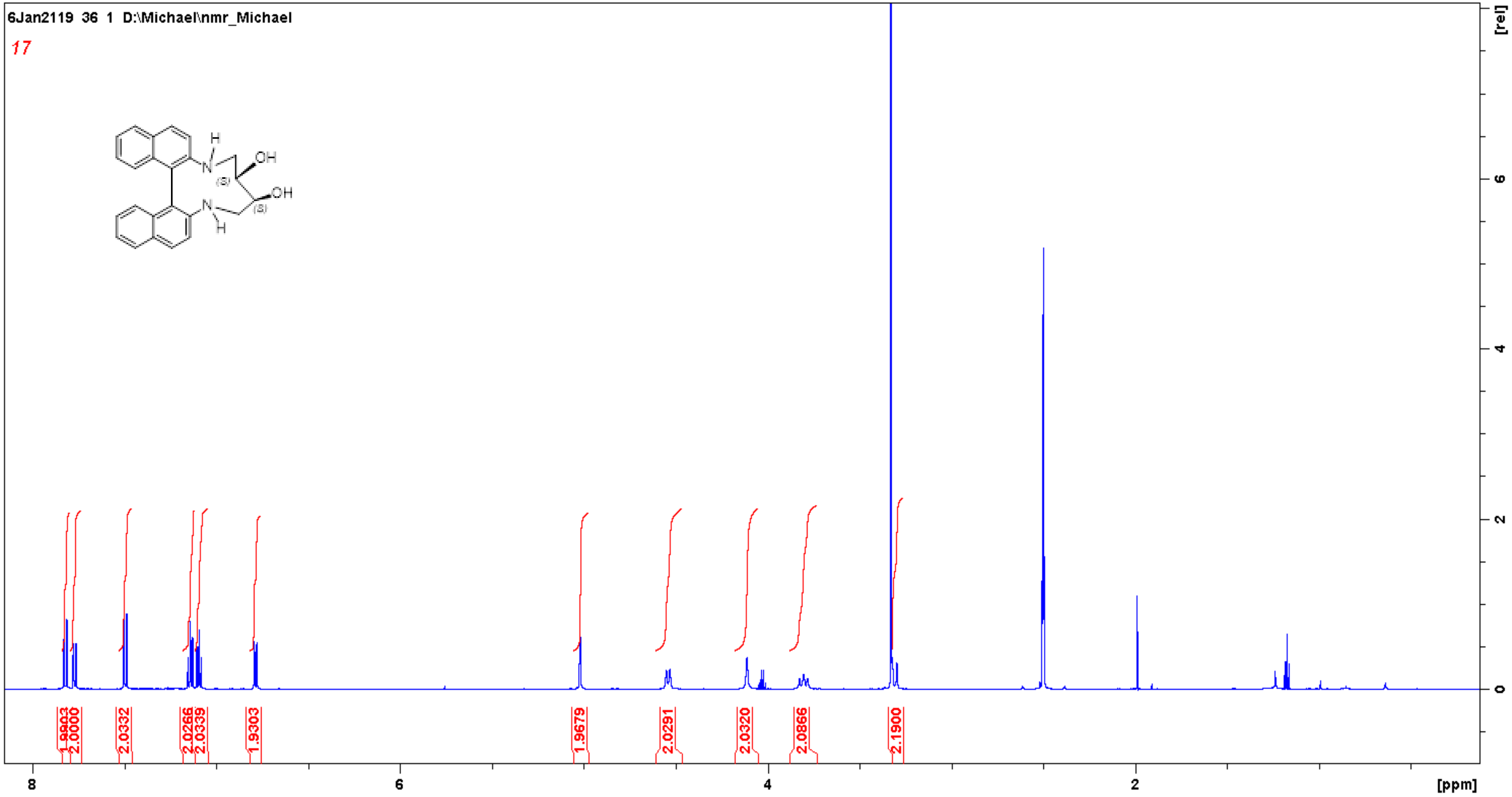
6Jan2119 36 1 D:\Michael\nmr_Michael
17
1.9903
2.0000
2.0332
2.0266
2.0339
1.9303
1.9679
2.0291
2.0320
2.0866
2.1900
[rel]
[ppm]
8
6
4
2
0

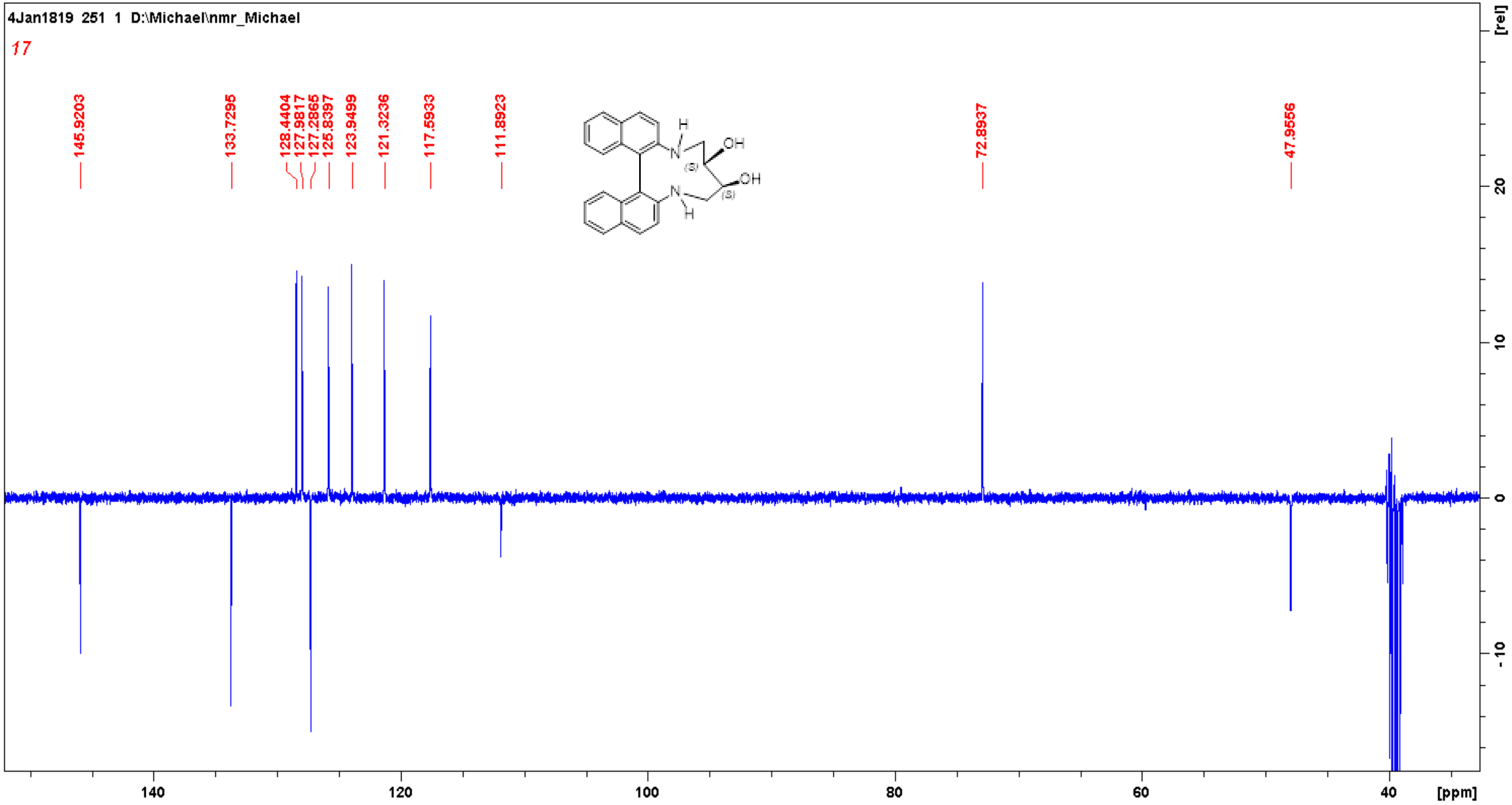
4Jan1819 251 1 D:\Michael\nmr_Michael
17
145.9203
133.7295
128.4404
127.9817
127.2865
125.8397
123.9499
121.3236
117.5933
111.8923
72.8937
47.9556
H
OH
(S)
OH
N
(S)
N
H
140
120
100
80
60
40
[ppm]
[rel]
20
10
0
-10

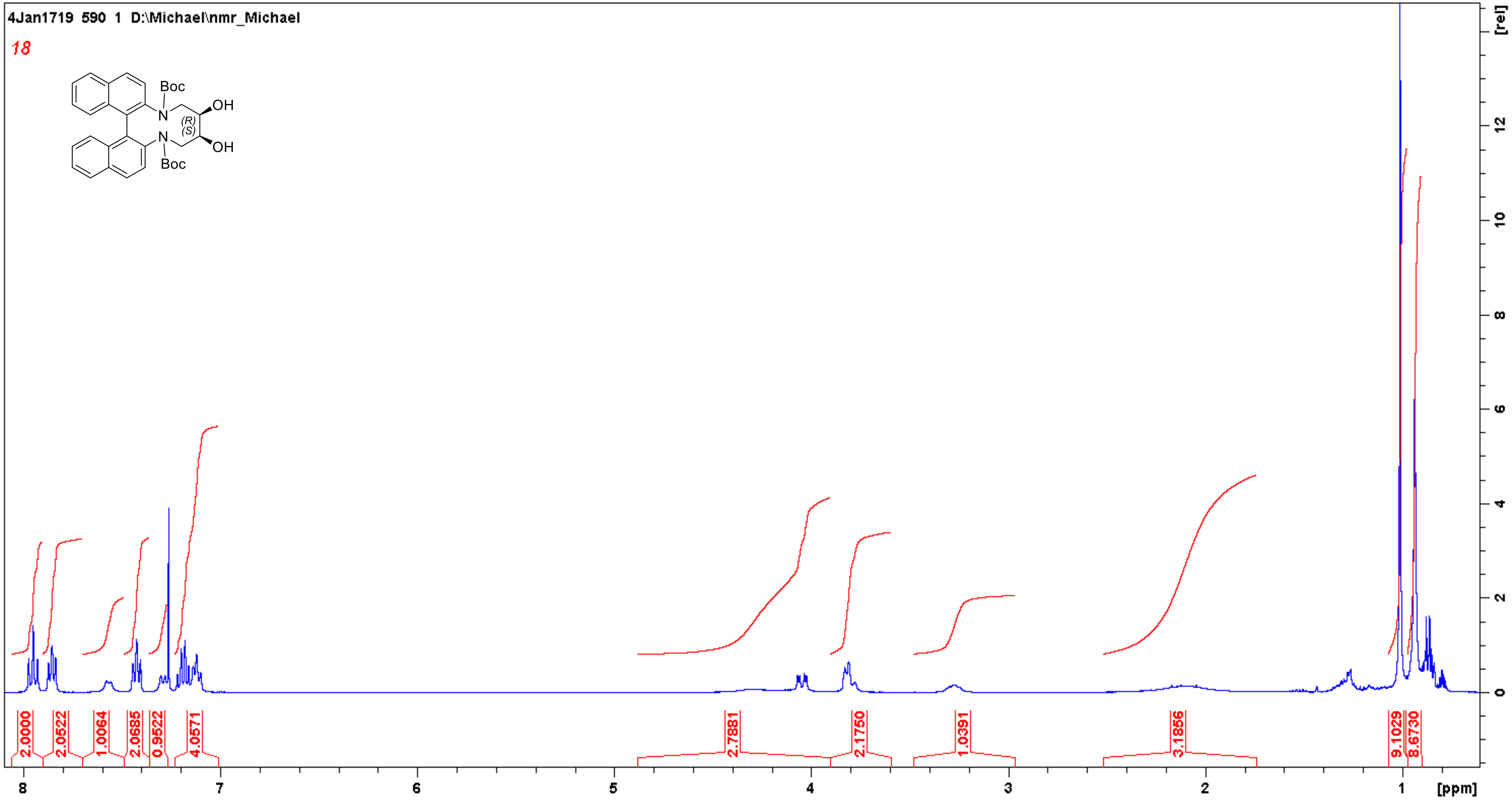
4Jan1719 590 1 D:\Michael\nmr_Michael
18
Boc
N
(R)
(S)
OH
N
OH
Boc
2.0000
2.0522
1.0064
2.0685
0.9522
4.0571
2.7881
2.1750
1.0391
3.1856
9.1029
8.6730
8
7
6
5
4
3
2
1
[ppm]
[rel]
12
10
8
6
4
2
0

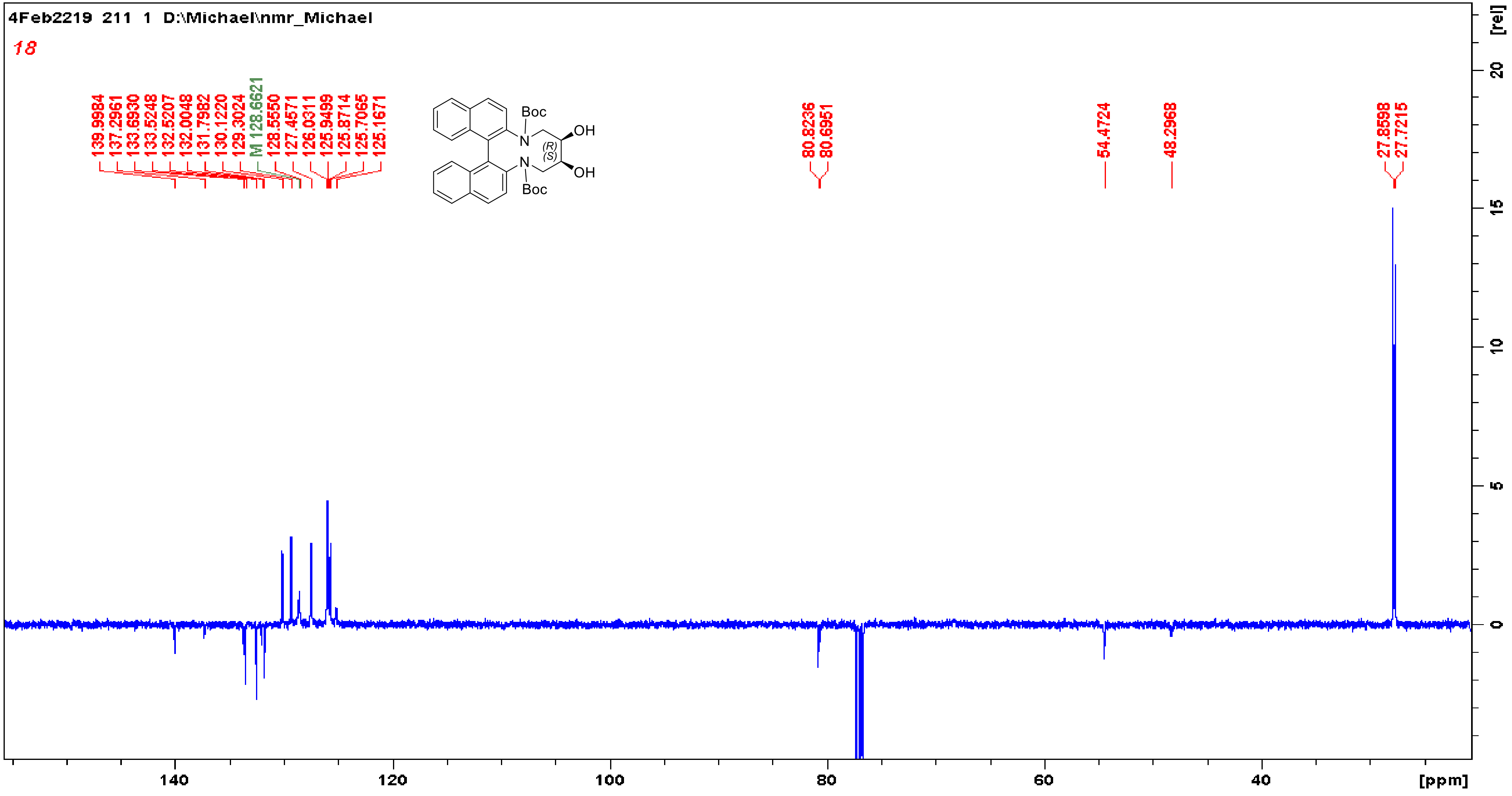
4Feb2219 211 1 D:\Michael\nmr_Michael
18
139.9984
137.2961
133.6930
133.5248
132.5207
132.0048
131.7982
130.1220
129.3024
M 128.6621
128.5550
127.4571
126.0311
125.9499
125.8714
125.7065
125.1671
80.8236
80.6951
54.4724
48.2968
27.8598
27.7215
Boc
N
(R)
(S)
OH
OH
N
Boc
[rel]
20
15
10
5
0
140
120
100
80
60
40
[ppm]

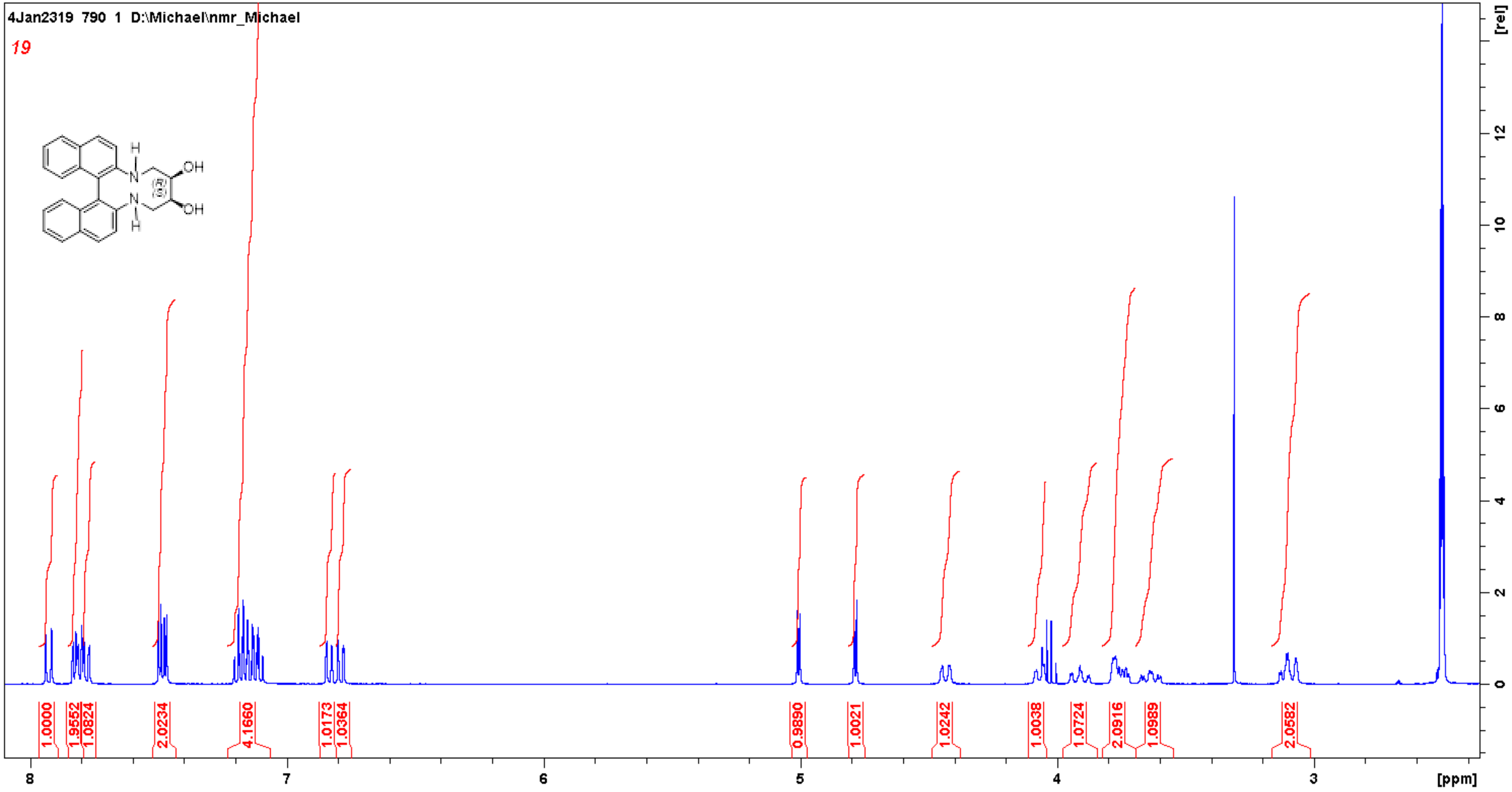
4Jan2319 790 1 D:\Michael\nmr_Michael
19
H
N
OH
(S)
(S)
OH
N
H
1.0000
1.9552
1.0824
2.0234
4.1660
1.0173
1.0364
0.9890
1.0021
1.0242
1.0038
1.0724
2.0916
1.0989
2.0582
8
7
6
5
4
3
[ppm]
[rel]
12
10
8
6
4
2
0

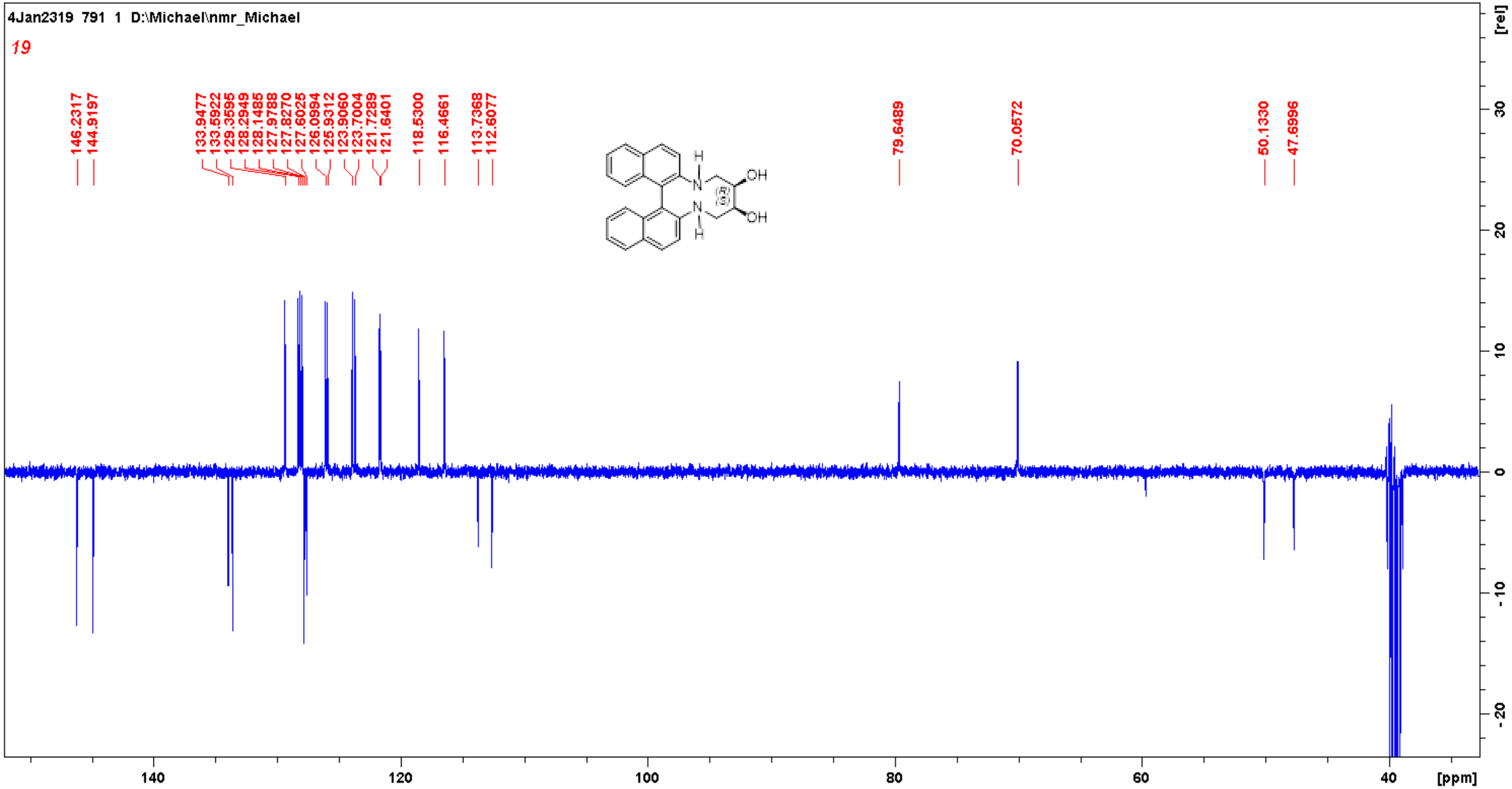
4Jan2319 791 1 D:\Michael\nmr_Michael
19
146.2317
144.9197
133.9477
133.5922
129.3595
128.2949
128.1485
127.9788
127.8270
127.6025
126.0994
125.9312
123.9060
123.7004
121.7289
121.6401
118.5300
116.4661
113.7368
112.6077
79.6489
70.0572
50.1330
47.6996
H
N
N
H
OH
OH
(R)
(S)
140
120
100
80
60
40
[ppm]
[rel]
30
20
10
0
-10
-20

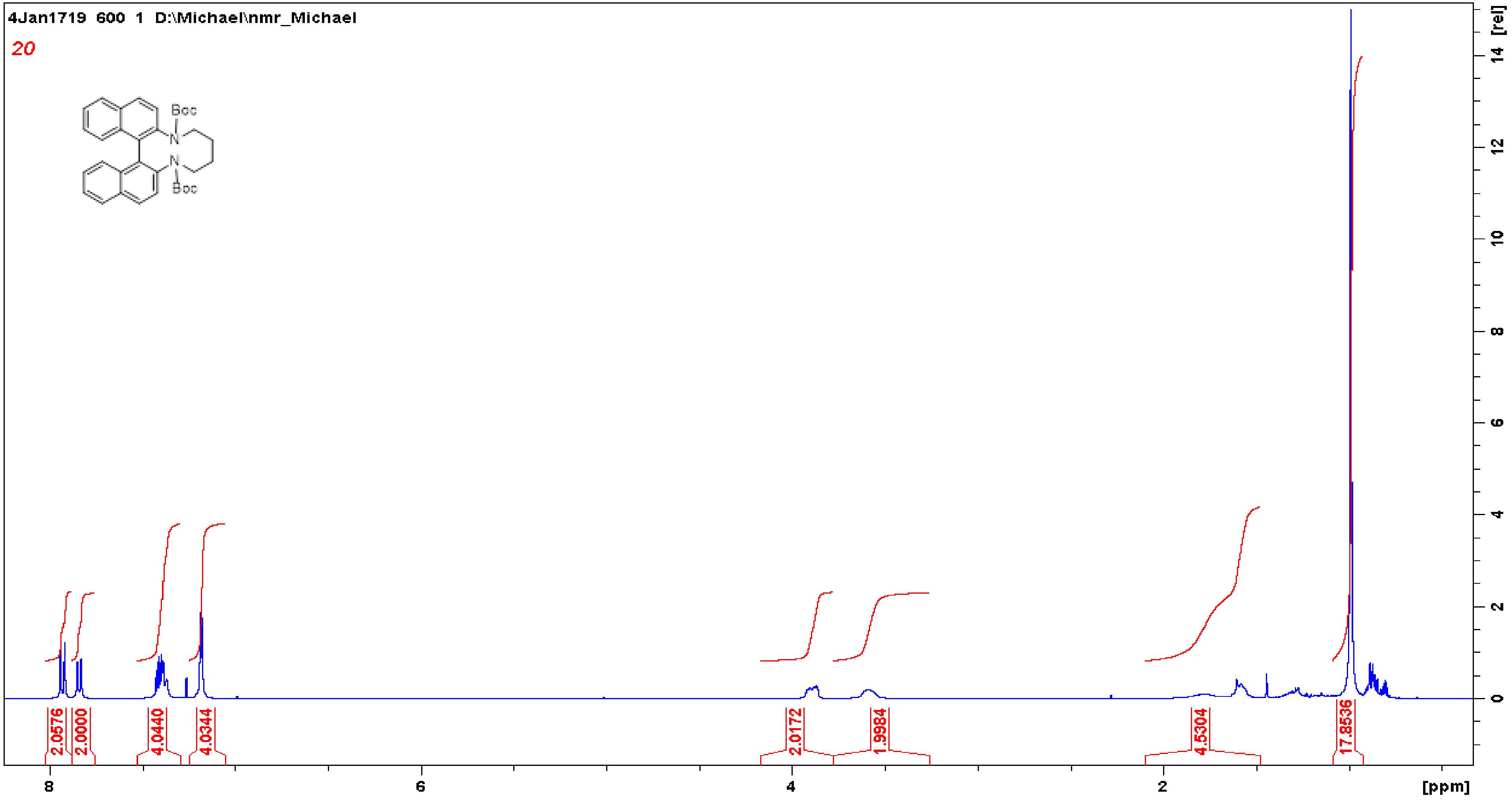
4Jan1719 600 1 D:\Michael\nmr_Michael
20
Boc
N
N
Boc
2.0576
2.0000
4.0440
4.0344
2.0172
1.9984
4.5304
17.8536
8
6
4
2
[ppm]
[rel]
14
12
10
8
6
4
2
0

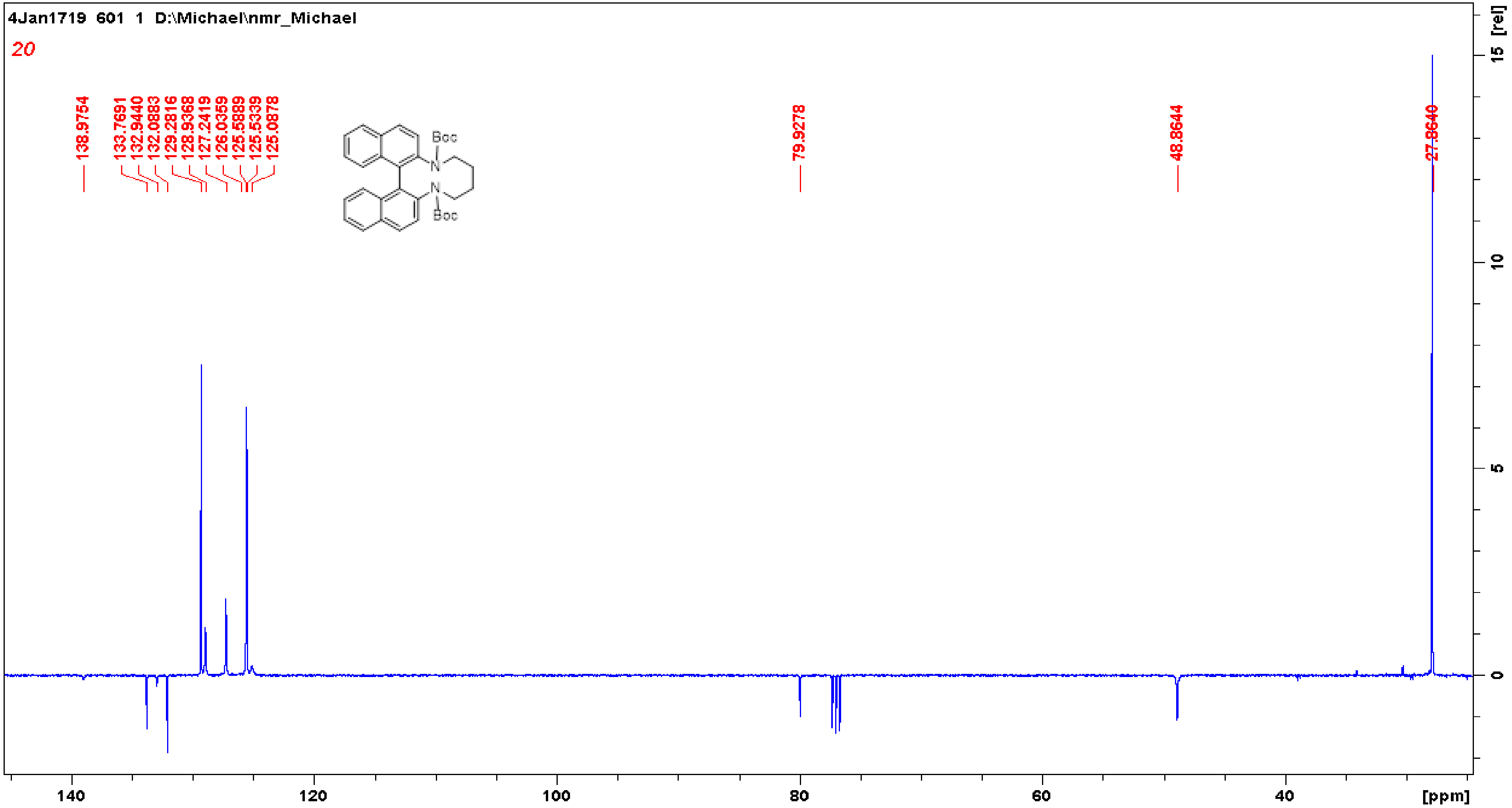
4Jan1719 601 1 D:\Michael\nmr_Michael
20
138.9754
133.7691
132.9440
132.0883
129.2816
128.9368
127.2419
126.0359
125.5889
125.5339
125.0878
79.9278
48.8644
27.8640
Boc
N
N
Boc
[rel]
15
10
5
0
140
120
100
80
60
40
[ppm]

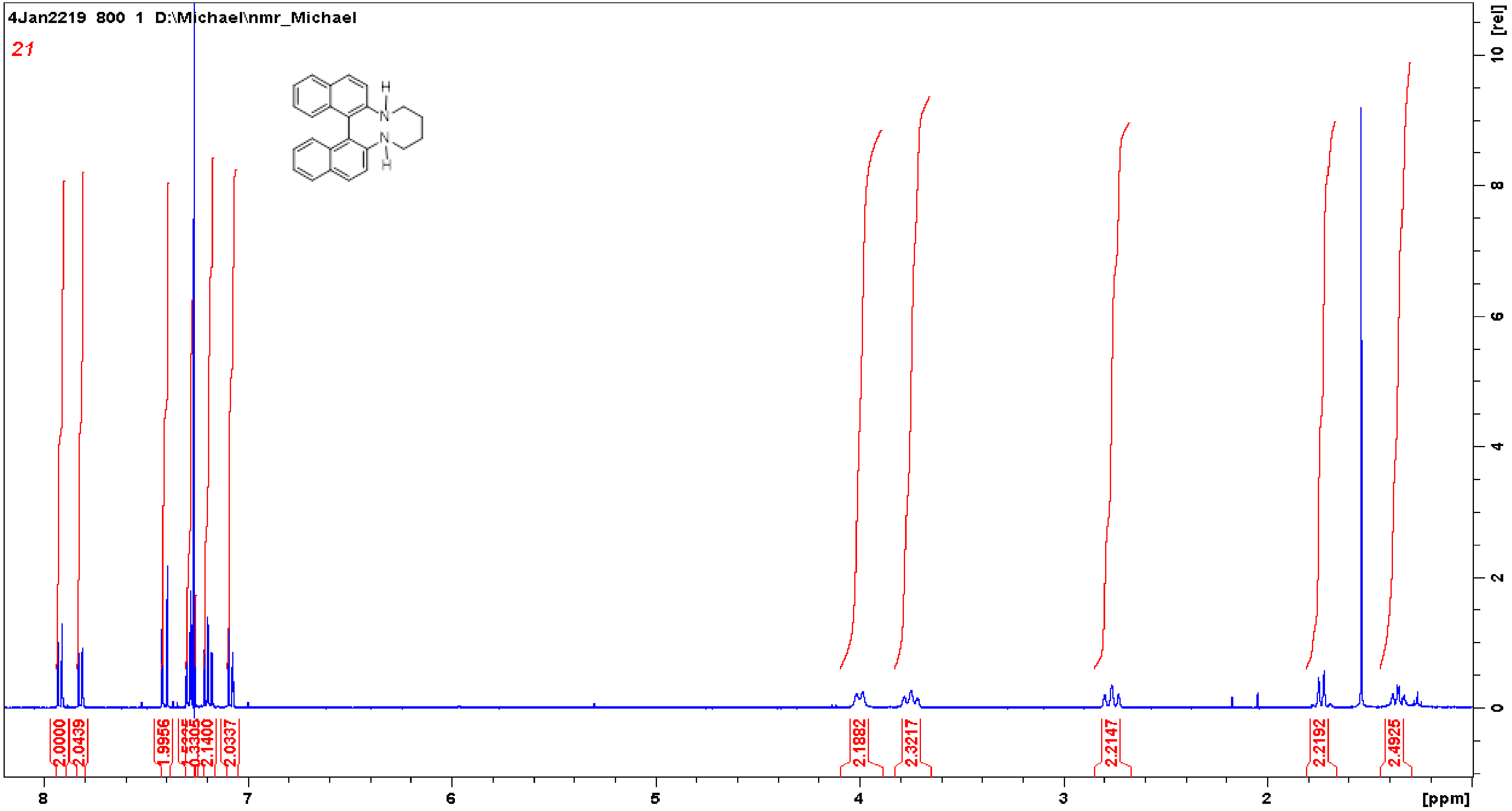
4Jan2219 800 1 D:\Michael\nmr_Michael
21
2.0000
2.0439
1.9956
1.5335
0.3305
2.1400
2.0337
2.1882
2.3217
2.2147
2.2192
2.4925
8
7
6
5
4
3
2
[ppm]
10
8
6
4
2
0
[rel]

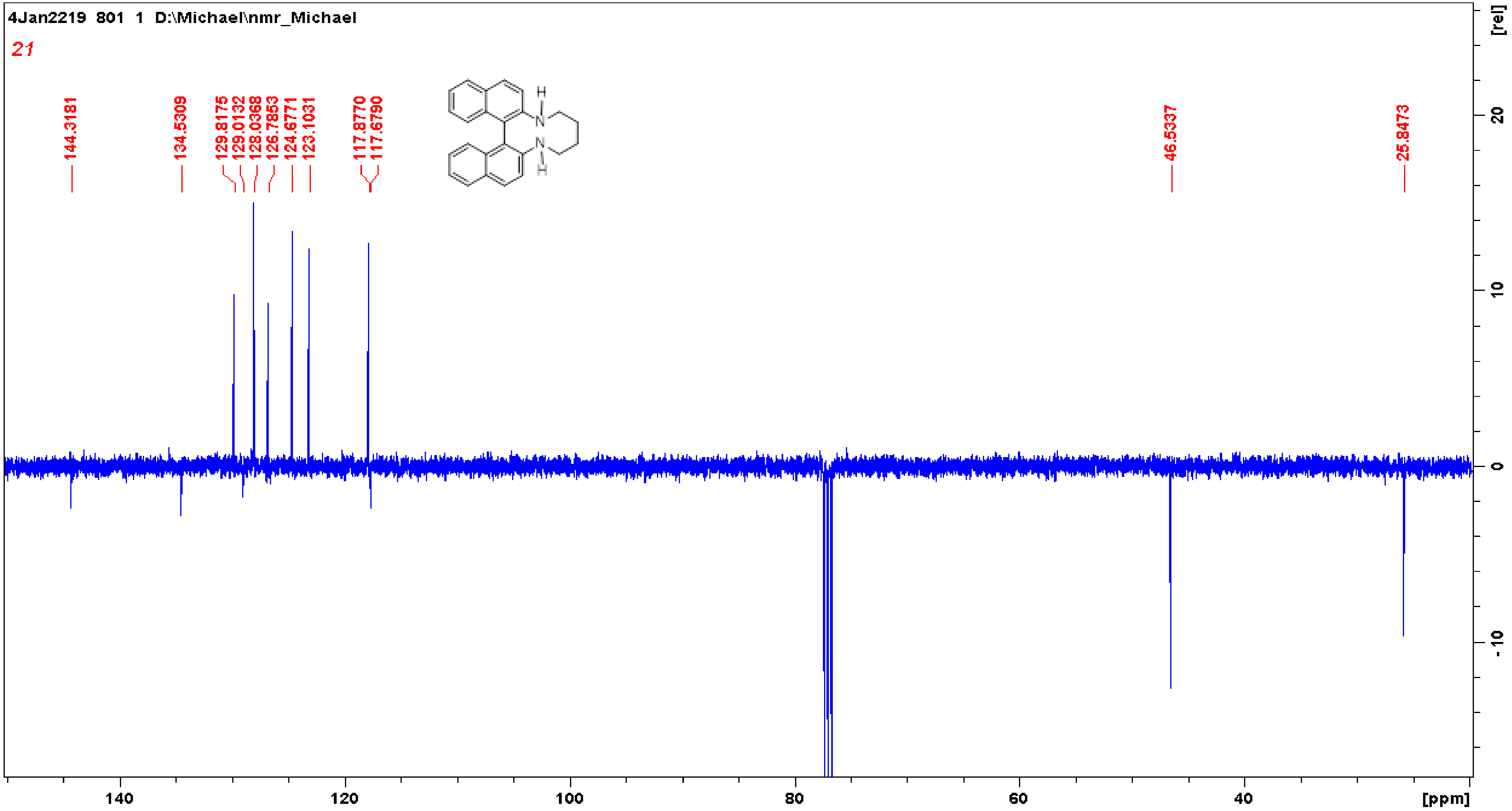

4Jan2219 801 1 D:\Michael\nmr_Michael
21
144.3181
134.5309
129.8175
129.0132
128.0368
126.7853
124.6771
123.1031
117.8770
117.6790
46.5337
25.8473
[rel]
20
10
0
-10
140
120
100
80
60
40
[ppm]

X-ray Analysis

The X-ray intensity data were measured on Bruker D8 Venture and X8 APEX2 diffractometer equipped with multilayer monochromators, Cu/Mo K/α INCOATEC micro focus sealed tubes and Oxford and Cryoflex2 cooling systems. The structure was solved by *direct methods or charge flipping* and refined by *full-matrix least-squares techniques*. Non-hydrogen atoms were refined with *anisotropic displacement parameters*. Hydrogen atoms were inserted at calculated positions and refined with riding model. The following software was used: *Bruker SAINT software package*[1] using a narrow-frame algorithm for frame integration, *SADABS*[2] for absorption correction, *OLEX2*[3] for structure solution, refinement, molecular diagrams and graphical user-interface, *Shelxle*[4] for refinement and graphical user-interface *SHELXS-2015*[5] for structure solution, *SHELXL-2015*[6] for refinement, *Platon*[7] for symmetry check. Experimental data and CCDC-Codes Experimental data and CCDC-Code (Available online: http://www.ccdc.cam.ac.uk/conts/retrieving.html) can be found in Table S1. Crystal data, data collection parameters, and structure refinement details are given in Tables S2 to S27. Crystal structures and packing are visualized in Figures S1 to S17.

Table S1 Experimental parameter and CCDC-Codes.

Sample	Machine	Source	Temp.	Detector Distance	Time/ Frame	#Frames	Frame width	CCDC
	Bruker		[K]	[mm]	[s]		[°]	
cis-**1**	X8	Mo	120	35	40	497	0.500	1923065
trans-**1**	X8	Mo	120	35	3	1110	0.500	1923066
**4a**	X8	Mo	100	40	60	900	1.000	1923067
**4c**	X8	Mo	100	40	10	3444	1.000	1923068
cis-**5a**	X8	Mo	120	35	10	3914	1.000	1923069
trans-**5a**	X8	Mo	120	35	20	1935	1.000	1923070
cis-**5d**	X8	Mo	120	35	60	857	0.500	1923071
trans-**5d**	X8	Mo	100	35	5	802	0.500	1923072
7	X8	Mo	130	35	5	1645	0.500	1923073
**9**	X8	Mo	130	40	30	592	0.500	1923074
**11**	D8	Mo	100	30	5	2022	0.500	1923075
**12**	D8	Cu	100	30	4	4912	0.700	1923076
**21**	D8	Mo	100	40	1	496	0.500	1923077

(*Z*)-11,12,15,16-Tetrahydrodinaphtho[2,1-b:1',2'-d][1,6]diazecine, *cis*-**1**

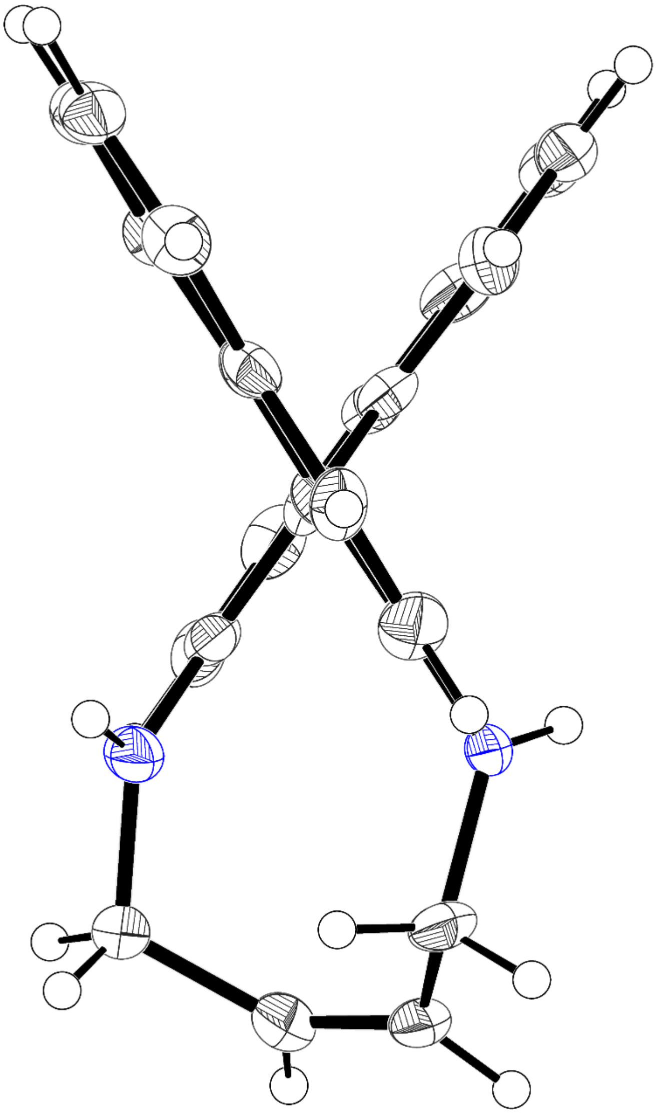

Figure S1 Crystal structure of *cis*-**1** (*S*-configuration). Second independent molecule in the asymmetric unit omitted for clarity. C-C Bond precision: 0.0066 Å.

Table S2 Sample and crystal data of *cis*-**1**.

**Chemical formula**	$C_{24}H_{20}N_2$	**Crystal system**	monoclinic	
**Formula weight [g/mol]**	336.42	**Space group**	*P2₁/n*	
**Temperature [K]**	120	**Z**	8	
**Measurement method**	\f and \w scans	**Volume [Å^3]**	3455.2(17)	
**Radiation (Wavelength [Å])**	MoKα (λ = 0.71073)	**Unit cell dimensions [Å] and [°]**	14.133(5)	90
**Crystal size / [mm^3]**	0.17 × 0.07 × 0.01		11.145(3)	105.704(13)
**Crystal habit**	clear colourless		22.787(6)	90
**Density (calculated) / [g/cm^3]**	1.293	**Absorption coefficient / [mm^{-1}]**	0.076	
**Abs. correction Tmin**	0.5686	**Abs. correction Tmax**	0.7452	
**Abs. correction type**	multiscan	**F(000) [e$^-$]**	1424	

Table S3 Data collection and structure refinement of *cis*-**1**.

**Index ranges**	-17 ≤ h ≤ 14, -13 ≤ k ≤ 11, -26 ≤ l ≤ 27	**Theta range for data collection [°]**	4.1 to 50.696	
**Reflections number**	17942	**Data / restraints / parameters**	6321/0/469	
**Refinement method**	Least squares	**Final R indices**	all data	R1 = 0.2364, wR2 = 0.2084
**Function minimized**	Σ w(F_o^2 - F_c^2)^2		I>2σ(I)	R1 = 0.0772, wR2 = 0.1482
**Goodness-of-fit on F^2**	0.936	**Weighting scheme**	w=1/[σ^2(F_o^2)+(0.0654P)^2]	
**Largest diff. peak and hole [e Å^{-3}]**	0.41/-0.43		where P=(F_o^2+2F_c^2)/3	

(*E*)-11,12,15,16-Tetrahydrodinaphtho[2,1-b:1',2'-d][1,6]diazecine, *trans*-**1**

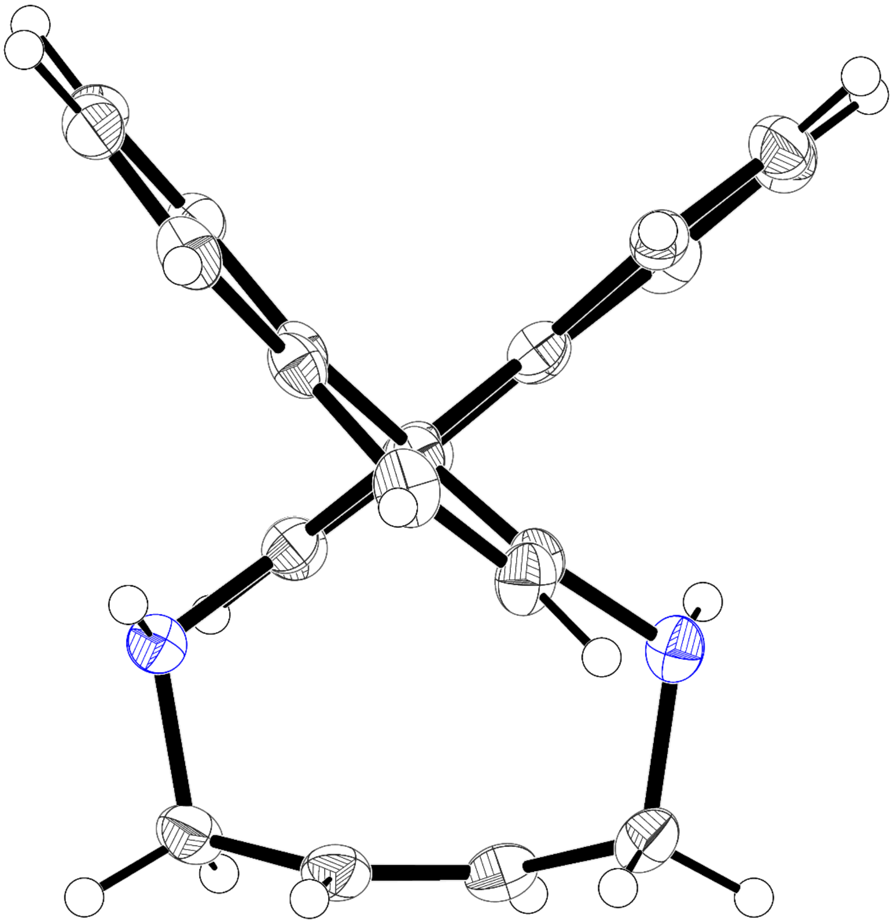

Figure S2 Asymmetric unit of *trans*-**1** (*S*-configuration). C-C Bond precision: 0.0030 Å. As the spacegroup *P2₁2₁2₁* is a chiral one only one of the two forms crystalized. The proof for one of these two could not be done by x-ray methods. The corresponding Flack parameter was not in the range of trust.

Table S4 Sample and crystal data of *trans*-**1**.

**Chemical formula**	$C_{24}H_{20}N_2$	**Crystal system**	orthorhombic	
**Formula weight [g/mol]**	336.42	**Space group**	$P2_12_12_1$	
**Temperature [K]**	120	**Z**	4	
**Measurement method**	\f and \w scans	**Volume [Å^3]**	1755.68(12)	
**Radiation (Wavelength [Å])**	MoKα (λ = 0.71073)	**Unit cell dimensions [Å] and [°]**	10.5405(4)	90
**Crystal size / [mm^3]**	0.26 × 0.2 × 0.2		11.5662(5)	90
**Crystal habit**	clear colourless		14.4010(5)	90
**Density (calculated) / [g/cm^3]**	1.273	**Absorption coefficient / [mm^{-1}]**	0.075	
**Abs. correction Tmin**	0.6929	**Abs. correction Tmax**	0.746	
**Abs. correction type**	multiscan	**F(000) [e$^-$]**	712	

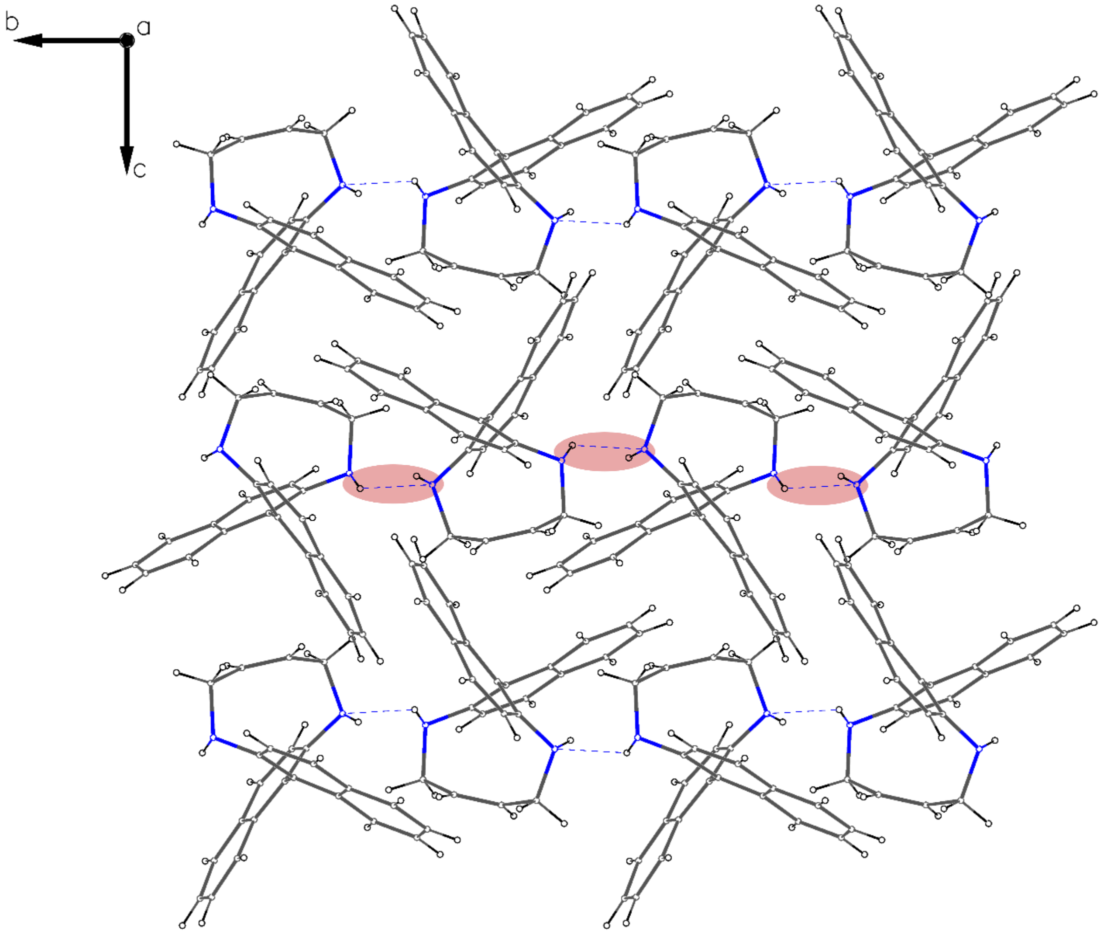

Figure S3 Orthogonal projection packing of *trans*-**1** along axis a. The analysis of hydrogen bonded molecular aggregates by Platon detected the Infinite 1D-chain with base vector: [0 1 0].

Table S5 Data collection and structure refinement of *trans*-**1**.

**Index ranges**	$-12 \le h \le 12$, $-13 \le k \le 13$, $-15 \le l \le 17$	**Theta range for data collection [°]**	4.516 to 50.694	
**Reflections number**	20450	**Data / restraints / parameters**	3214/0/235	
**Refinement method**	Least squares	**Final R indices**	all data	R1 = 0.0361, wR2 = 0.0848
**Function minimized**	Σ w(F_o^2 - F_c^2)^2		I>2σ(I)	R1 = 0.0333, wR2 = 0.0827
**Goodness-of-fit on F^2**	1.062	**Weighting scheme**	w=1/[$\sigma^2(F_o^2)$+(0.0449P)^2 +0.3440P]	
**Largest diff. peak and hole [e Å^{-3}]**	0.16/-0.18		where P=(F_o^2+2F_c^2)/3	

N,N'-([1,1'-Binaphthalene]-2,2'-diyl)bis(*N*-allylmethanesulfonamide) **4a**

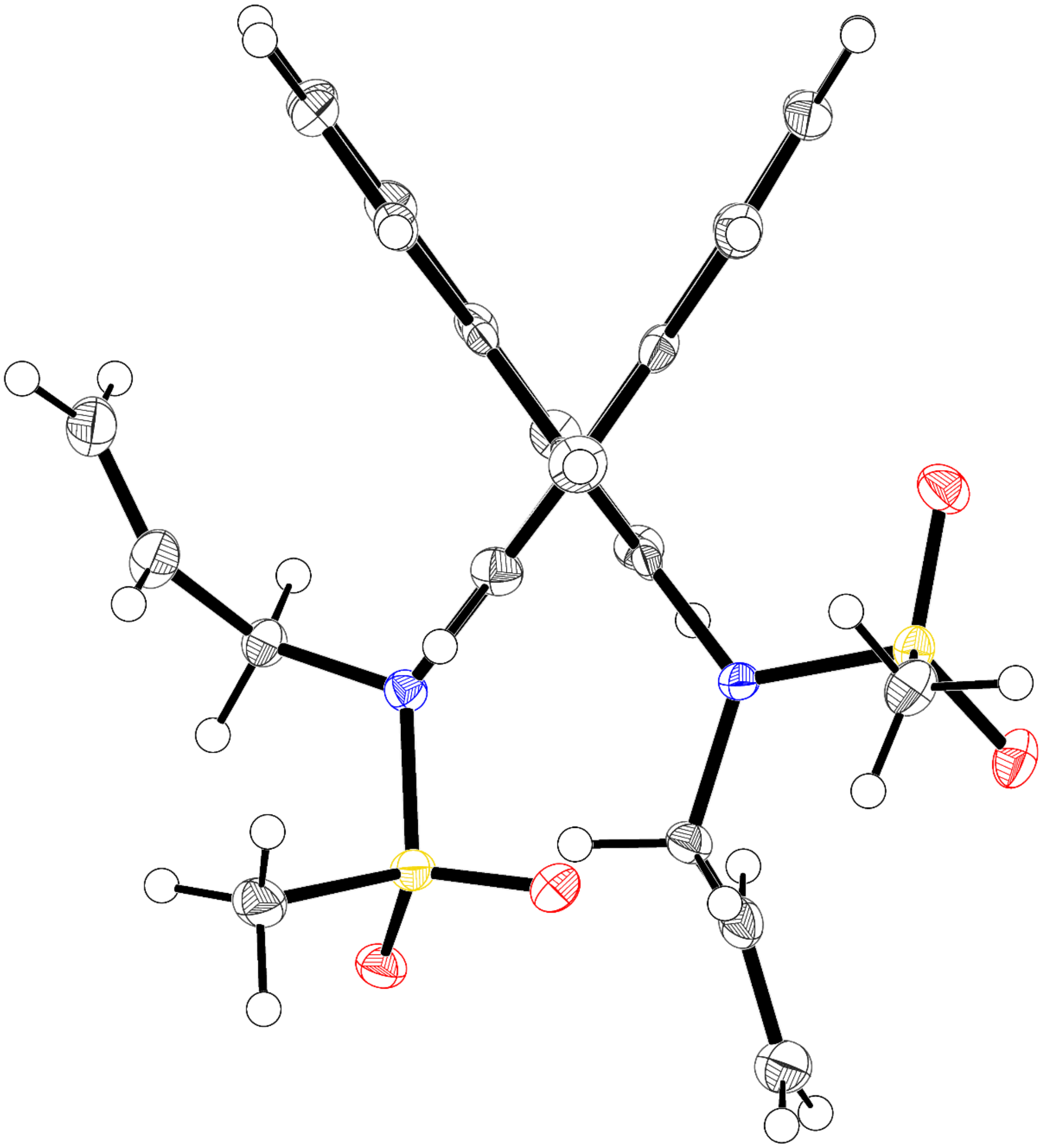

Figure S4 Asymmetric unit of **4a** (*R*-configuration). C-C Bond precision: 0.0046 Å. As the spacegroup $P2_12_12_1$ is a chiral one only one of the two forms crystalized. The anomalous dispersion with the Flack parameter x = -0.07(5) is a good proof for the crystallization in *R*-configuration.

Table S6 Sample and crystal data.of **4a.**

**Chemical formula**	$C_{28}H_{28}N_2O_4S_2$	**Crystal system**	orthorhombic	
**Formula weight [g/mol]**	520.64	**Space group**	*P212121*	
**Temperature [K]**	100.15	**Z**	4	
**Measurement method**	\f and \w scans	**Volume [Å^3]**	2487.8(5)	
**Radiation (Wavelength [Å])**	MoKα (λ = 0.71073)	**Unit cell dimensions [Å] and [°]**	9.7339(11)	90
**Crystal size / [mm^3]**	0.1 × 0.1 × 0.03		11.2343(11)	90
**Crystal habit**	clear colourless block		22.750(3)	90
**Density (calculated) / [g/cm^3]**	1390	**Absorption coefficient / [mm^{-1}]**	0.253	
**Abs. correction Tmin**	0.5836	**Abs. correction Tmax**	0.7452	
**Abs. correction type**	multiscan	**F(000) [e$^-$]**	1096.0	

Table S7 Data collection and structure refinement of **4a**

**Index ranges**	$-11 \leq h \leq 11$, $-10 \leq k \leq 13$, $-27 \leq l \leq 27$	**Theta range for data collection [°]**	5.096 to 51.432	
**Reflections number**	32379	**Data / restraints / parameters**	4660/0/328	
**Refinement method**	Least squares	**Final R indices**	all data	R1 = 0.0429, wR2 = 0.0829
**Function minimized**	Σ w(F_o^2 - F_c^2)^2		I>2σ(I)	R1 = 0.0348, wR2 = 0.0791
**Goodness-of-fit on F^2**	1034	**Weighting scheme**	w=1/[σ^2(F_o^2)+(0.0367P)^2 +0.3250P]	
**Largest diff. peak and hole [e Å^{-3}]**	0.23/-0.28		where P=(F_o^2+2F_c^2)/3	

N,N'-([1,1'-Binaphthalene]-2,2'-diyl)bis(N-allyl-2,2,2-triflouroacetamide **4c**

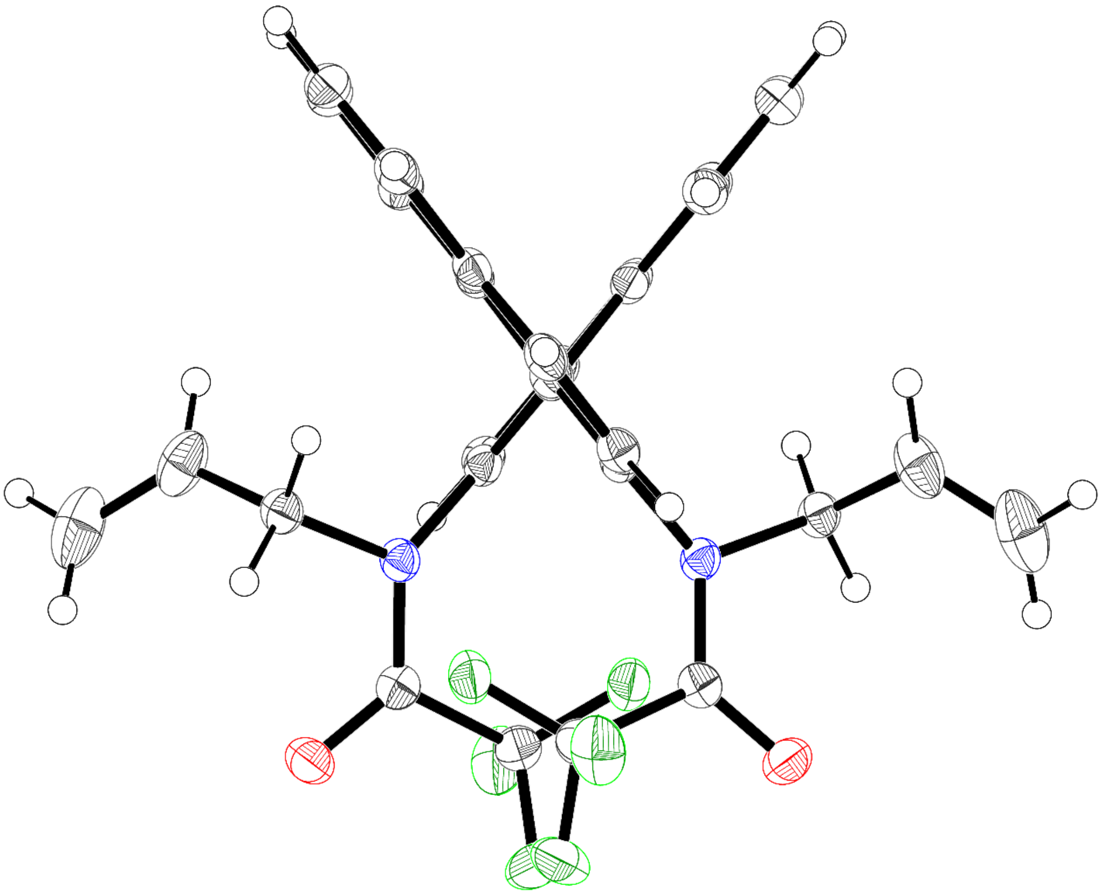

Figure S5 Crystal structure of **4c** (*S*-configuration). C-C Bond precision: 0.0019 Å.

Table S8 Sample and crystal data of **4c**.

**Chemical formula**	$C_{30}H_{22}F_6N_2O_2$	**Crystal system**	monoclinic	
**Formula weight [g/mol]**	556.49	**Space group**	*C2/c*	
**Temperature [K]**	100	**Z**	4	
**Measurement method**	\f and \w scans	**Volume [Å^3]**	2518.17(13)	
**Radiation (Wavelength [Å])**	MoKα (λ = 0.71073)	**Unit cell dimensions [Å] and [°]**	13.8966(4)	90
**Crystal size / [mm^3]**	0.5 × 0.2 × 0.2		13.3655(4)	103.583(2)
**Crystal habit**	clear brown		13.9480(4)	90
**Density (calculated) / [g/cm^3]**	1.468	**Absorption coefficient / [mm^{-1}]**	0.123	
**Abs. correction Tmin**	0.6911	**Abs. correction Tmax**	0.746	
**Abs. correction type**	multiscan	**F(000) [e$^-$]**	1144	

Table S9 Data collection and structure refinement of **4c**.

**Index ranges**	$-19 \leq h \leq 19$, $-18 \leq k \leq 18$, $-19 \leq l \leq 19$	**Theta range for data collection [°]**	4.812 to 59.992	
**Reflections number**	75275	**Data / restraints / parameters**	3655/0/181	
**Refinement method**	Least squares	**Final R indices**	all data	R1 = 0.0627, wR2 = 0.1186
**Function minimized**	$\Sigma\ w(F_o^2 - F_c^2)^2$		$I > 2\sigma(I)$	R1 = 0.0439, wR2 = 0.1103
**Goodness-of-fit on F^2**	1.091	**Weighting scheme**	$w=1/[\sigma^2(F_o^2)+(0.0539P)^2+1.5315P]$	
**Largest diff. peak and hole [e Å^{-3}]**	0.48/-0.24		where $P=(F_o^2+2F_c^2)/3$	

(*Z*)-11,16-Bis(methylsulfonyl)-11,12,15,16-tetrahydrodinaphtho[2,1-b:1',2'-d][1,6]diazecine *cis*-**5a**

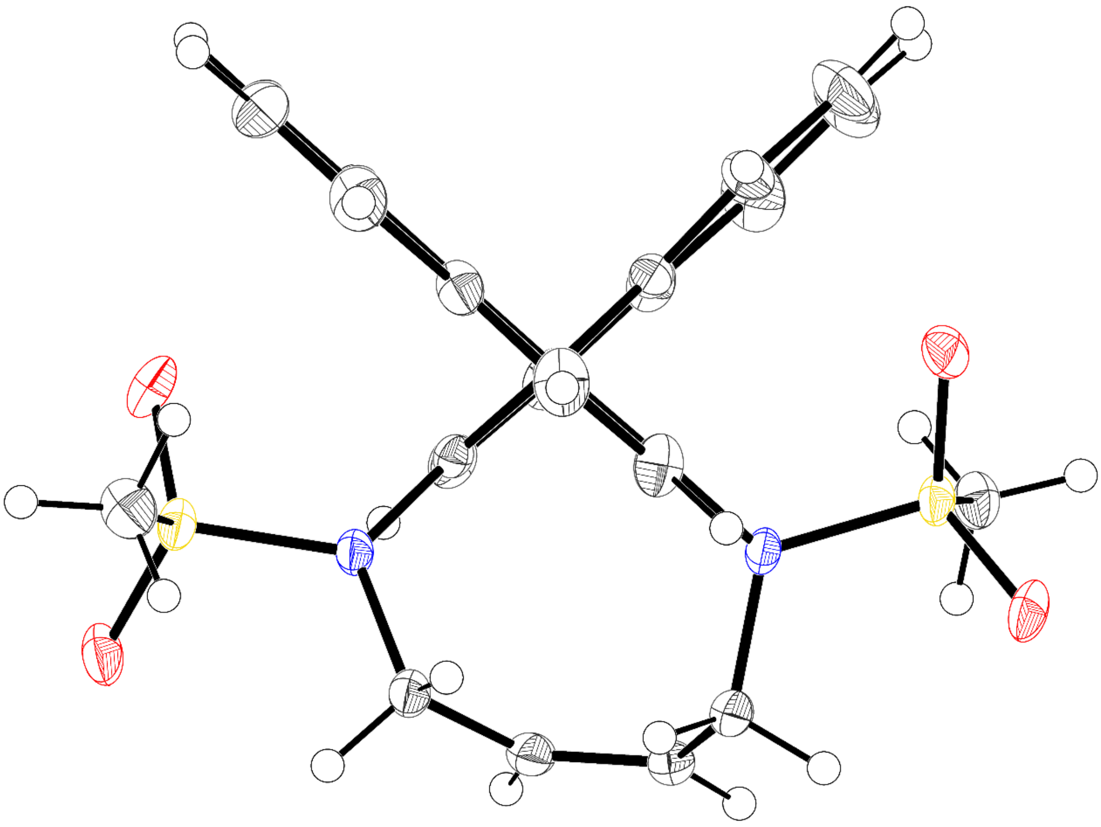

Figure S6 Crystal structure of *cis* **5a** (*S*-configurationm). C-C Bond precision: 0.0032 Å. Solvent omitted for clarity. Platon detected one short intramolecular contact. The resulting alert is wrong because the according atoms are from different parts in disordered co-crystalized solvent. These alerts could not be avoided. Also not with the help of Mr. Spek (developer of Platon).

Table S10 Sample and crystal data of *cis*-**5a**

**Chemical formula**	$C_{27}H_{28}Cl_3N_2O_4S_2$	**Crystal system**	triclinic	
**Formula weight [g/mol]**	614.98	**Space group**	*P-1*	
**Temperature [K]**	120	**Z**	2	
**Measurement method**	\f and \w scans	**Volume [Å^3]**	1416.00(12)	
**Radiation (Wavelength [Å])**	MoKα (λ = 0.71073)	**Unit cell dimensions [Å] and [°]**	10.7270(5)	86.543(2)
**Crystal size / [mm^3]**	0.4 × 0.3 × 0.25		11.3356(5)	81.059(3)
**Crystal habit**	clear colourless block		12.4265(6)	71.569(2)
**Density (calculated) / [g/cm^3]**	1442	**Absorption coefficient / [mm^{-1}]**	0.508	
**Abs. correction Tmin**	0.7038	**Abs. correction Tmax**	0.7460	
**Abs. correction type**	multiscan	**F(000) [e$^-$]**	638.0	

Table S11 Data collection and structure refinement of *cis*-**5a**.

**Index ranges**	-15 ≤ h ≤ 15, -15 ≤ k ≤ 15, -17 ≤ l ≤ 17	**Theta range for data collection [°]**	4.044 to 60.132	
**Reflections number**	111464	**Data / restraints / parameters**	8253/0/355	
**Refinement method**	Least squares	**Final R indices**	all data	R1 = 0.0607, wR2 = 0.1540
**Function minimized**	Σ w(F_o^2 - F_c^2)^2		I>2σ(I)	R1 = 0.0482, wR2 = 0.1388
**Goodness-of-fit on F^2**	1089	**Weighting scheme**	w=1/[σ^2(F_o^2)+(0.0764P)^2 +1.8185P]	
**Largest diff. peak and hole [e Å^{-3}]**	1.22/-1.10		where P=(F_o^2+2F_c^2)/3	

(E)-11,16-Bis(methylsulfonyl)-11,12,15,16-tetrahydrodinaphtho[2,1-b:1',2'-d][1,6]diazecine *trans*-**5a**

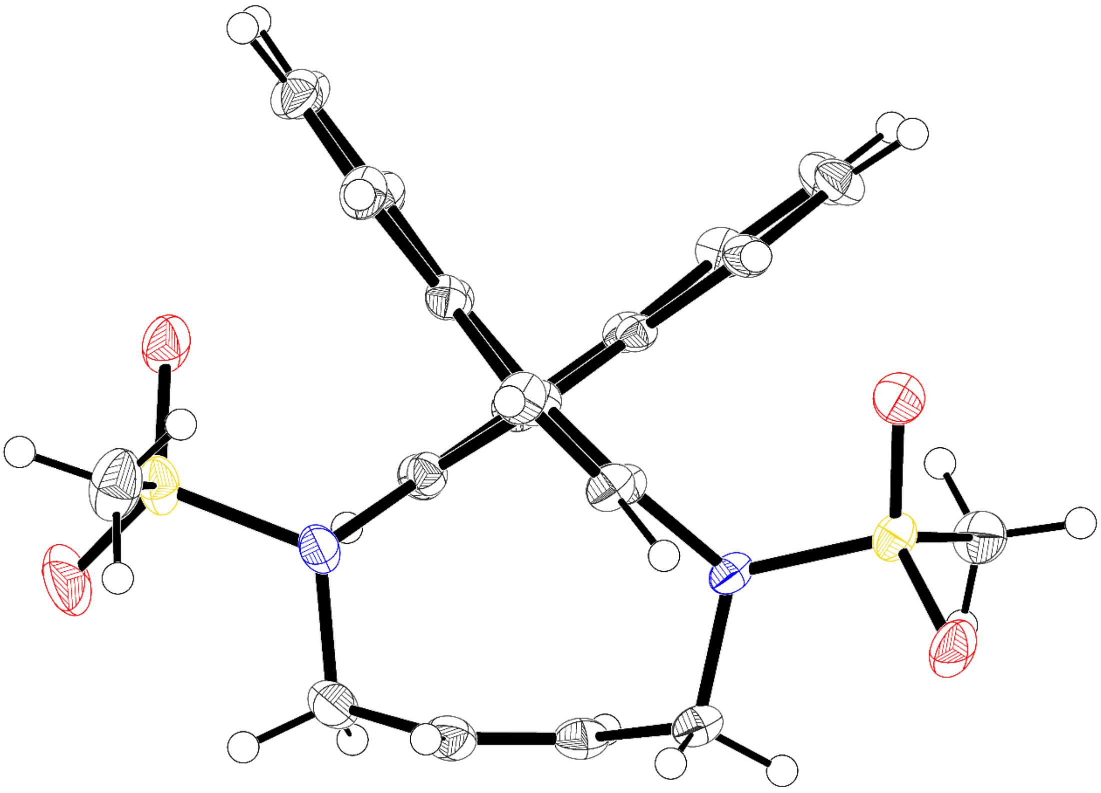

Figure S7 Crystal structure of *trans*-**5a** (*S*-configuration). C-C Bond precision: 0.0022 Å.

Table S12 Sample and crystal data of *trans*-**5a**.

**Chemical formula**	$C_{52}H_{48}N_4O_8S_4$	**Crystal system**	monoclinic	
**Formula weight [g/mol]**	985.18	**Space group**	*P21/n*	
**Temperature [K]**	120.15	**Z**	2	
**Measurement method**	\f and \w scans	**Volume [Å^3]**	2326.62(14)	
**Radiation (Wavelength [Å])**	MoKα (λ = 0.71073)	**Unit cell dimensions [Å] and [°]**	10.8879(4)	90
**Crystal size / [mm^3]**	0.2 × 0.11 × 0.07		13.6381(5)	90.396(2)
**Crystal habit**	clear colourless		15.6689(5)	90
**Density (calculated) / [g/cm^3]**	1.406	**Absorption coefficient / [mm^{-1}]**	0.266	
**Abs. correction Tmin**	0.6776	**Abs. correction Tmax**	0.746	
**Abs. correction type**	multiscan	**F(000) [e$^-$]**	1032	

Table S13 Data collection and structure refinement of *trans*-**5a**.

**Index ranges**	$-15 \le h \le 15$, $-19 \le k \le 19$, $-21 \le l \le 22$	**Theta range for data collection [°]**	3.96 to 60.144	
**Reflections number**	92527	**Data / restraints / parameters**	6775/0/403	
**Refinement method**	Least squares	**Final R indices**	all data	R1 = 0.0693, wR2 = 0.1039
**Function minimized**	$\Sigma\ w(F_o^2 - F_c^2)^2$		I>2σ(I)	R1 = 0.0406, wR2 = 0.0907
**Goodness-of-fit on F^2**	1.017	**Weighting scheme**	$w=1/[\sigma^2(F_o^2)+(0.0391P)^2+1.5784P]$	
**Largest diff. peak and hole [e Å^{-3}]**	0.33/-0.58		where $P=(F_o^2+2F_c^2)/3$	

Di-*tert*-butyl (*Z*)-12,15-dihydrodinaphtho[2,1-b:1',2'-d][1,6]diazecine-11,16-dicarboxylate *cis*-**5d**

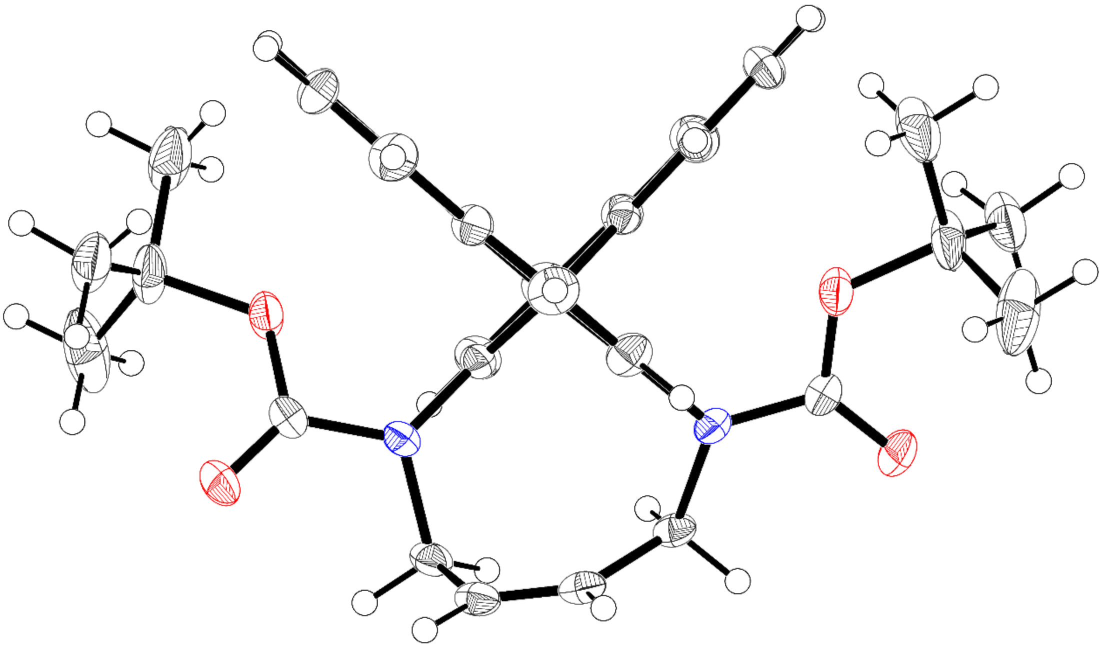

Figure S8 Crystal structure of *cis*-**5d** (*S*-configuration). C-C Bond precision: 0.0029 Å. Disorder omitted for clarity. Main residue disorder 10%. Platon detected short intramolecular contacts. These three resulting alerts are wrong because the according atoms are from different parts. These alerts could not be avoided. Also not with the help of Mr. Spek (developer of Platon).

Table S14 Sample and crystal data of *cis*-**5d**.

**Chemical formula**	$C_{34}H_{38}N_2O4$	**Crystal system**	monoclinic	
**Formula weight [g/mol]**	538.66	**Space group**	*C2/c*	
**Temperature [K]**	100	**Z**	4	
**Measurement method**	\f and \w scans	**Volume [Å^3]**	2837.5(12)	
**Radiation (Wavelength [Å])**	MoKα (λ = 0.71073)	**Unit cell dimensions [Å] and [°]**	19.795(4)	90
**Crystal size / [mm^3]**	0.15 × 0.12 × 0.08		12.914(4)	124.816(10)
**Crystal habit**	clear colourless block		13.521(4)	90
**Density (calculated) / [g/cm^3]**	1261	**Absorption coefficient / [mm^{-1}]**	0.082	
**Abs. correction Tmin**	0.6689	**Abs. correction Tmax**	0.7460	
**Abs. correction type**	multiscan	**F(000) [e$^-$]**	1152.0	

Table S15 Data collection and structure refinement of *cis* **5d**.

**Index ranges**	-26 ≤ h ≤ 27, -18 ≤ k ≤ 18, -19 ≤ l ≤ 19	**Theta range for data collection [°]**	4.382 to 60.364	
**Reflections number**	14987	**Data / restraints / parameters**	4180/3/210	
**Refinement method**	Least squares	**Final R indices**	all data	R1 = 0.1009, wR2 = 0.1535
**Function minimized**	Σ w(F_o^2 - F_c^2)^2		I>2σ(I)	R1 = 0.0604, wR2 = 0.1344
**Goodness-of-fit on F^2**	1025	**Weighting scheme**	w=1/[σ2(Fo2)+(0.0606P)2 +2.0466P]	
**Largest diff. peak and hole [e Å^{-3}]**	0.32/-0.26		where P=(F_o^2+2F_c^2)/3	

Di-*tert*-butyl (*E*)-12,15-dihydrodinaphtho[2,1-b:1',2'-d][1,6]diazecine-11,16-dicarboxylate *trans*-**5d**

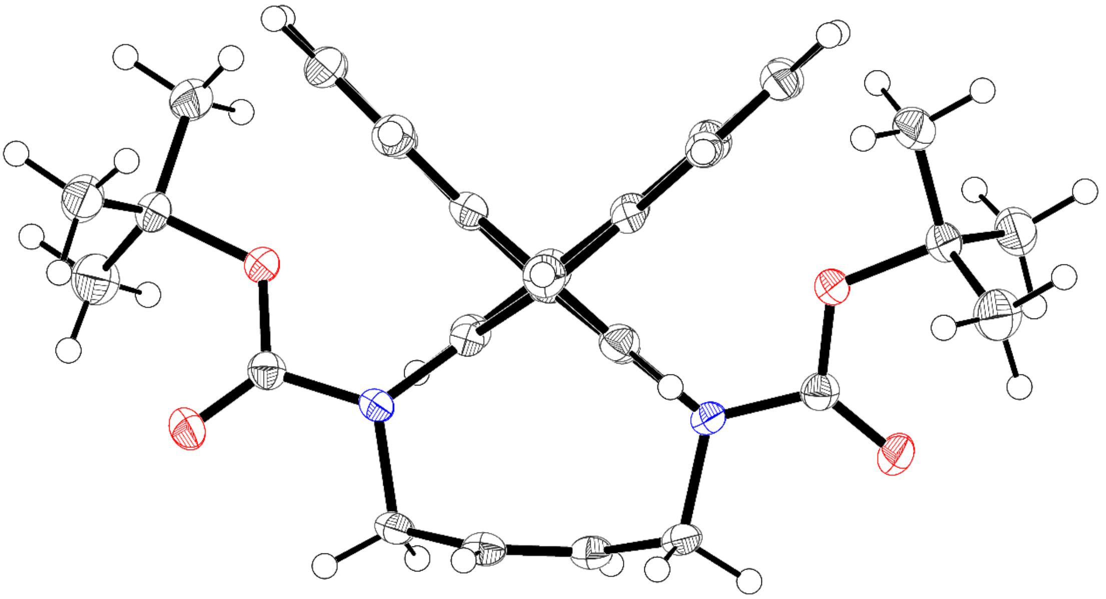

Figure S9 Crystal structure of *trans*-**5d** (*S*-configuration). C-C Bond precision: 0.0031 Å.

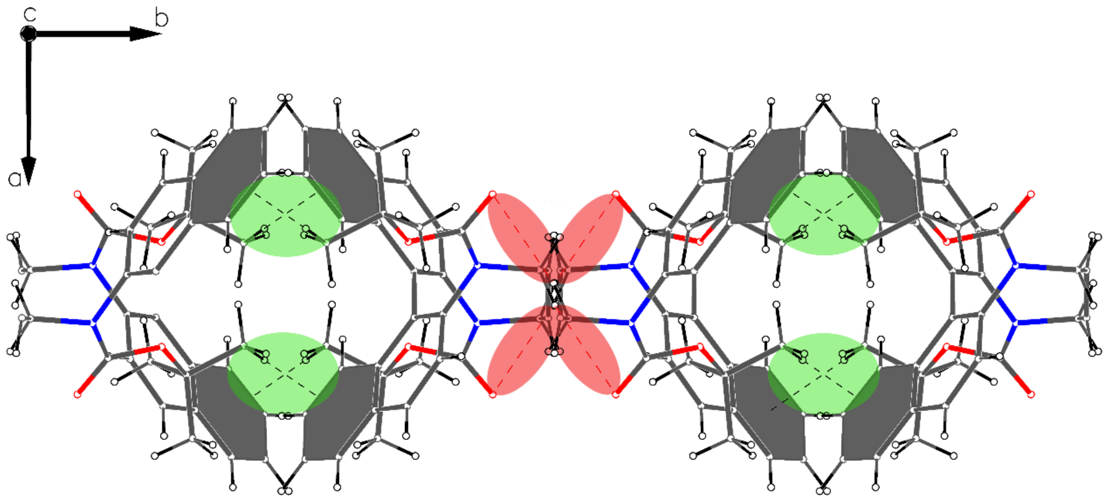

Figure S10 Packing of *trans*-**5d** along axis c. Visualization of two classes of intermolecular interactions: red highlighted weak C-H—O interactions and green highlighted C-H—PI Ring interactions.

Table S16 Sample and crystal data of *trans*-**5d**.

**Chemical formula**	$C_{34}H_{36}N_2O_4$	**Crystal system**	monoclinic	
**Formula weight [g/mol]**	536.65	**Space group**	*C2/c*	
**Temperature [K]**	100.0	**Z**	4	
**Measurement method**	\f and \w scans	**Volume [Å^3]**	2828.9(3)	
**Radiation (Wavelength [Å])**	MoKα (λ = 0.71073)	**Unit cell dimensions [Å] and [°]**	19.7641(15)	90
**Crystal size / [mm^3]**	0.2 × 0.12 × 0.1		12.6467(8)	127.156(3)
**Crystal habit**	clear colourless block		14.2008(9)	90
**Density (calculated) / [g/cm^3]**	1260	**Absorption coefficient / [mm^{-1}]**	0.082	
**Abs. correction Tmin**	0.6549	**Abs. correction Tmax**	0.7460	
**Abs. correction type**	multiscan	**F(000) [e$^-$]**	1144.0	

Table S17 Data collection and structure refinement of *trans*-**5d**.

**Index ranges**	$-27 \leq h \leq 26$, $-17 \leq k \leq 17$, $-20 \leq l \leq 17$	**Theta range for data collection [°]**	4.332 to 60.264	
**Reflections number**	13784	**Data / restraints / parameters**	4156/0/184	
**Refinement method**	Least squares	**Final R indices**	all data	R1 = 0.1131, wR2 = 0.1557
**Function minimized**	Σ w(F_o^2 - F_c^2)^2		I>2σ(I)	R1 = 0.0583, wR2 = 0.1295
**Goodness-of-fit on F^2**	1033	**Weighting scheme**	w=1/[σ2(Fo2)+(0.0617P)2]	
**Largest diff. peak and hole [e Å^{-3}]**	0.30/-0.30		where P=(F_o^2+2F_c^2)/3	

tert-Butyl 11-bromo-8-oxo-11,12-dihydro-8*H*-7,10-methanodinaphtho[2,1-d:1',2'-f][1]oxa[3,8]diazacycloundecine-13(10H)-carboxylate **7**

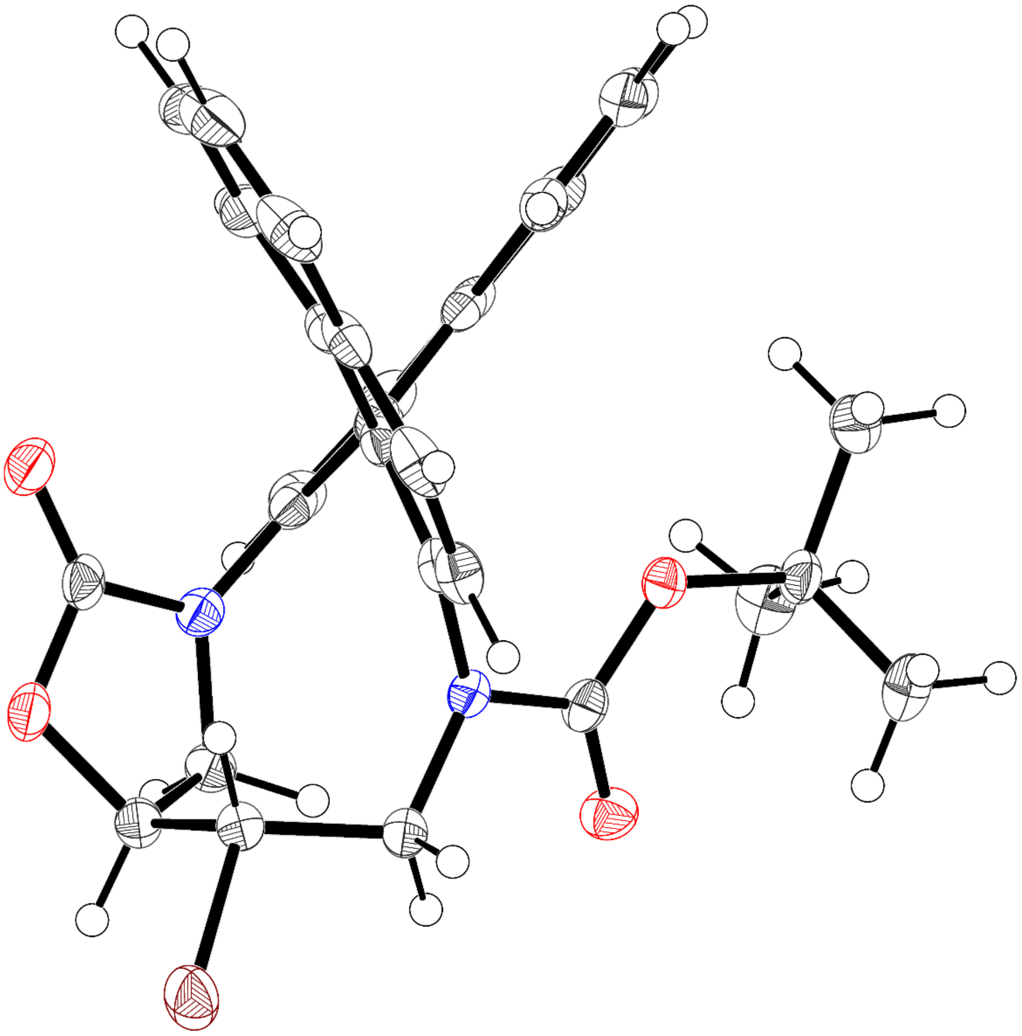

Figure S11 Crystal structure of **7** (*S*-configuration). C-C Bond precision: 0.0031 Å

Table S18 Sample and crystal data of **7**.

**Chemical formula**	$C_{30}H_{27}BrN_2O_4$	**Crystal system**	monoclinic	
**Formula weight [g/mol]**	559.44	**Space group**	*P21/n*	
**Temperature [K]**	130	**Z**	4	
**Measurement method**	\f and \w scans	**Volume [Å^3]**	2514.7(3)	
**Radiation (Wavelength [Å])**	MoKα (λ = 0.71073)	**Unit cell dimensions [Å] and [°]**	9.4794(6)	90
**Crystal size / [mm^3]**	0.23 × 0.14 × 0.14		21.6529(14)	92.249(2)
**Crystal habit**	clear colourless		12.2608(6)	90
**Density (calculated) / [g/cm^3]**	1.478	**Absorption coefficient / [mm^{-1}]**	1.675	
**Abs. correction Tmin**	0.6587	**Abs. correction Tmax**	0.746	
**Abs. correction type**	multiscan	**F(000) [e$^-$]**	1152	

Table S19 Data collection and structure refinement. of **7**

**Index ranges**	$-13 \leq h \leq 13$, $-30 \leq k \leq 30$, $-14 \leq l \leq 17$	**Theta range for data collection [°]**	4.694 to 60.292	
**Reflections number**	50380	**Data / restraints / parameters**	7358/0/337	
**Refinement method**	Least squares	**Final R indices**	all data	R1 = 0.0774, wR2 = 0.0997
**Function minimized**	Σ w(F_o^2 - F_c^2)^2		I>2σ(I)	R1 = 0.0411, wR2 = 0.0875
**Goodness-of-fit on F^2**	1.049	**Weighting scheme**	w=1/[$\sigma^2(F_o^2)+(0.0375P)^2+0.9399P$]	
**Largest diff. peak and hole [e Å^{-3}]**	0.78/-0.82		where $P=(F_o^2+2F_c^2)/3$	

Di-*tert*-butyl (8*R*,9*S*)-9-bromo-8-(bromomethyl)-9,10-dihydro-7*H*-dinaphtho[2,1-f:1',2'-h][1,5]diazonine-7,11(8H)-dicarboxylate **9**

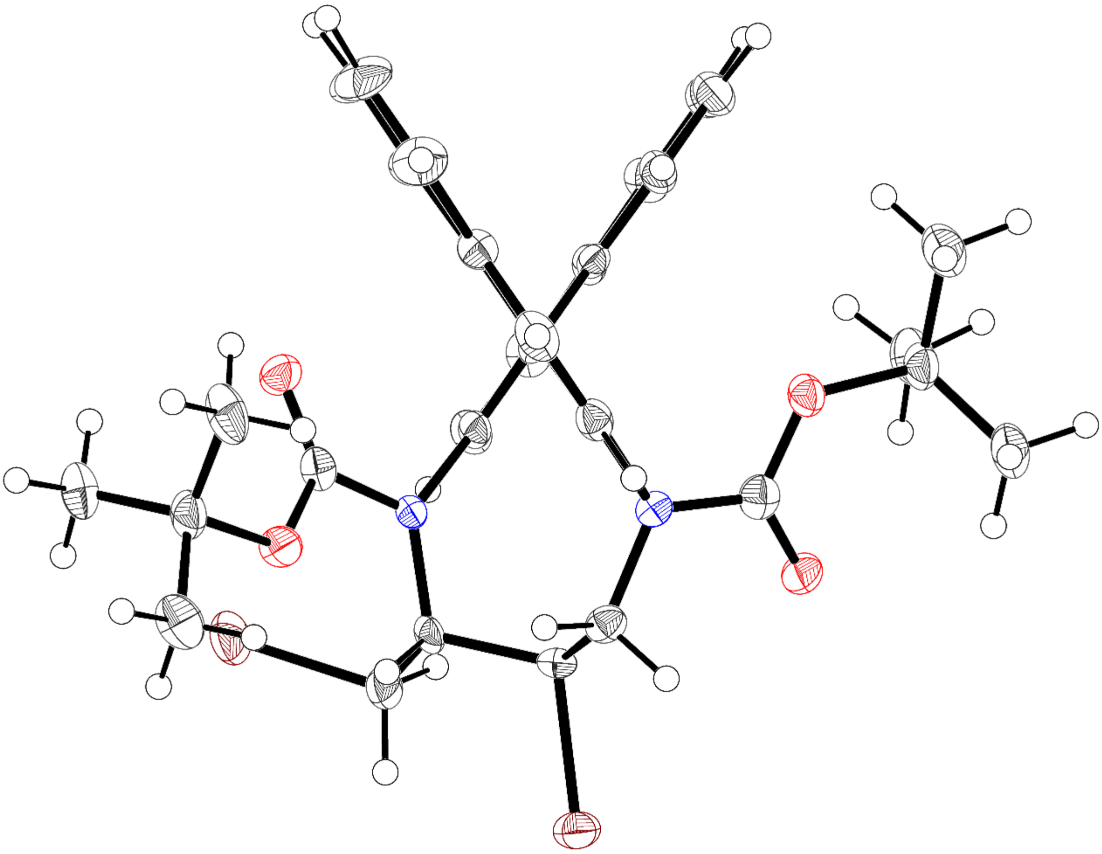

Figure S12 Crystal structure of **9** (*S*-configuration). C-C Bond precision: 0.0069 Å. Solvent omitted for clarity.

Table S20 Sample and crystal data of **9**.

**Chemical formula**	$C_{36}H_{40}Br_2N_2O_{4.5}$	**Crystal system**	monoclinic	
**Formula weight [g/mol]**	732.52	**Space group**	*C2/c*	
**Temperature [K]**	130	**Z**	8	
**Measurement method**	\f and \w scans	**Volume [Å^3]**	6648.4(15)	
**Radiation (Wavelength [Å])**	MoKα (λ = 0.71073)	**Unit cell dimensions [Å] and [°]**	23.929(2)	90
**Crystal size / [mm^3]**	0.11 × 0.11 × 0.1		12.375(2)	111.178(4)
**Crystal habit**	clear colourless		24.078(3)	90
**Density (calculated) / [g/cm^3]**	1.464	**Absorption coefficient / [mm^{-1}]**	2.482	
**Abs. correction Tmin**	0.606	**Abs. correction Tmax**	0.746	
**Abs. correction type**	multiscan	**F(000) [e$^-$]**	3008	

Table S21 Data collection and structure refinement of **9**.

**Index ranges**	$-22 \leq h \leq 28$, $-14 \leq k \leq 10$, $-28 \leq l \leq 29$	**Theta range for data collection [°]**	4.114 to 50.696	
**Reflections number**	16131	**Data / restraints / parameters**	6069/0/408	
**Refinement method**	Least squares	**Final R indices**	all data	R1 = 0.0995, wR2 = 0.0975
**Function minimized**	$\Sigma\, w(F_o^2 - F_c^2)^2$		I>2σ(I)	R1 = 0.0483, wR2 = 0.0825
**Goodness-of-fit on F^2**	0.996	**Weighting scheme**	$w=1/[\sigma^2(F_o^2)+(0.0217P)^2]$	
**Largest diff. peak and hole [e Å^{-3}]**	0.57/-0.53		where $P=(F_o^2+2F_c^2)/3$	

Di-*tert*-butyl (1a*R**,17a*S**)-1a,2,17,17a-tetrahydrodinaphtho[2,1-b:1',2'-d]oxireno[2,3-h][1,6]diazecine-3,16-dicarboxylate **11'**

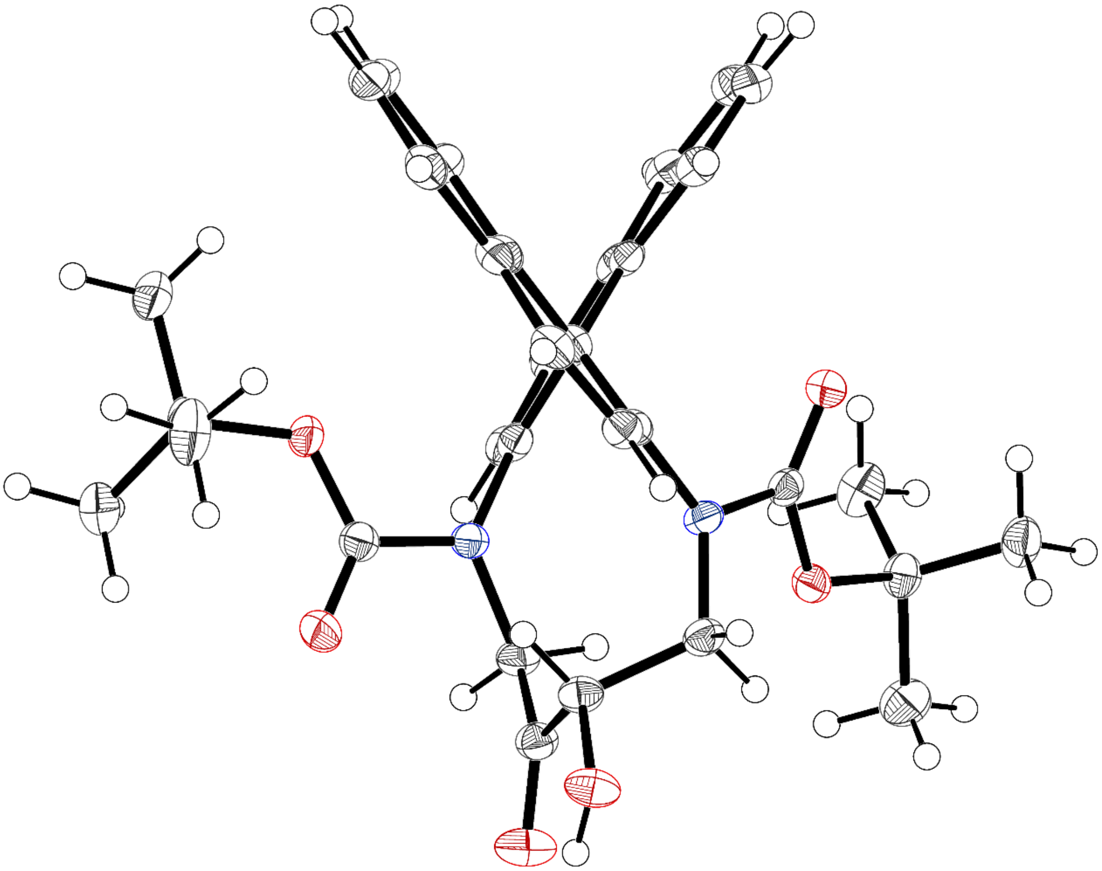

Figure S13 Crystal structure of **11'** (*S*-configuration). C-C Bond precision: 0.0026 Å.

Table S22 Sample and crystal data.of **11'**

**Chemical formula**	$C_{34}H_{36}N_2O_6$	**Crystal system**	monoclinic	
**Formula weight [g/mol]**	568.65	**Space group**	*P21/c*	
**Temperature [K]**	100	**Z**	4	
**Measurement method**	\f and \w scans	**Volume [Å³]**	2913.7(4)	
**Radiation (Wavelength [Å])**	MoKα (λ = 0.71073)	**Unit cell dimensions [Å] and [°]**	15.6315(14)	90
**Crystal size / [mm³]**	0.1 × 0.05 × 0.03		16.9124(14)	111.746(3)
**Crystal habit**	clear colourless		11.8661(9)	90
**Density (calculated) / [g/cm³]**	1.296	**Absorption coefficient / [mm⁻¹]**	0.089	
**Abs. correction Tmin**	0.7047	**Abs. correction Tmax**	0.746	
**Abs. correction type**	multiscan	**F(000) [e⁻]**	1208	

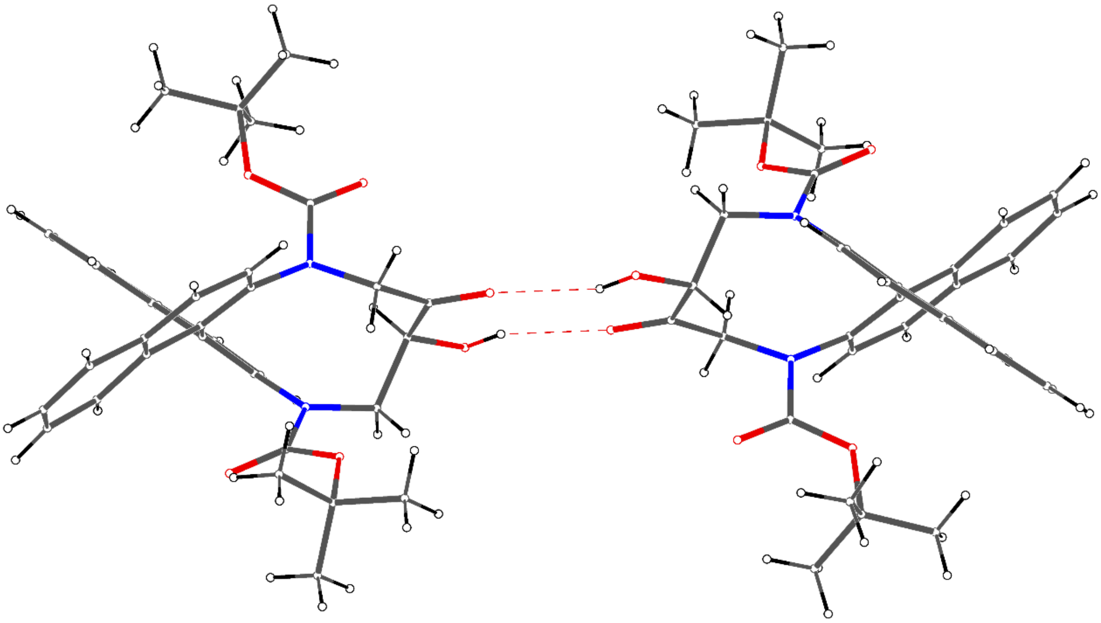

Figure S14 Illustration of moderate intermolecular interactions O-H—O between two molecules of **11’**.

Table S23 Data collection and structure refinement of **11’**.

**Index ranges**	$-18 \leq h \leq 18$, $-20 \leq k \leq 20$, $-14 \leq l \leq 14$	**Theta range for data collection [°]**	4.412 to 50.698	
**Reflections number**	86244	**Data / restraints / parameters**	5330/0/386	
**Refinement method**	Least squares	**Final R indices**	all data	R1 = 0.0565, wR2 = 0.1001
**Function minimized**	Σ w(F_o^2 - F_c^2)^2		I>2σ(I)	R1 = 0.0412, wR2 = 0.0905
**Goodness-of-fit on F^2**	1.052	**Weighting scheme**	w=1/[σ^2(F_o^2)+(0.0360P)^2 +1.5775P]	
**Largest diff. peak and hole [e Å^{-3}]**	0.20/-0.22		where P=(F_o^2+2F_c^2)/3	

Di-*tert*-Butyl (1a*R**,17a*R**)-1a,2,17,17a-tetrahydrodinaphtho[2,1-b:1',2'-d]oxireno[2,3-h][1,6]diazecine-3,16-dicarboxylate **12**

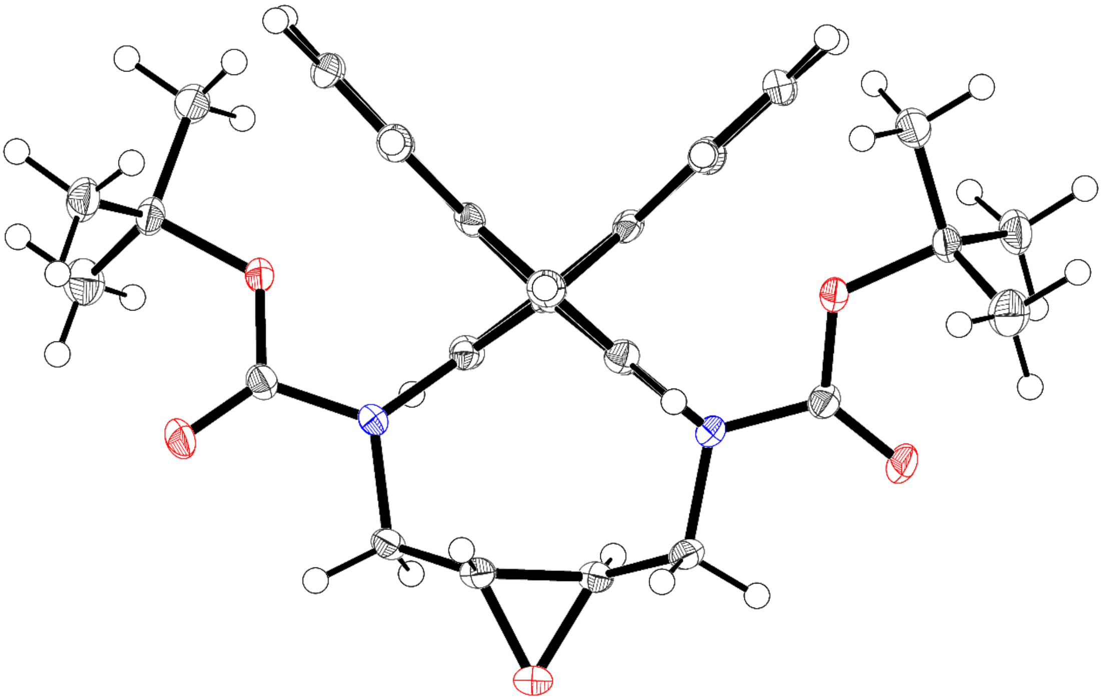

Figure S15 Crystal structure of **12** (*S*-configuration). C-C Bond precision: 0.0017 Å.

Table S24 Sample and crystal data of **12.**

**Chemical formula**	$C_{34}H_{36}N_2O_{4.88}$	**Crystal system**	monoclinic	
**Formula weight [g/mol]**	550.65	**Space group**	*C2/c*	
**Temperature [K]**	100	**Z**	4	
**Measurement method**	\f and \w scans	**Volume [Å^3]**	2892.5(4)	
**Radiation (Wavelength [Å])**	CuKα (λ = 1.54178)	**Unit cell dimensions [Å] and [°]**	19.4742(11)	90
**Crystal size / [mm^3]**	0.287 × 0.218 × 0.189		13.0437(11)	125.810(3)
**Crystal habit**	clear colourless		14.0412(12)	90
**Density (calculated) / [g/cm^3]**	1.264	**Absorption coefficient / [mm^{-1}]**	0.679	
**Abs. correction Tmin**	0.5543	**Abs. correction Tmax**	0.7536	
**Abs. correction type**	multiscan	**F(000) [e$^-$]**	1172	

Table S25 Data collection and structure refinement of **12**.

**Index ranges**	$-24 \leq h \leq 23$, $-16 \leq k \leq 16$, $-17 \leq l \leq 17$	**Theta range for data collection [°]**	8.792 to 145.026	
**Reflections number**	21637	**Data / restraints / parameters**	2848/0/190	
**Refinement method**	Least squares	**Final R indices**	all data	R1 = 0.0393, wR2 = 0.0977
**Function minimized**	Σ w(F_o^2 - F_c^2)^2		I>2σ(I)	R1 = 0.0374, wR2 = 0.0958
**Goodness-of-fit on F^2**	1.065	**Weighting scheme**	w=1/[σ^2(F_o^2)+(0.0461P)^2 +2.2674P]	
**Largest diff. peak and hole [e Å^{-3}]**	0.32/-0.28		where P=(F_o^2+2F_c^2)/3	

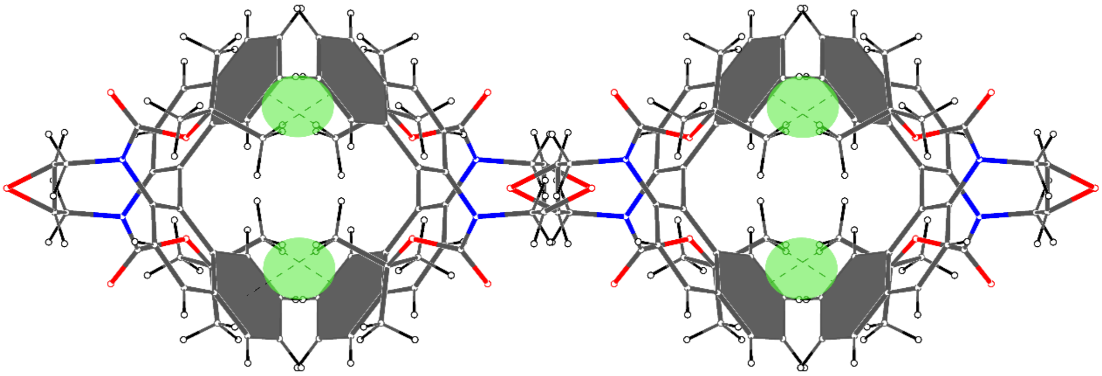

Figure S16 Packing of **12** along axis c. Green highlighted visualization of C-H—PI Ring intermolecular interactions

7,8,9,10,11,12-Hexahydrodinaphtho[2,1-b:1',2'-d][1,6]diazecine **21**

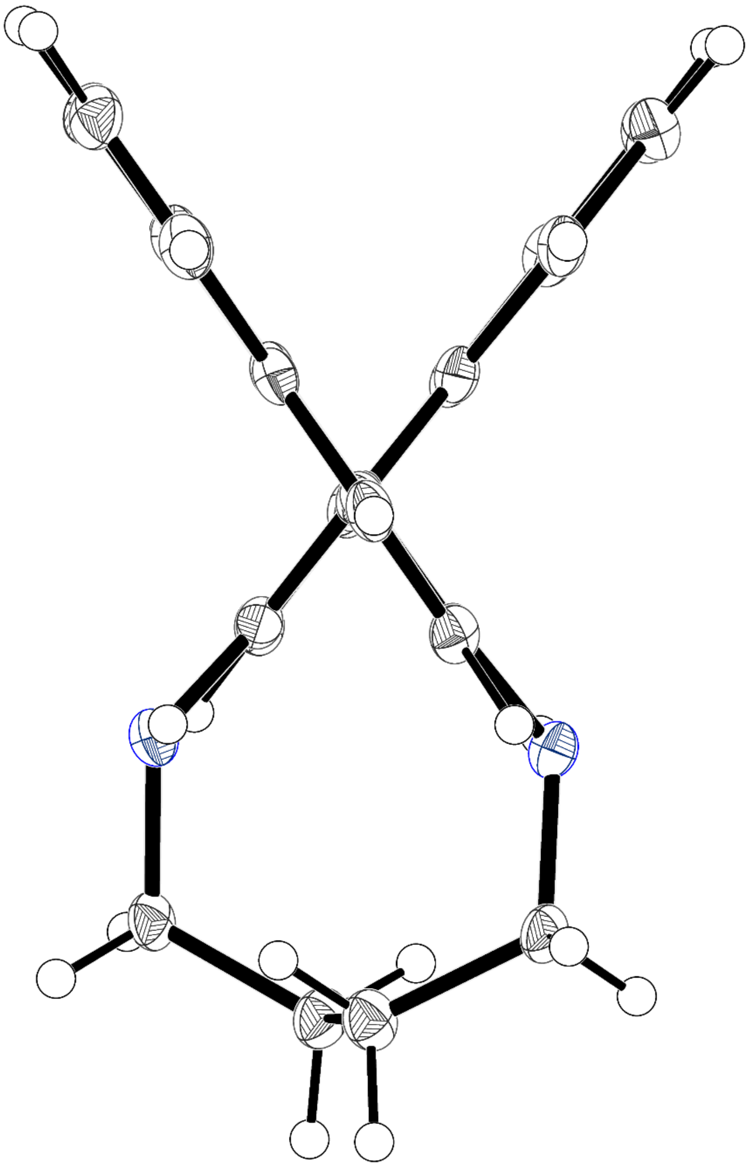

Figure S17 Crystal structure of **21** (*S*-configuration). C-C Bond precision: 0.0049 Å.

Table S26 Sample and crystal data of **21**.

**Chemical formula**	$C_{24}H_{22}N_2$	**Crystal system**	orthorhombic	
**Formula weight [g/mol]**	338.43	**Space group**	*Aea2*	
**Temperature [K]**	100.0	**Z**	4	
**Measurement method**	\f and \w scans	**Volume [Å^3]**	1746.0(3)	
**Radiation (Wavelength [Å])**	MoKα (λ = 0.71073)	**Unit cell dimensions [Å] and [°]**	11.6694(12)	90
**Crystal size / [mm^3]**	0.843 × 0.196 × 0.034		15.4757(14)	90
**Crystal habit**	clear colourless plate		9.6682(7)	90
**Density (calculated) / [g/cm^3]**	1287	**Absorption coefficient / [mm^{-1}]**	0.075	
**Abs. correction Tmin**	0.4602	**Abs. correction Tmax**	0.7460	
**Abs. correction type**	multiscan	**F(000) [e$^-$]**	720.0	

Table S27 Data collection and structure refinement of **21**.

**Index ranges**	-16 ≤ h ≤ 14, -21 ≤ k ≤ 21, -13 ≤ l ≤ 13	**Theta range for data collection [°]**	5.264 to 60.196	
**Reflections number**	6717	**Data / restraints / parameters**	2282/1/118	
**Refinement method**	Least squares	**Final R indices**	all data	R1 = 0.0910, wR2 = 0.2103
**Function minimized**	Σ w(F_o^2 - F_c^2)^2		I>2σ(I)	R1 = 0.0767, wR2 = 0.1924
**Goodness-of-fit on F^2**	1016	**Weighting scheme**	w=1/[σ2(Fo2)+(0.1510P)2]	
**Largest diff. peak and hole [e Å^{-3}]**	0.34/-0.37		where P=(F_o^2+2F_c^2)/3	

[1] Bruker SAINT v8.38A/B & SAINT v7.56/7.68A Copyright © 2005–2019 Bruker AXS.
[2] Sheldrick, G. M. (1996). *SADABS.* University of Göttingen, Germany.
[3] Dolomanov, O. V.; Bourhis, L. J.; Gildea, R.J; Howard, J. A. K.; Puschmann, H. OLEX2, *J. Appl. Cryst.* **2009**, *42*, 339–341.
[4] Huebschle, C. B.; Sheldrick, G. M.; Dittrich, B. ShelXle: a Qt graphical user interface for SHELXL, *J. Appl. Cryst.* **2011**, *44*, 1281–1284.
[5] Sheldrick, G. M. (2015). *SHELXS v 2016/4* University of Göttingen, Germany.
[6] Sheldrick, G. M. (2015). *SHELXL v 2016/4* University of Göttingen, Germany.
[7] Spek, A. L. *Acta Cryst.* **2009**, *D65*, 148–155.
